# Enantiospecific Synthesis of Aniline-Derived Sulfonimidamides

**DOI:** 10.1021/acs.orglett.3c02132

**Published:** 2023-07-25

**Authors:** Dong-Dong Liang, Natassa Lional, Bas Scheepmaker, Muthusamy Subramaniam, Guanna Li, Fedor M. Miloserdov, Han Zuilhof

**Affiliations:** ‡ Laboratory of Organic Chemistry, Wageningen University, Stippeneng 4, 6708 WE Wageningen, Netherlands; § Department of Chemistry, Capital Normal University, Beijing 100048, People’s Republic of China; ∥ Biobased Chemistry and Technology, Wageningen University, Bornse Weilanden 9, 6708 WG Wageningen, Netherlands; ⊥ Institute for Molecular Design and Synthesis, School of Pharmaceutical Science & Technology, Tianjin University, Tianjin 300072, People’s Republic of China

## Abstract

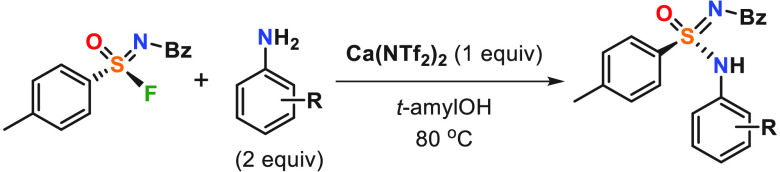

Reaction of sulfonimidoyl fluorides with anilines and Ca(NTf_2_)_2_ results in the formation of chiral sulfonimidamides. The reaction proceeds with inversion of the stereocenter at a sulfur atom. Enantiospecificity of the reaction was observed for all studied non-heterocyclic anilines. Combined experimental and computational mechanistic studies highlight chelate-type coordination of the sulfonimidoyl group to Ca(NTf_2_)_2_ and the formation of a S_N_2-like transition state, in which leaving F^–^ coordinates with the Ca^2+^ ion.

The sulfonamide moiety is fundamental in medicinal chemistry, starting from sulfonamide antibiotics in the 1930s.^1^ Sulfadiazine, sulfadoxine, and sulfamethoxazole are included in the World Health Organization (WHO) model list of essential medicines (Figure 1A),^2^ and, e.g., tipranavir (Figure 1A) is an important sulfonamide drug that is used for human immunodeficiency virus (HIV) treatment.^3^ Sulfonimidamides (Figure 1B) are bioisosteric analogues of sulfonamides,^4^ in which one of the oxygens is replaced by an imine functional group. Such a modification introduces an additional chiral handle that allows for further variation of molecular structures in three dimensions (3D), potentially leading to more active and less toxic compounds.^5^ Such a sulfonamide-to-sulfonimidamide fragment substitution was recently shown to result in decreased cytotoxicity and improved antibacterial properties.^6^

**Figure 1:**
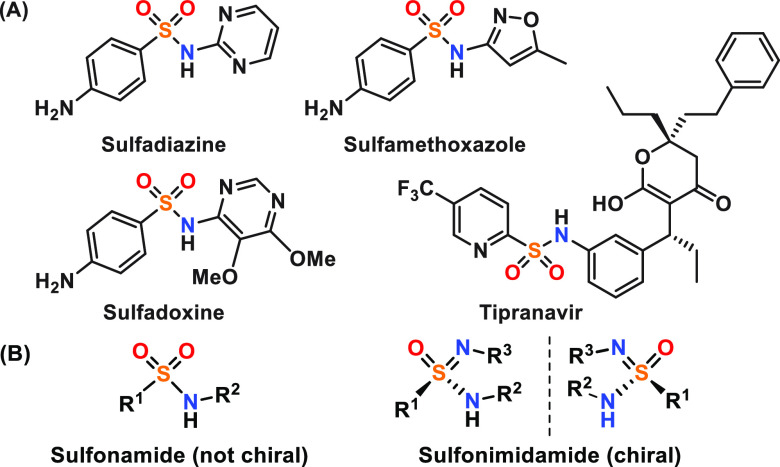
(A) Sulfonamide-containing drugs and (B) general structure of sulfonamides and sulfonimidamides.

Sulfonimidamides possess an intrinsic chirality center at a tetrahedral sulfur atom.^7^ To access the wide chemical space of sulfonimidamides, it is necessary to develop efficient methods to synthesize these molecules in a stereo-controlled manner. In 2010, Bolm and co-workers^8a^ reported the synthesis of chiral unsubstituted sulfonimidamides by enantiospecific nucleophilic substitution of chiral sulfonimidoyl chlorides with ammonia (Figure 2A), and recently, this reaction was extended to a broad range of aromatic and aliphatic amines.^8b^ Bull’s group^9^ used sulfur fluoride exchange (SuFEx) to accomplish the stereoselective synthesis of sulfonimidamides based on aliphatic amines. The use of LiBr as a fluoride ion scavenger was crucial to preventing racemization of starting sulfondimidoyl fluorides. Recently, Willis and co-workers reported the synthesis of chiral sulfonimidamides using organocatalytic benzylation of prochiral sulfonimidamide anions (Figure 2A).^10^ To the best of our knowledge, the enantioselective synthesis of aniline-derived sulfonimidamides (R^2^ = Ar; Figure 1B) from sulfonimidoyl fluorides was not accomplished. Such methods are highly desirable, particularly as a result of the importance of *N*-aryl-substituted sulfonamides for medicinal chemistry (Figure 1A), the wide availability of anilines, and the substantially higher stability of sulfur(VI) fluorides to hydrolysis in comparison to sulfur(VI) chlorides.

**Figure 2:**
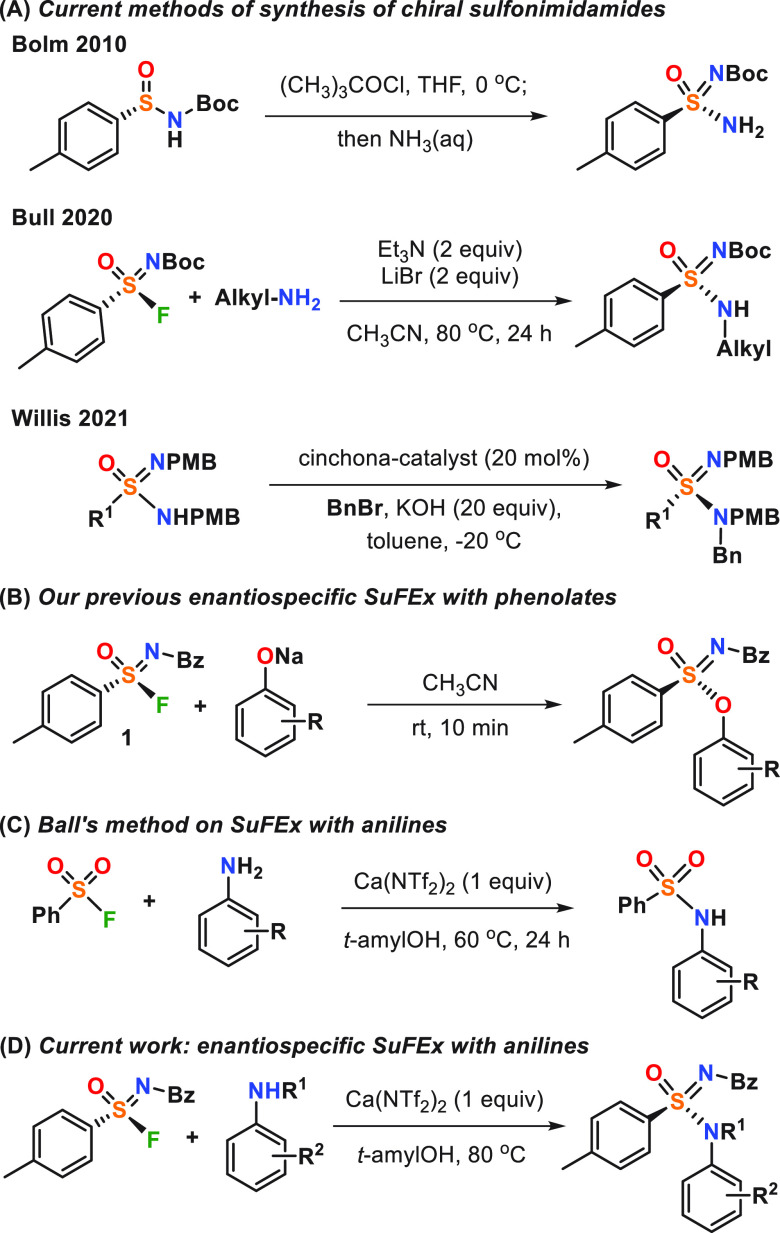
(A) Current methods of the synthesis of chiral sulfonimidamides, (B) our previous enantiospecific SuFEx with phenolates, (C) Ball’s method on SuFEx with anilines, and (D) current work for enantiospecific SuFEx with anilines.

The lack of enantioselective syntheses of aniline-derived sulfonimidamides from sulfur(VI) fluorides is not surprising considering the low nucleophilicity of anilines in comparison to phenolates and alkyl amines. In line with our wider interest to further develop S(VI) exchange chemistry,^11^ we thus aimed to develop methods to achieve this transformation.

In line with the previous studies, our initial attempts to react sulfonimidoyl fluoride **1** with aniline under conditions reported by Bull and co-workers^9^ were not successful. Similarly, the conditions that we previously used for the first enantiospecific phenolate-based SuFEx reaction^11b,11e,11f^ (Figure 2B) were not working for aniline-derived anions (see the Supporting Information for details). Next, we turned our attention to an alternative approach related to Ball’s use of Ca(NTf_2_)_2_ to activate sulfonyl fluorides (Figure 2C)^12^ and investigated this systematically with the aim to develop a SuFEx-based enantioselective method for the synthesis of aniline-derived sulfonimidamides (Figure 2D).

We started our investigation with optimization of the reaction conditions. After extensive variation of different bases, solvents, and Lewis acids, we were able to isolate product **2a** in 96% yield [>99% high-performance liquid chromatography (HPLC) yield] and >99% enantiomeric excess (ee), starting from chiral **1**. Similar to Ball and co-workers,^12a^ we found that Ca(NTf_2_)_2_ is crucial for the reaction success, while *t*-amyl alcohol is the most suitable solvent, and a second equivalent of aniline was the best base to minimize the side hydrolysis (Table 1; see the Supporting Information for details).

**Table 1:**
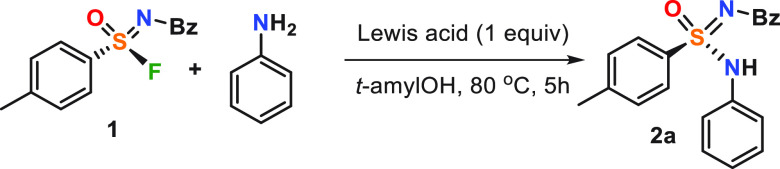
Lewis Acid Optimization^a^

entry	Lewis acid	solvent	yield (%)^b^
1	none	*t*-amylOH	nr
2	Ca(NTf_2_)_2_	*t*-amylOH	>99
3	Ca(OTf)_2_	*t*-amylOH	10
4	Mg(NTf_2_)_2_	*t*-amylOH	50
5	Ba(NTf_2_)_2_	*t*-amylOH	72
6	Li(NTf_2_)	*t*-amylOH	13
7	Zn(NTf_2_)_2_	*t*-amylOH	3
8	B(C_6_F_5_)_3_	*t*-amylOH	3
9	Ca(NTf_2_)_2_	*t*-BuOH	76
10	Ca(NTf_2_)_2_	CH_3_CN	55
11	Ca(NTf_2_)_2_	toluene	53

a Reaction conditions: sulfonimidoyl fluoride (1 equiv), aniline (2 equiv), Lewis acid (1 equiv), *t*-amylOH (0.2 M), 80 °C, and 5 h.

b Yield was determined by HPLC.

With optimal reaction conditions in hand, we tested the substrate scope, with an attention on the influence of steric and electronic properties of anilines on the reaction yields and stereospecificity. To our delight, electron-donating groups (OMe and SMe) in the *ortho* and *para* positions on the aromatic ring as well as the −NHMe moiety as a reactive group were well-tolerated (**2b**–**2f**), and reaction proceeded with good to excellent yields of 70–90% and >99% ee. Anilines with mild to reasonable electron-withdrawing groups (*m*-CF_3_, *m*-OH, *m*-NO_2_, *p*-I, and *p*-CO_2_Me) could also be involved in the reaction, leading to target compounds **2h**–**2l** in 54–90% yield and >99% ee, although 3 equiv of aniline was required to achieve full conversion of compound **1**. The reaction proceeds with an inversion of configuration at the sulfur atom, which was confirmed by X-ray crystal structures of (*S*)-**2b** and (*S*)-**2d**. Several reaction limitations were also observed. This SuFEx reaction did not work with highly electron-deficient *p*-NO_2_ aniline **2m** (Figure 3). In addition, for both 3-aminophenol and 4-aminophenol, which can react via the N and O atoms, no formation of the O-SuFEx product was observed (**2g** and **2i**).

**Figure 3:**
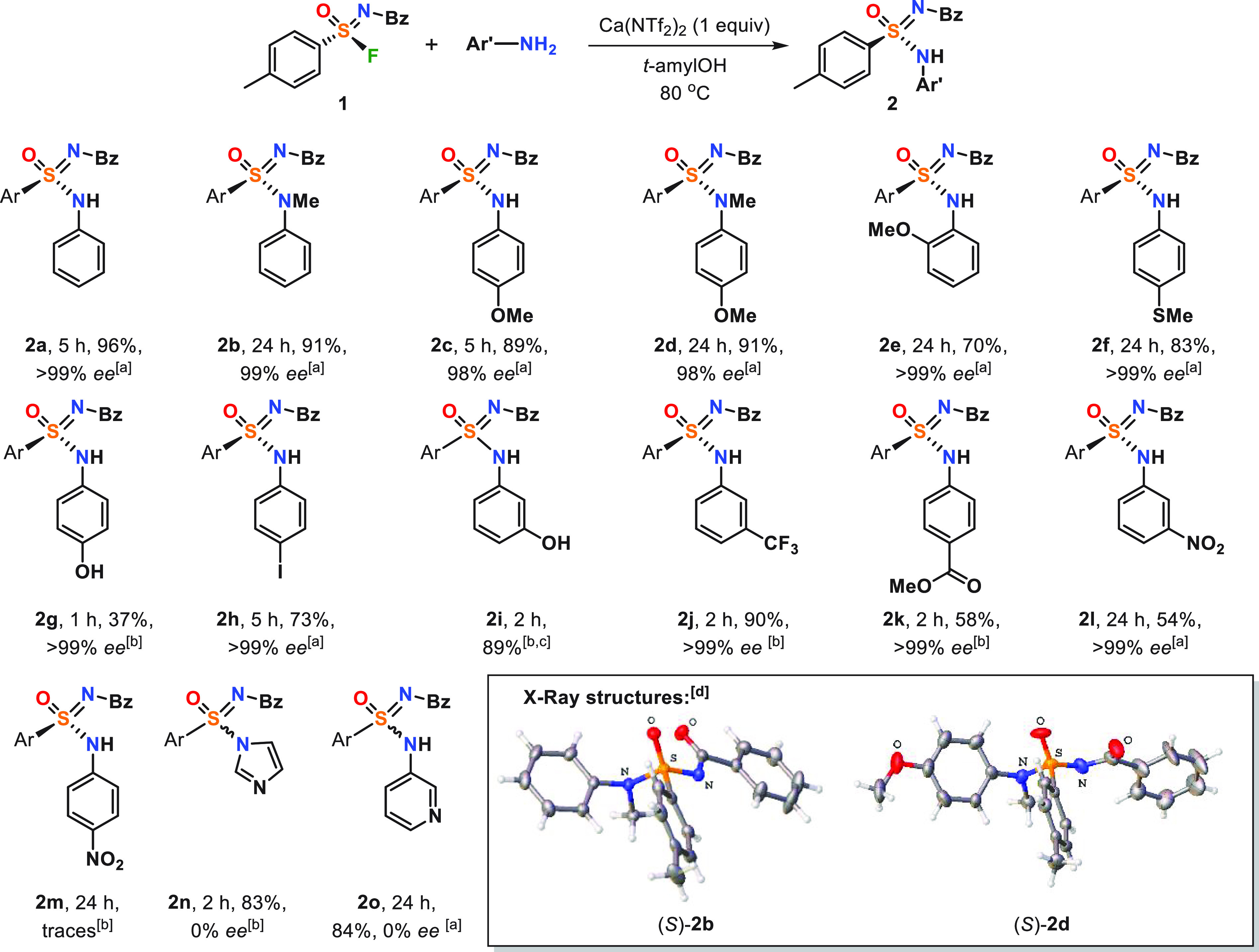
Scope of the enantiospecific SuFEx reaction. Ar, 4-tolyl; Bz, benzoyl. An enantiospecific reaction was performed on a 0.126 mmol scale using (*R*)-**1** (>99% ee). [a] Compound **1** (1 equiv), aromatic amine (2 equiv), and Ca(NTf_2_)_2_ (1 equiv) in *t*-amyl alcohol (0.2 M) at 80 °C. [b] Compound **1** (1 equiv), aromatic amine (3 equiv), and Ca(NTf_2_)_2_ (1 equiv) in *t*-amyl alcohol (0.2 M) at 80 °C. [c] ee was not determined as a result of the inability to separate enantiomers on chiral HPLC. [d] Molecular structures of (*S*)-**2b** and(*S*)-**2d** are shown with ellipsoids being represented at 50% probability, and solvent molecules are omitted for clarity.

The reaction could also tolerate heteroaromatic amines, such as imidazole and 3-aminopyridine, leading to products **2n** and **2o** in a high yield (>80%). However, these reactions were found to not be enantiospecific, and only the racemic product was isolated. Most likely, the racemization is caused by degenerate nucleophilic substitution in the product molecule, in which imidazole or 3-aminopyridine is additionally activated by the present Lewis acid [Ca(NTf_2_)_2_] or protonation by ArNH_3_^+^ produced in the reaction. A similar nucleophilic substitution of *N*-methyl imidazole was previously reported by Grygorenko et al.^13^

To further understand the role of the Lewis acid in this reaction, quantum chemical calculations on the reaction of sulfonimidoyl fluoride **1** and aniline (Figure 4) were performed at the ωB97XD/6-311+G(d,p) level of theory. Here, the bulk properties of the solvent (*t*-amyl alcohol) were represented by a dielectric continuum [polarizable continuum model (PCM) solvent model], while any specific role of solvent molecules was taken into account by explicit addition of 2 *t*-BuOH molecules (mimicking *t*-amyl alcohol for computational means) in our computations. In such a solvent, Ca(NTf_2_)_2_ is readily solvated by two solvent molecules, but upon addition of compound **1**, this coordination is replaced by coordination of Ca(NTf_2_)_2_ to compound **1**, which was found to be 4.9 kcal/mol (Gibbs free energy) more favorable (Table S11 of the Supporting Information). Compound **1** interacts with Ca(NTf_2_)_2_ via a sulfonyl oxygen atom and a carbonyl oxygen atom, forming a stable six-membered chelate ring. This coordination mode contrasts with findings of Ogba, Ball, and co-workers on the activation of sulfonyl fluorides (RSO_2_F) with the Ca(NTf_2_)_2_/DABCO system. They observed that Ca(NTf_2_)_2_ coordinates to RSO_2_F through a sulfonyl group, with the corresponding RSO_2_F···Ca(NTf_2_)_2_ adduct being 3 kcal/mol less stable than the corresponding tetrahydrofuran (THF)···Ca(NTf_2_)_2_ complex (computed with explicit and implicit THF solvation).^14^ In their case, in contrast to that under the current study for sulfonimidoyl fluorides, no chelation was observed. For our case, the computationally predicted formation of a stable coordination adduct between compound **1** and Ca(NTf_2_)_2_ was also confirmed experimentally. The treatment of compound **1** with 1 equiv of Ca(NTf_2_)_2_ in *t*-amylOH results in the significant broadening of the ^19^F nuclear magnetic resonance (NMR) signal of sulfonimidoyl fluoride and its shift from δ 60.2 to 67.6 ppm.

**Figure 4:**
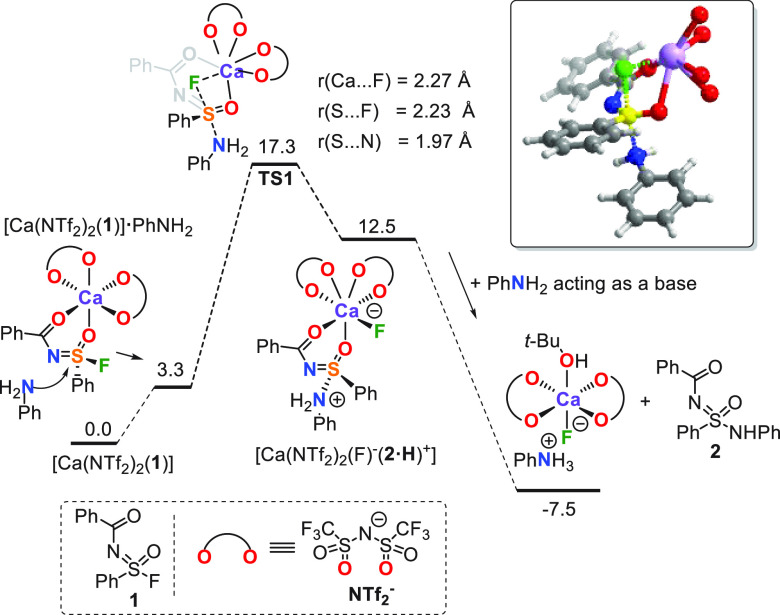
Computed reaction profile (Gibbs free energies, kcal/mol, with *T* = 353 K and *p* = 1 atm) for the reaction of [Ca(NTf_2_)_2_(**1**)] with aniline. (Inset) Geometry of transition state **TS1**, with all NTf_2_ groups being truncated.

Computational studies suggest the following mechanism: the addition of aniline to this [Ca(NTf_2_)_2_(**1**)] complex results in the formation of an adduct [Ca(NTf_2_)_2_(**1**)]·PhNH_2_, which is calculated to be 3.3 kcal/mol higher in energy than separated [Ca(NTf_2_)_2_(**1**)] and PhNH_2_ (Figure 4). In this complex, the aniline–S interaction allows for more readily of a shift of the F atom toward the Ca^2+^ ion, effectively inducing the overall nucleophilic attack of aniline at the S(VI) center. This reaction takes place with the inversion of configuration via a S_N_2-type mechanism with a transition state (**TS1**) at 17.3 kcal/mol, relative to the original reaction complexes. Such calculated activation energy is in line with the required thermal activation that was observed for the whole range of anilines (80 °C; Figure 3), but it would, in fact, also be compatible with a slow reaction at room temperature (*vide infra*).

The TS has a late character, which is manifested by a significant elongation of the S···F bond to 2.23 Å (∑covalent radii = 1.62 Å),^15^ shortening of *r*(S···N) to 1.97 Å, and a strong Ca–F interaction (2.27 Å) at a seven-coordinate Ca center (Table S12 of the Supporting Information). The core geometry of our TS resembles that computed by Ogba, Ball, and co-workers for the corresponding reaction with sulfonyl fluorides.^14^ The coordination of the F atom to the Ca center is accompanied by elongation of the calcium triflimide Ca–O bonds {from 2.39 Å in [Ca(NTf_2_)_2_(**1**)] to 2.46 Å in transition state **TS1**}.

Following this, transition state **TS1** toward product formation (by intrinsic reaction coordinate studies) leads to an intermediate [Ca(NTf_2_)_2_(F)^−^(**2**·H)^+^] that lies at 12.5 kcal/mol relative to starting [Ca(NTf_2_)_2_(**1**)]. This intermediate can be viewed as a complex of N-protonated product **2** and a [Ca(NTf_2_)_2_(F)]^−^ anion. Subsequent deprotonation with another 1 equiv of aniline and release of product **2** is a virtually barrierless process, yielding product **2** and a calcium triflimide complex at −7.5 kcal/mol. The gentle driving force of this reaction thus precludes extensive heat development during the reaction, making it also amenable to scale up if required. However, the computed activation energy Δ*G*^⧧^ in combination with the calculated driving force Δ*G* would suggest that the reaction should also go to near completion at room temperature, which it did not under the conditions described above.

To unravel this, we examined, among other things, the ability of product **2** to compete with compound **1** for coordination to Ca(NTf_2_)_2_. Computationally, we found that [Ca(NTf_2_)_2_(**2**)] is 6.3 kcal/mol more stable than [Ca(NTf_2_)_2_(**1**)], suggesting that the sulfonimidamide product **2** will substitute for compound **1** at the Ca(NTf_2_)_2_ Lewis acid, inhibiting the reaction (Table S11 of the Supporting Information). In line with that, the monitoring of a typical reaction of compound **1** with 1 equiv of Ca(NTf_2_)_2_ and 2 equiv of aniline showed autoinhibition: the reaction proceeds at room temperature to 34% yield after 5 h and virtually stops after 24 h at a 63% yield of compound **1**. To reach the full conversion reaction, heating was required at 80 °C. To prevent autoinhibition, 2 equiv of Ca(NTf_2_)_2_ was used (the extra equivalent thus added to bind to the produced product **2**). The reaction then proceeded, for all examples investigated, readily at room temperature, with 95% conversion reached within 5 h, confirming our hypothesis.

The use of 2 equiv of Lewis acid might be advantageous for less reactive substrates, for which a full conversion is difficult to reach, for substrates with other temperature-sensitive functionalities and when significant amounts of side hydrolysis are observed. However, this would come at a cost of the use of another equivalent of Ca(NTf_2_)_2_. We also tested the room-temperature reaction conditions with the imidazole and 3-amino pyridine substrates **2n** and **2o**, for which the 80 °C reactions led to product racemization. While these reactions indeed reached the full conversion of (*R*)-**1** at room temperature within 2 h, the products were still produced in the racemic form.

In conclusion, we have demonstrated that sulfonimidoyl fluorides can be reacted in an enantiospecific manner with anilines upon activation by Ca^2+^ ions, leading to chirality at sulfur analogues of sulfonamides. Mechanistic studies show a facile coordination of the sulfonimidoyl moiety to Ca^2+^ with the formation of a six-membered chelate, leading to a relatively low-energy TS and a gentle overall driving force. Further studies on the realization of enantioselective catalytic versions of this transformation will be pursued by our group.

## Data Availability

The data underlying this study are available in the published article and its online Supporting Information.

## Supporting Information Available

Experimental procedures, compound characterization, NMR spectra, HPLC chromatograms, and crystallographic and computational data (PDF)
