# Supplementary material for: Enantiospecific Synthesis of Aniline-Derived Sulfonimidamides
Source: Org Lett. 2023 Jul 25;25(30):5666–70. doi: 10.1021/acs.orglett.3c02132 (PMC10407922; doi:10.1021/acs.orglett.3c02132)
Supplement: Supplementary file 1 — ol3c02132_si_001.pdf [file ol3c02132_si_001.pdf]

Supporting information

Enantiospecific Synthesis of Aniline-Derived Sulfonimidamides

Dong-Dong Liang,[1,2]‡ Natassa Lional,[1]‡ Bas Scheepmaker,[1] Muthusamy Subramaniam,[1] Guanna Li,[1,3] Fedor M. Miloserdov,[1]* Han Zuilhof[1,4]*

1) Laboratory of Organic Chemistry, Wageningen University, Stippeneng 4, 6708WE Wageningen, The Netherlands

2) Department of Chemistry, Capital Normal University, Beijing, 100048, China

3) Biobased Chemistry and Technology, Wageningen University, Bornse Weilanden 9, 6708WG Wageningen, The Netherlands

4) School of Pharmaceutical Science and Technology, Tianjin University, Tianjin, 300072, China

‡These authors contributed equally.

*Corresponding authors emails: fedor.miloserdov@wur.nl & han.zuilhof@wur.nl

Table of Contents

General information and methods S-3
Reactions with anilides S-4
  Reactions with benzanilide S-4
  Reactions with acetanilide S-5
Reactions with aniline, optimization of the reaction conditions S-7
  General procedure S-7
  LC calibration for quantitative analysis S-7
  Optimization of base S-8
  Optimization of solvent S-9
  Optimization of temperature S-10
  Optimization of reaction concentration S-11
  Optimization of reaction time S-12
  Discussion S-12
N-SuFEx reactions of racemic 1 with aromatic amines S-13
  Experimental data S-14
  NMR spectra S-22
N-SuFEx reactions between chiral *(R)*-1 and aromatic amines. S-40
  HPLC data S-41
X-Ray crystallographic data of (*S*)-2b and (*S*)-2d S-52
Computational studies S-55
References S-65

General information and methods

All commercial chemicals were used as received and stored under argon. Racemic sulfonimidoyl fluoride **1** was synthesized according to the literature,[1] while chiral sulfonimidoyl fluoride **1** was synthesized according to the literature.[2] Reagents were used without further purification unless otherwise noted. Unless otherwise noted, all reactions were performed using glassware without further preparation. Certain reactions were carried out in anhydrous conditions. This utilized oven-dried glassware which was heated in an oven above 100 °C for at least 5 h or which was dried under vacuum with a heat gun (T > 200 °C), under oxygen-free and water-free conditions. After weighing any solids, the glassware was connected to a Schlenk line, and then placed under vacuum and flushed with nitrogen gas (repeated 3 times). Liquids were added via syringe through a rubber septum. An oil bath was used as the heat source. Solvent abbreviations are: tetrahydrofuran (THF), ethyl acetate (EtOAc), dichloromethane (DCM), isopropyl alcohol (IPA), methanol (MeOH), *tert*-amyl alcohol (*t*-amylOH), petroleum ether 40-60 (P.E.). Solvents were used as received without any distillation with the exception of *t*-amylOH which was distilled over sodium before use. Flash column chromatography was performed using a Biotage® system, SiliCycle® precast silica columns (200−300 mesh or 300−400 mesh) and silica gel 40-63 µm. TLC analysis was performed on pre-coated, alumina-backed silica gel plates. TLC plates were analyzed by UV fluorescence (254 nm) or I_2 stain. ^{1}H NMR and ^{13}C NMR spectra were recorded on a Bruker Avance 400 MHz spectrometer at 298 K. Structural assignments were made with additional information from gCOSY, gHSQC, and gHMBC experiments. The chemical shifts are listed in ppm on the δ = scale and coupling constants were recorded in Hertz (Hz). Chemical shifts are calibrated relative to the signals of corresponding non-deuterated solvents ($CHCl_3$: δ = 7.26 ppm for ^{1}H and 77.16 ppm for ^{13}C, DMSO: δ = 2.50 ppm for ^{1}H and 39.52 ppm for ^{13}C). Abbreviations are used in the description of NMR data as follows: chemical shift (δ = ppm), multiplicity (s = singlet, d = doublet, t = triplet, q = quartet, dd = doublet of doublet and m = multiplet), coupling constant (*J*, Hz). High-resolution mass spectra (HRMS) were measured on a QTOF micro spectrometer using electrospray ionization (ESI) in positive mode (ESI^+) or in negative mode (ESI^-). Enantioselectivity was monitored using an HPLC Chiralpak IA column. A reference, racemic product was synthesized to determine the column conditions for baseline separation. Optimization of reaction conditions was monitored using Agilent 1290 Infinity UHPLC equipped with Zorbax SB C-18 HPLC column for analysis of reaction outcome. The column conditions for baseline separation were found to be H_2O:MeCN, (50:50 v/v) + 0.1% formic acid, flow rate 0.6 mL/min, oven temperature 23 °C, detector wavelength 238 nm.

Reactions with anilides

Reactions with benzanilide

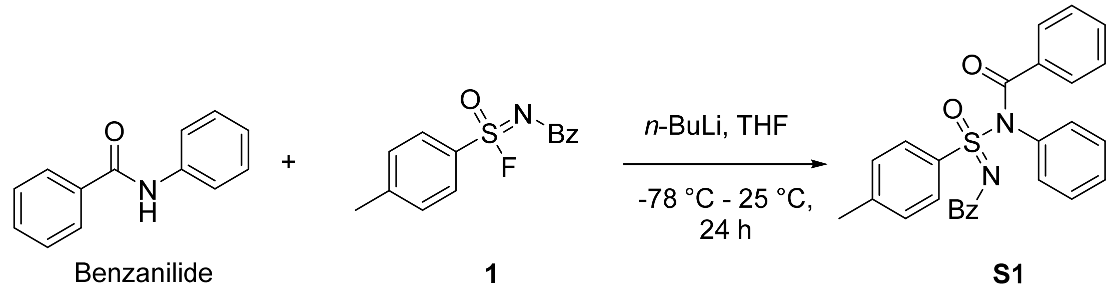

Benzanilide (49.3 mg; 0.25 mmol; 1.09 equiv) was charged into an oven dried 50 mL round bottom flask and flushed with N_2. 10 mL of dry THF was added and the solution was cooled to -78 °C. At this temperature, *n*-BuLi 2.5 M hexanes (0.16 mL; 0.26 mmol; 1.1 equiv) was added dropwise. After 30 min, sulfonimidoyl fluoride **1** (63.9 mg; 0.23 mmol; 1 equiv) was added to the reaction mixture in one portion. The reaction mixture was allowed to warm up to room temperature and the reaction was monitored by TLC at 1, 5 and 24 h. After 24 h, the reaction mixture was quenched with methanol. TLC and HRMS did not show the target product **S1**.

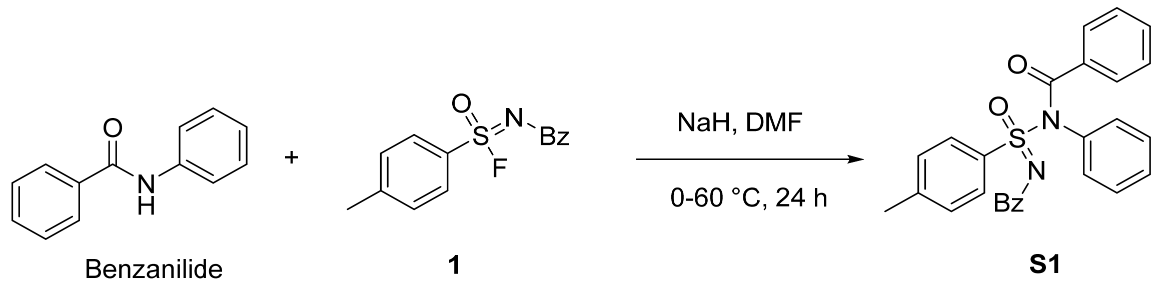

Benzanilide (49.3 mg; 0.25 mmol; 1.09 equiv) was charged into an oven dried 50 mL round bottom flask and flushed with N_2. 10 mL of dry DMF was added and the solution was cooled to 0 °C. At this temperature, sodium hydride 60% dispersion in mineral oil (13.19 mg; 0.33 mmol; 1.4 equiv) was added. After 30 min, sulfonimidoyl fluoride **1** (63.9 mg; 0.23 mmol; 1 equiv) was added to the reaction mixture in one portion. The reaction mixture was allowed to warm up to room temperature and the reaction was monitored by TLC at 1, 5 and 24 h. After 32 h, the reaction mixture was heated to 60 °C for 24 h. TLC and HRMS did not show the target product **S1**.

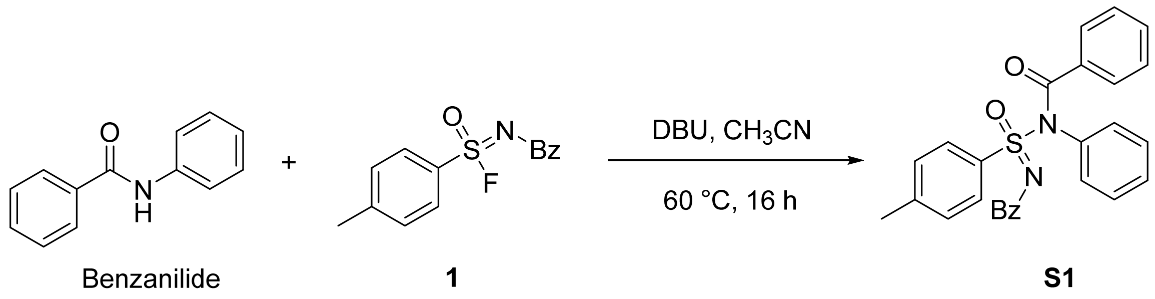

Benzanilide (49.3 mg; 0.25 mmol; 1.09 equiv) was charged into an oven dried 50 mL round bottom flask and flushed with N_2. 10 mL dry acetonitrile was added along with DBU (40 µL; 0.27 mmol; 1.2 equiv). After 30 min, sulfonimidoyl fluoride **1** (63.9 mg; 0.23 mmol; 1 equiv) was added to the reaction mixture in one portion. The reaction mixture was monitored by TLC at 1 and 2 h. After 2 h, the reaction mixture was heated to 60 °C for 16 h. TLC and HRMS did not show the target product. **S1**

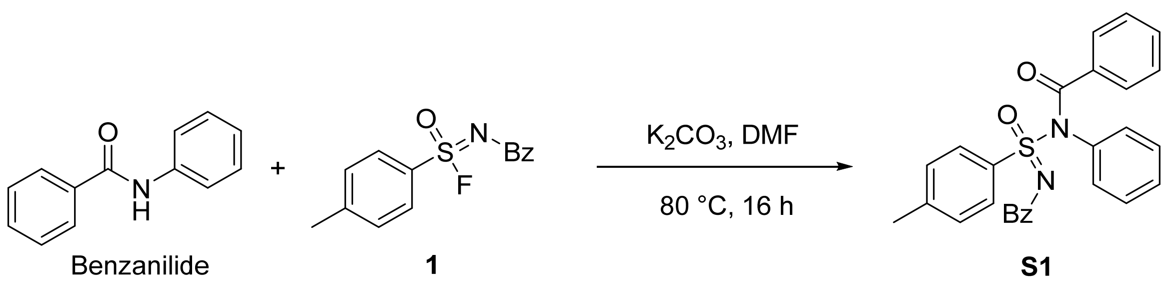

Benzanilide (49.3 mg; 0.25 mmol; 1.09 equiv) was charged into an oven dried 50 mL round bottom flask and flushed with N_2. 10 mL of dry DMF was added along with K_2CO_3 (40 mg, 0.25 mmol; 1.09 equiv). After 30 min, sulfonimidoyl fluoride **1** (63.9 mg; 0.23 mmol; 1 equiv) was added to the reaction mixture in one portion. The reaction mixture was monitored by TLC at 1 and 2 h. After 2 h, the reaction mixture was heated to 80 °C for 16 h. TLC and HRMS did not show the target product **S1**.

Reactions with acetanilide

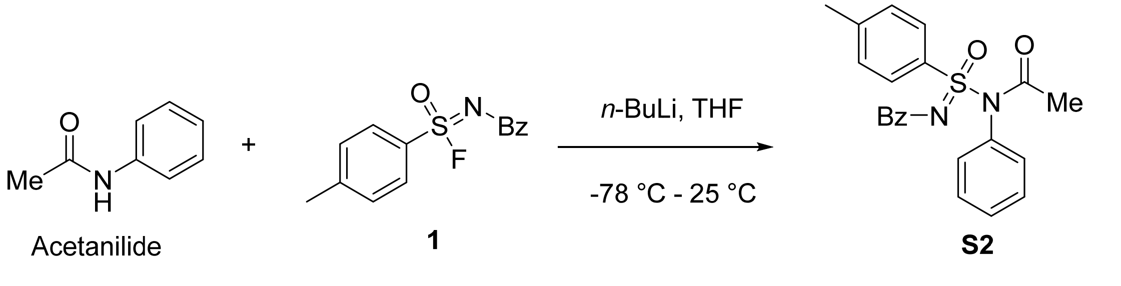

Acetanilide (34 mg; 0.25 mmol; 1.09 equiv) was charged into an oven dried 50 mL round bottom flask and flushed with N_2. 10 mL dry THF was injected then the mixture was cooled to -78 °C. At this temperature, *n*-BuLi (0.16 mL; 0.26 mmol; 1.1 equiv) was added dropwise. After 30 min, sulfonimidoyl fluoride **1** (63.9 mg; 0.23 mmol; 1 equiv) was added to the reaction mixture in one portion. The reaction mixture was allowed to warm up to room temperature and the reaction was monitored by TLC at 1, 5

and 24 h. After 120 h, the reaction mixture was quenched with methanol. TLC and HRMS did not show the target product **S2**.

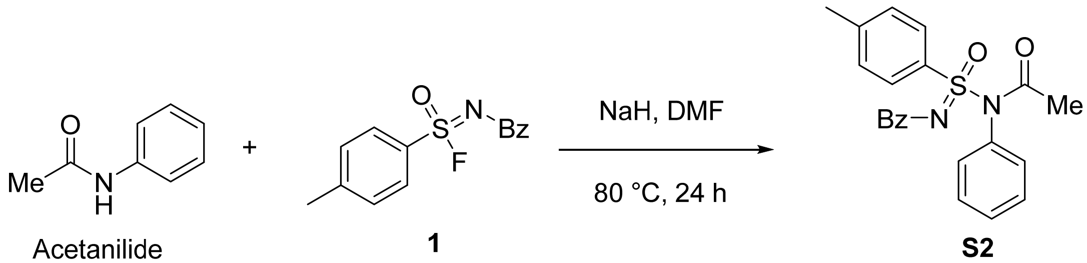

Acetanilide 1 S2

Acetanilide (34 mg; 0.25 mmol; 1.09 equiv) was charged into an oven dried 50 mL round bottom flask and flushed with N_2. 10 mL dry THF was added and the solution was cooled to 0 °C. At this temperature, sodium hydride 60% dispersion in mineral oil (13.19 mg; 0.33 mmol; 1.4 equiv) was added. After 30 min, sulfonimidoyl fluoride **1** (63.9 mg; 0.23 mmol; 1 equiv) was added to the reaction mixture in one portion. The reaction mixture was allowed to warm up to room temperature and the reaction was monitored by TLC at 1, 5 and 48 h. After 100 h, the reaction mixture was heated to 80 °C for 24 h. TLC and HRMS did not show the target product **S2**.

Reactions with aniline, optimization of the reaction conditions

General procedure

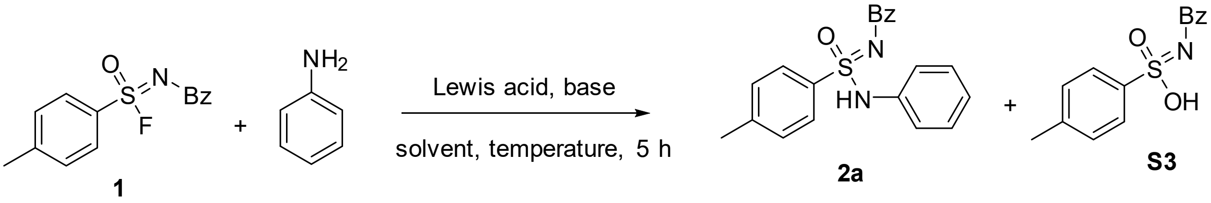

Sulfonimidoyl fluoride **1** (25 mg; 0.09 mmol; 1 equiv), aniline (24 µL; 0.09 mmol; 1 equiv), Lewis acid (0.09 mmol; 1 equiv) and base (0.09 mmol; 1 equiv) were charged into an 4 mL vial. 0.45 mL *t*-amylOH was injected and the reaction mixture was stirred for 5 h.

LC calibration for quantitative analysis

HPLC with a UV-Vis detector was used to determine the yields. Baseline separation was achieved within 5 min. using the conditions as described in general information and methods section. HPLC samples were prepared using 30 µL aliquotes (without work up) that were diluted with 1 mL of a 50:50 (v/v) mixture of water : acetonitrile. A typical HPLC chromatogram is shown in Figure S1. The peak at t_r = 0.264 min corresponds to aniline, the peak at t_r = 0.719 min corresponds to the hydrolysis product **S3**, the peak at t_r = 2.167 min belongs to target product **2a** and the peak at 3.152 min belong to starting material **1**. A calibration was done using an external reference (1,3,5-tribromobenzene). The externally calibrated absorption coefficients are summarized in Table S1.

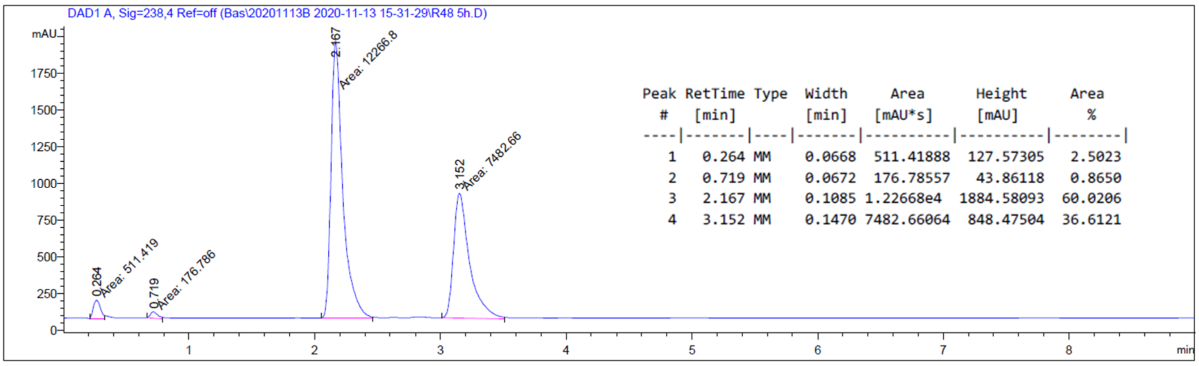

Figure S1. Typical HPLC chromatogram for the reaction optimization.

Table S1. The externally calibrated coefficients for the different reactants and products.

Entry	Compound	Coefficient (μmol^{-1})
1	Sulfonimidoyl fluoride **1**	$1.90*10^6$
2	Hydrolysis byproduct **S1**	$1.55*10^6$
3	Target product **2a**	$2.32*10^6$

Optimization of base

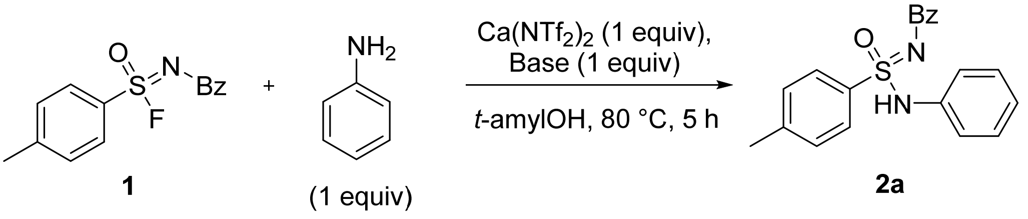

Bases:

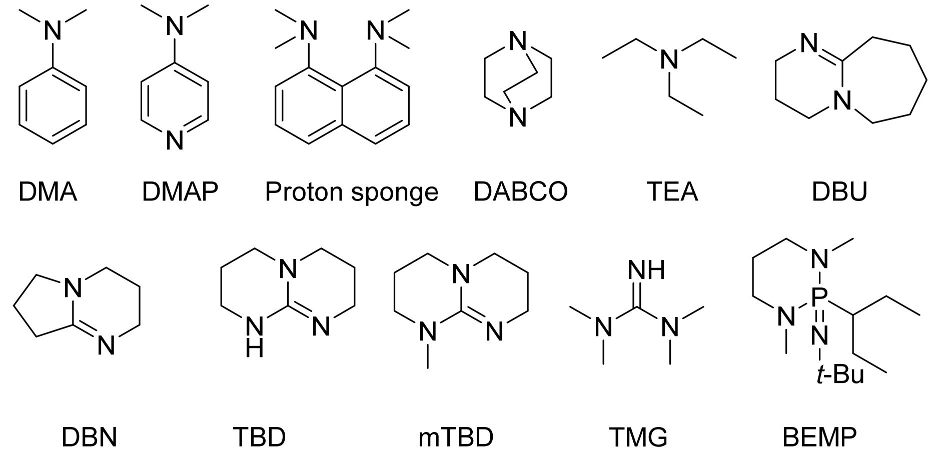

Table S2. Optimization of base.

Entry	Base	Yield (%)	Hydrolysis (%)
1	Aniline	>99	0
2	DMA	64	4
3	DMAP	66	20
4	Proton sponge	56	42
5	DABCO	71	5
6	TEA	52	38
7	DBU	58	42
8	DBN	55	11
9	TBD	53	10
10	mTBD	25	20
11	TMG	61	18
12	BEMP	57	21
13	KOH	43	29
14	K_2CO_3	59	5
15	K_3PO_4	47	2
16[a]	DABCO	n.r.	n.d.

[a] Reaction was performed using THF as solvent and monitored by TLC (Hex (2)/EtOAc (1)); TLC showed only hydrolysis product was formed.

Optimization of solvent

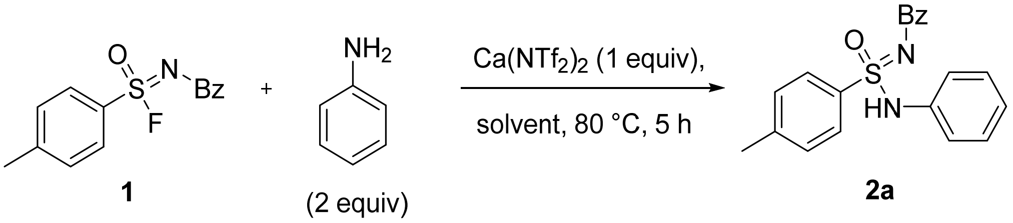

Table S3. The effects of different solvents on the SuFEx reactions between sulfonimidoyl fluoride **1** and aniline.

Entry	Solvent	Yield (%)	Hydrolysis (%)
**1**	*t*-amylOH	>99	0
**2**	*t*-butylOH	76	0
**3**	2-propanol[a]	74	4
**4**	HFIP[a]	49	0
**5**	1,4-dioxane	23	1
**6**	DMF	n.r.	n.r.
**7**	DMSO	n.r.	n.r.
**8**	CH_3CN	55	1
**9**	toluene	53	1
**10**	1,2-dichloroethane	59	0

[a] These experiments were run at 60 °C, due to the boiling point of solvents.

Optimization of temperature

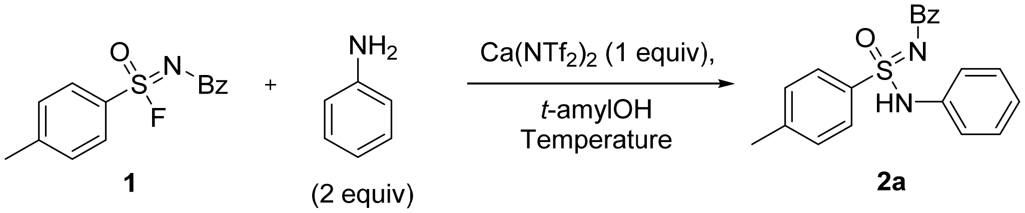

Table S4. The effects of different temperatures on the SuFEx reactions between sulfonimidoyl fluoride **1** and aniline.

Entry	Temp. (°C)	Reaction time (h)	Yield (%)	Hydrolysis (%)
1	20	5	34	2
2	40	5	71	1
3	60	5	83	2
4	80	5	>99	0
5	100	5	88	3
6	20	24	63	2
7	40	24	80	1
8	60	24	96	1
9	80	24	93	0
10	100	24	38	3
11[a]	20	5	95	n.d.

[a] Reaction was performed using 2 equiv. of $Ca(NTf_2)_2$ and was monitored by ^{19}F NMR using 4-fluoro-*o*-xylene as internal standard; conversion is reported in place of yield.

Optimization of reaction concentration

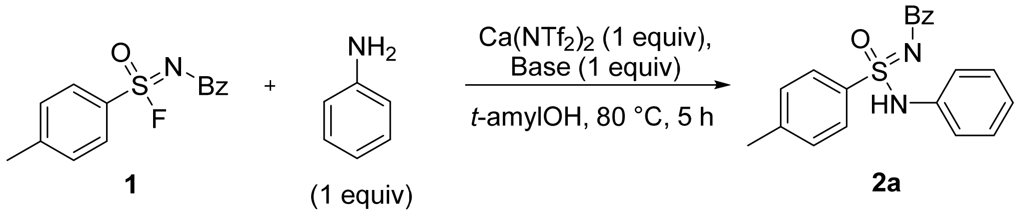

Table S5. The effects of concentrations of **1** and bases on the SuFEx reactions between sulfonimidoyl fluoride **1** and aniline.

Entry	Base	Conc. of 1 (M)	Yield (%)	Hydrolysis (%)
1	Aniline	0.23	>99	1
2[a]	Aniline	0.23	63	0
3	Aniline	0.11	94	0
4	Aniline	0.06	93	0
5	DABCO	0.23	64	8
6	DABCO	0.11	86	10
7	DABCO	0.06	79	14
8	DMA	0.23	62	4
9	DMA	0.11	70	5
10	DMA	0.06	58	5

[a] Reaction was performed using 0.3 equiv. of $Ca(NTf_2)_2$ and was monitored by ^1H NMR using 1,3-benzodioxole as internal standard.

Optimization of reaction time

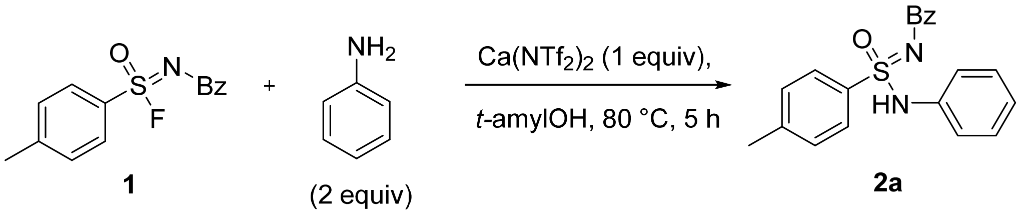

Table S6. The effects of reaction time on the SuFEx reactions between sulfonimidoyl fluoride **1** and aniline.

Entry	Reaction time (h)	Yield (%)	Hydrolysis (%)
1	1	87	0
2	2	88	0
3	3	91	0
4	4	94	0
5	5	>99	0

Discussion

Reaction optimization was performed using sulfonimidoyl fluoride **1** and aniline as starting materials and included variation of Lewis acids, bases, temperature, solvents and concentration. Because sulfonimidoyl fluorides can act as bidentate ligands, while sulfonyl fluorides are monodentate, we did not initially expect to arrive to reaction conditions similar to those reported by Ball.[3]

Initially, we thought that sulfonimidoyl fluorides would be better activated with softer Lewis acids due to the presence of sulfonimine bond.[4] However, we found that hard Lewis acids such as Ca^{2+}, Mg^{2+} and Ba^{2+} gave higher yield compared to softer acids Li^{+} and Zn^{2+} (Table 1). Furthermore, counter ion of the Lewis acid played an important role in this reaction as $Ca(NTf_2)_2$ gave higher conversion in comparison with $Ca(OTf)_2$. Interestingly, the choice of base was important in order to prevent the side hydrolysis of **1** (Table S2). The use of two equivalents of aniline as both base and nucleophile resulted in virtually quantitative yield in *t*-amylOH without the formation of hydrolysis side-products. The reaction performs well in protic solvents (*t*-amylOH, *t*-BuOH, *i*-PrOH), but does not work in polar aprotic solvents such as DMF and DMSO. Furthermore, we discovered that a complete conversion of starting material can be reached with 1 equiv of $Ca(NTf_2)_2$ at 80 °C in 0.23 M solution within 5 h. The reaction with 2 equivs of $Ca(NTf_2)_2$ reached 95% conversion at room temperature within 5 h.

N-SuFEx reactions of racemic 1 with aromatic amines

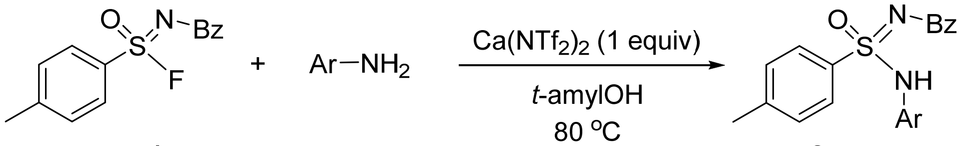

General procedure A:

The sulfonimidoyl fluoride **1** (0.25 mmol, 69 mg, 1 equiv), aromatic amine (0.5 mmol, 2 equiv), and calcium triflimide (0.25 mmol, 150 mg, 1 equiv) were charged into an oven-dried 5 mL microwave vial. 1.25 mL of *tert*-amyl alcohol was injected and the reaction mixture was heated to 80 °C. After 5 or 24 h, the reaction mixture was cooled to room temperature and diluted with 10 mL ethyl acetate. This mixture was washed with 20 mL saturated NaCl, and the residue aqueous phase was extracted by ethyl acetate (2 x 10 mL). All the organic phases were combined and dried with Na_2SO_4. After the concentration of the organic solution, the residue mixture was further purified via flash column chromatography to give the target product.

General procedure B:

The sulfonimidoyl fluoride **1** (0.5 mmol, 139 mg, 1 equiv), aromatic amine (1.5 mmol, 3 equiv), and calcium triflimide (0.5 mmol, 300 mg, 1 equiv) were charged into an oven-dried 10 mL vial. 3 mL of *tert*-amyl alcohol was injected and the reaction mixture was heated to 80 °C. After 2 h, the reaction mixture was cooled to room temperature and diluted with 20 mL ethyl acetate. This mixture was washed by 50 mL saturated NH_4Cl, and the residue aqueous phase was extracted by ethyl acetate (2 × 20 mL). All the organic phases were combined and dried with Na_2SO_4. After the concentration of the organic solution, the residue mixture was further purified via flash column chromatography to give the target product.

Experimental data

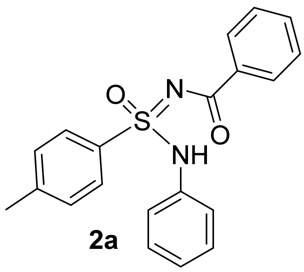

2a

Rac-2a was synthesized according to the general procedure A with a reaction time of 5 h. Purification was performed with flash column chromatography on silica gel (from 60:40 to 90:10 DCM/*n*-hex) providing the title compound as white solid (84 mg, 0.24 mmol; 96% yield). ^{1}H NMR (400 MHz, $CDCl_3$) *δ* 10.39 (s, 1H), 8.32 – 8.07 (m, 2H), 7.92 – 7.73 (m, 2H), 7.56 – 7.48 (m, 1H), 7.46 – 7.37 (m, 2H), 7.28 – 7.19 (m, 4H), 7.18 – 7.12 (m, 2H), 7.12 – 7.06 (m, 1H), 2.35 (s, 3H). ^{13}C NMR (101 MHz, $CDCl_3$) *δ* 173.1, 144.7, 136.4, 136.0, 135.6, 132.8, 130.1, 129.8, 129.6, 128.3, 127.4, 125.7, 122.4, 21.8. IR (neat): 3065, 1598,1 572, 1276, 712, 522 cm^{-1}. HRMS (ESI, m/z): calculated for $C_{20}H_{18}N_2O_2S$ [M+H]$^+$: 351.1173, found: 351.1160.

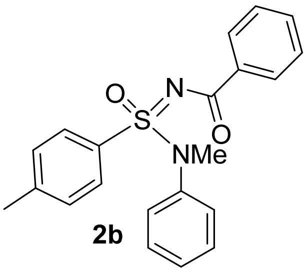

2b

Rac-2b was synthesized according to the general procedure A with a reaction time of 5 h. Purification was performed with flash column chromatography on silica gel (from 10:90 to 20:10 EtOAc/*n*-hex) providing the title compound as white solid (57 mg, 0.16 mmol; 91% yield). ^{1}H NMR (400 MHz, $CDCl_3$) *δ* 7.99 (d, *J* = 7.6 Hz, 2H), 7.62 (d, *J* = 7.9 Hz, 2H), 7.38 (t, 1H), 7.28 (t, *J* = 7.6 Hz, 2H), 7.23 – 7.16 (m, 5H), 7.14 – 7.07 (m, 2H), 3.19 (s, 3H), 2.33 (s, 3H). ^{13}C NMR (101 MHz, $CDCl_3$) δ 172.4, 143.9, 141.0, 135.9, 133.8, 131.8, 129.5, 129.3, 128.8, 127.9, 128.0 (confirmed by HSQC), 127.8, 127.7, 38.5, 21.4. IR (neat): 3062, 1634, 1595, 1246, 1137 cm^{-1}. HRMS (ESI, m/z): calculated for $C_{21}H_{20}N_2O_2S$ [M+Na]$^+$: 387.1138, found: 387.1136.

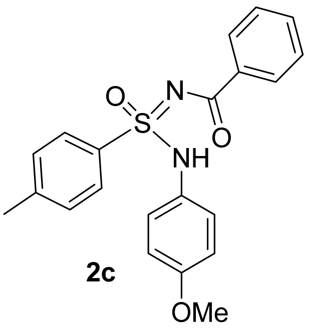

2c

Rac-2c was synthesized according to the general procedure A with a reaction time of 5 h. Heating was performed for 5 h. Purification was performed with flash column chromatography on silica gel (from 10:90 to 60:40 EtOAc/*n*-hex) providing the title compound as orange oil (85 mg, 0.22 mmol; 89% yield). ^{1}H NMR (400 MHz, $CDCl_3$) δ 9.91 (s, 1H), 8.25 – 8.14 (m, 2H), 7.76 (d, J = 8.4 Hz, 2H), 7.58 – 7.47 (m, 1H), 7.47 – 7.38 (m, 2H), 7.23 (d, J = 8.0 Hz, 2H), 7.12 – 7.01 (m, 2H), 6.82 – 6.70 (m, 2H), 3.74 (s, 3H), 2.37 (s, 3H). ^{13}C NMR (101 MHz, $CDCl_3$) δ 173.2, 158.2, 144.5, 136.2, 135.6, 132.6, 129.9, 129.8, 128.2, 127.9, 127.4, 126.0, 114.7, 55.5, 21.7. IR (neat): 3065, 1602, 1572, 1508, 1316, 1245, 1143, 1026, 807 cm^{-1} HRMS (ESI, m/z): calculated for $C_{21}H_{20}N_2O_3S$ $[M+H]^+$: 381.1278, found: 381.1268.

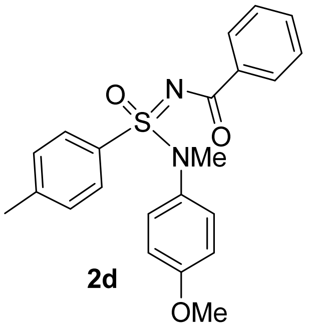

2d

Rac-2d was synthesized according to the general procedure A with a reaction time of 24 h. Purification was performed with flash column chromatography on silica gel (from 10:90 to 60:40 EtOAc/*n*-hex) providing the title compound as colorless oil (89 mg, 0.22 mmol; 91% yield). ^{1}H NMR (400 MHz, $CDCl_3$) δ 8.15 – 8.08 (m, 2H), 7.72 (d, J = 8.4 Hz, 2H), 7.54 – 7.44 (m, 1H), 7.39 (t, J = 7.6 Hz, 2H), 7.30 (d, J = 8.4 Hz, 2H), 7.14 – 7.05 (m, 2H), 6.85 – 6.75 (m, 2H), 3.78 (s, 3H), 3.28 (s, 3H), 2.44 (s, 3H). ^{13}C NMR (101 MHz, $CDCl_3$) δ 172.8, 159.2, 144.1, 136.3, 134.2, 133.7, 132.1, 129.8, 129.7, 129.6, 128.2, 128.1, 114.3, 55.6, 39.2, 21.7. IR (neat): 3064, 1636, 1508, 1279, 1245, 1141, 714 cm^{-1}. HRMS (ESI, m/z): calculated for $C_{22}H_{22}N_2O_3S$ $[M+H]^+$: 395.1435, found: 395.1411.

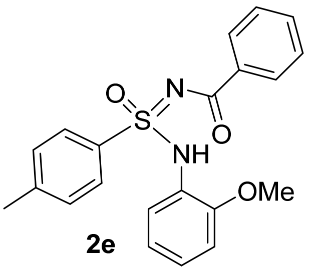

2e

Rac-2e was synthesized according to the general procedure A with a reaction time of 24 h. Purification was performed with flash column chromatography on silica gel (from 40:40:5 to 40:40:10 DCM/*n*-hex/EtOAc) providing the title compound as colorless oil (66 mg, 0.17 mmol; 70% yield). ^{1}H NMR (400 MHz, $CDCl_3$) δ 10.25 (s, 1H), 8.30 – 8.15 (m, 2H), 7.83 (d, J = 8.4 Hz, 2H), 7.53 – 7.47 (m, 1H), 7.46 – 7.37 (m, 3H), 7.19 (d, J = 8.2 Hz, 2H), 7.08 – 7.00 (m, 1H), 6.87 – 6.78 (m, 2H), 3.86 (s, 3H), 2.33 (s, 3H). ^{13}C NMR (101 MHz, $CDCl_3$) δ 172.6, 150.1, 144.4, 136.5, 135.6, 132.5, 129.7 (2C confirmed by HSQC), 128.1, 127.3, 125.7, 125.5, 121.8, 121.0, 111.0, 56.0, 21.6. IR (neat): 3065, 1614, 1597, 1498, 1293, 1251, 713 cm^{-1}. HRMS (ESI, m/z): calculated for $C_{21}H_{20}N_2O_3S$ $[M+H]^+$: 381.1267, found: 381.1262.

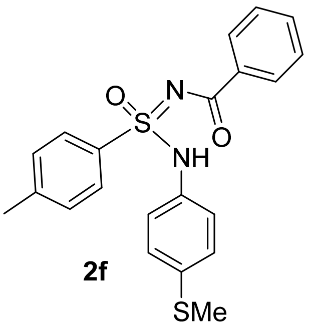

2f

Rac-2f was synthesized according to the general procedure A with a reaction time of 24 h. Purification was performed with flash column chromatography on silica gel (40:40:5 to 40:40:10 DCM/*n*-hex/EtOAc) providing the title compound as white solid (83 mg, 0.21 mmol; 83% yield). ^{1}H NMR (400 MHz, $CDCl_3$) δ 10.31 (s, 1H), 8.19 (d, J = 7.2 Hz, 2H), 7.79 (d, J = 8.1 Hz, 2H), 7.52 (t, J = 7.3 Hz, 1H), 7.42 (t, J = 7.6 Hz, 2H), 7.24 (d, J = 8.0 Hz, 2H), 7.12 (d, J = 8.7 Hz, 2H), 7.07 (d, J = 8.8 Hz, 2H), 2.42 (s, 3H), 2.37 (s, 3H). ^{13}C NMR (101 MHz, $CDCl_3$) δ 173.0, 144.7, 136.1, 136.0, 135.4, 132.9, 132.7, 130.0, 129.7, 128.2, 127.7, 127.3, 123.4, 21.7, 16.1. IR (neat): 3065, 1599, 1572, 1492, 1289, 1144, 713 cm^{-1}. HRMS (ESI, m/z): calculated for $C_{21}H_{20}N_2O_2S_2$ $[M+Na]^+$: 419.0858, found: 419.0863.

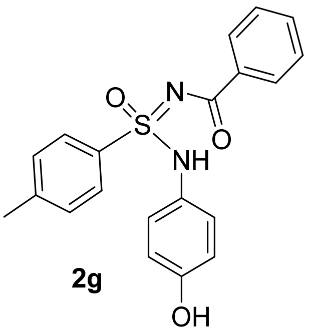

2g

Rac-2g was synthesized according to the general procedure B with a reaction time of 2 h. Purification was performed with flash column chromatography on silica gel (from 10:90 to 60:40 EtOAc/*n*-hex). After column, product was further recrystallized with EtOAc/*n*-hex providing the title compound as brown powder (63 mg, 0.17 mmol; 37% yield). ^{1}H NMR (400 MHz, DMSO) δ 10.03 (s, 1H), 9.39 (s, 1H), 8.06 – 7.98 (m, 2H), 7.67 (d, J = 8.3 Hz, 2H), 7.55 (t, J = 7.3 Hz, 1H), 7.46 (t, J = 7.5 Hz, 2H), 7.35 (d, J = 8.1 Hz, 2H), 6.88 (d, J = 8.8 Hz, 2H), 6.61 (d, J = 8.9 Hz, 2H), 2.34 (s, 3H). ^{13}C NMR (101 MHz, DMSO) δ 171.3, 155.5, 143.3, 136.0, 135.6, 132.0, 129.6, 128.8, 128.1, 127.6, 126.5, 125.7, 115.6, 21.0. IR (neat): 3160, 1569, 1566, 1510, 1330, 712 cm^{-1}. HRMS (ESI, m/z): calculated for $C_{20}H_{19}N_2O_3S$ [M+H]$^+$: 367.1111, found: 367.1101.

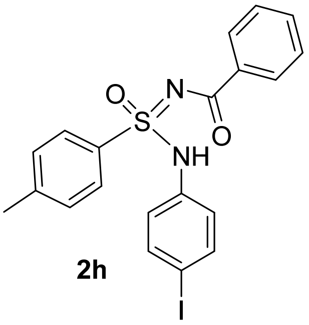

2h

Rac-2h was synthesized according to the general procedure B with a reaction time of 5 h. Purification was performed with flash column chromatography on silica gel (10:90 to 60:40 EtOAc/*n*-hex) providing the title compound as white solid (87 mg, 0.18 mmol; 73% yield). ^{1}H NMR (400 MHz, $CDCl_3$) δ 10.41 (s, 1H), 8.17 (d, J = 7.7 Hz, 2H), 7.80 (d, J = 8.3 Hz, 2H), 7.56 – 7.47 (m, 3H), 7.40 (t, J = 7.6 Hz, 2H), 7.24 (d, J = 8.1 Hz, 2H), 6.91 (d, J = 8.7 Hz, 2H), 2.36 (s, 3H). ^{13}C NMR (101 MHz, $CDCl_3$) δ 173.0, 144.9, 138.5, 135.9, 135.2, 132.8, 130.1, 129.7, 128.2, 127.3, 123.8, 122.2, 89.5, 21.7. IR (neat): 3065, 1601, 1572, 1485, 1292, 1146, 712 cm^{-1}. HRMS (ESI, m/z): calculated for $C_{20}H_{17}N_2O_2SI$ [M+H]$^+$: 477.0139, found: 477.0130.

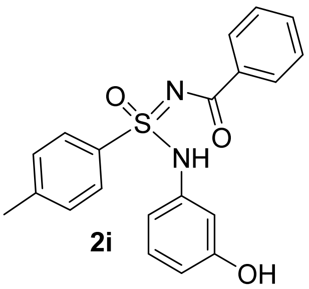

2i

Rac-2i was synthesized according to the general procedure B with a reaction time of 2 h. Purification was performed with flash column chromatography on silica gel (from 10:90 to 60:40 EtOAc/*n*-hex) providing the title compound as white powder (162 mg, 0.45 mmol; 89% yield). ^{1}H NMR (400 MHz, DMSO) δ 10.47 (s, 1H), 9.49 (s, 1H), 8.11 – 7.97 (m, 2H), 7.78 (d, J = 8.3 Hz, 2H), 7.56 (t, J = 7.4 Hz, 1H), 7.46 (t, J = 7.6 Hz, 2H), 7.38 (d, J = 8.1 Hz, 2H), 6.99 (t, J = 8.1 Hz, 1H), 6.61 (t, J = 2.2 Hz, 1H), 6.55 (dd, J = 8.4, 1.7 Hz, 1H), 6.42 (dd, J = 8.1, 2.4 Hz, 1H), 2.34 (s, 3H). ^{13}C NMR (101 MHz, DMSO) δ 171.2, 157.8, 143.6, 137.7, 135.8, 135.6, 132.1, 129.8, 129.7, 128.9, 128.2, 127.7, 111.5, 111.4, 107.8, 21.0. IR (neat): 3237, 1596, 1569, 1488, 1284, 1139 cm^{-1}. HRMS (ESI, m/z): calculated for $C_{20}H_{18}N_2O_3SNa$ [M+Na]$^+$: 389.0930, found: 389.0919.

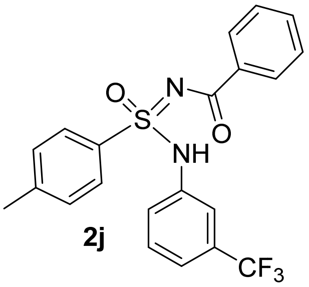

2j

Rac-2j was synthesized according to the general procedure B with a reaction time of 2 h. Purification was performed with flash column chromatography on silica gel (from 20:80 to 70:30 EtOAc/*n*-hex) providing the title compound as white powder (188 mg, 0.45 mmol; 90% yield). ^{1}H NMR (400 MHz, DMSO) δ 11.02 (s, 1H), 8.08 – 7.98 (m, 2H), 7.82 (d, J = 8.4 Hz, 2H), 7.62 – 7.53 (m, 1H), 7.53 – 7.42 (m, 4H), 7.44 – 7.35 (m, 4H), 2.35 (s, 3H). ^{13}C NMR (101 MHz, DMSO-d_6) δ 171.2, 144.1, 137.9, 135.5, 135.2, 132.3, 130.6, 130.0, 129.8 (q, J = 31.6 Hz), 128.8, 128.3, 127.5, 124.1, 123.7 (q, J = 270.8 Hz), 120.7 (q, J = 3.9 Hz), 116.7 (q, J = 3.8 Hz), 21.0. ^{19}F NMR (377 MHz, $CDCl_3$) δ -62.95. IR (neat): 3069, 1596, 1568, 1318, 1254, 1125 cm^{-1}. HRMS (ESI, m/z): calculated for $C_{21}H_{18}N_2F_3O_2S$ [M+H]$^+$: 419.1036, found: 419.1023.

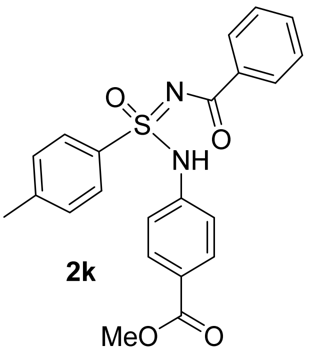

2k

Rac-2k was synthesized according to the general procedure B with a reaction time of 2 h. Purification was performed with flash column chromatography on silica gel (from 20:80 to 80:20 EtOAc/*n*-hex) providing the title compound as white powder (118 mg, 0.29 mmol; 58% yield). ^{1}H NMR (400 MHz, DMSO) δ 11.17 (s, 1H), 8.08 – 8.01 (m, 2H), 7.83 (dd, J = 14.4, 8.6 Hz, 4H), 7.61 – 7.54 (m, 1H), 7.47 (t, J = 7.5 Hz, 2H), 7.40 (d, J = 8.1 Hz, 2H), 7.25 (d, J = 8.7 Hz, 2H), 3.77 (s, 3H), 2.34 (s, 3H). ^{13}C NMR (101 MHz, DMSO) δ 171.1, 165.6, 144.1, 141.9, 135.5, 135.4, 132.3, 130.5, 129.9, 128.9, 128.2, 127.6, 124.5, 118.9, 51.9, 21.0. IR (neat): 3064, 1717, 1601, 1273, 951 cm^{-1}. HRMS (ESI, m/z): calculated for $C_{22}H_{21}N_2O_4S$ [M+H]$^+$: 409.1217, found: 409.1207.

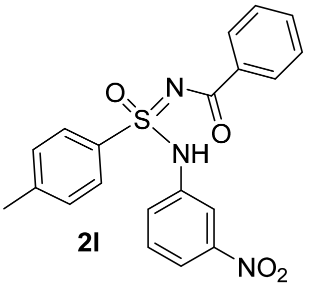

2l

Rac-2l was synthesized according to the general procedure A with a reaction time of 24 h. Purification was performed with flash column chromatography on silica gel (from 10:90 to 60:40 EtOAc/*n*-hex) providing the title compound as white solid (53 mg, 0.13 mmol; 54% yield). ^{1}H NMR (400 MHz, $CDCl_3$) δ 11.08 (s, 1H), 8.18 (d, J = 7.2 Hz, 2H), 7.99 (s, 1H), 7.93 (d, J = 8.0 Hz, 1H), 7.85 (d, J = 8.1 Hz, 2H), 7.59 – 7.47 (m, 2H), 7.47 – 7.38 (m, 3H), 7.29 (d, J = 8.1 Hz, 2H), 2.39 (s, 3H). ^{13}C NMR (101 MHz, $CDCl_3$) δ 173.1, 149.0, 145.5, 137.7, 135.8, 135.0, 133.1, 130.5, 130.4, 129.8, 128.4, 127.4, 127.0, 119.9, 116.1, 21.8. IR (neat): 3100, 1601, 1573, 1530, 1279, 713 cm^{-1}. HRMS (ESI, m/z): calculated for $C_{20}H_{17}N_3O_4S$ [M-H]$^-$: 394.0862, found: 394.0856.

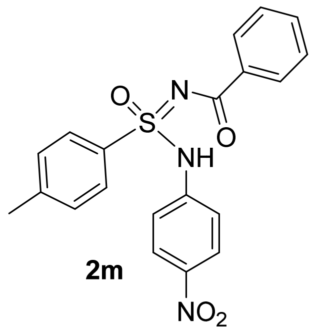

We aimed to synthesize **Rac-2m** according to the general procedure B with a reaction time of 24 h. No product formation was observed by TLC. Only traces of product were found with HRMS (below).

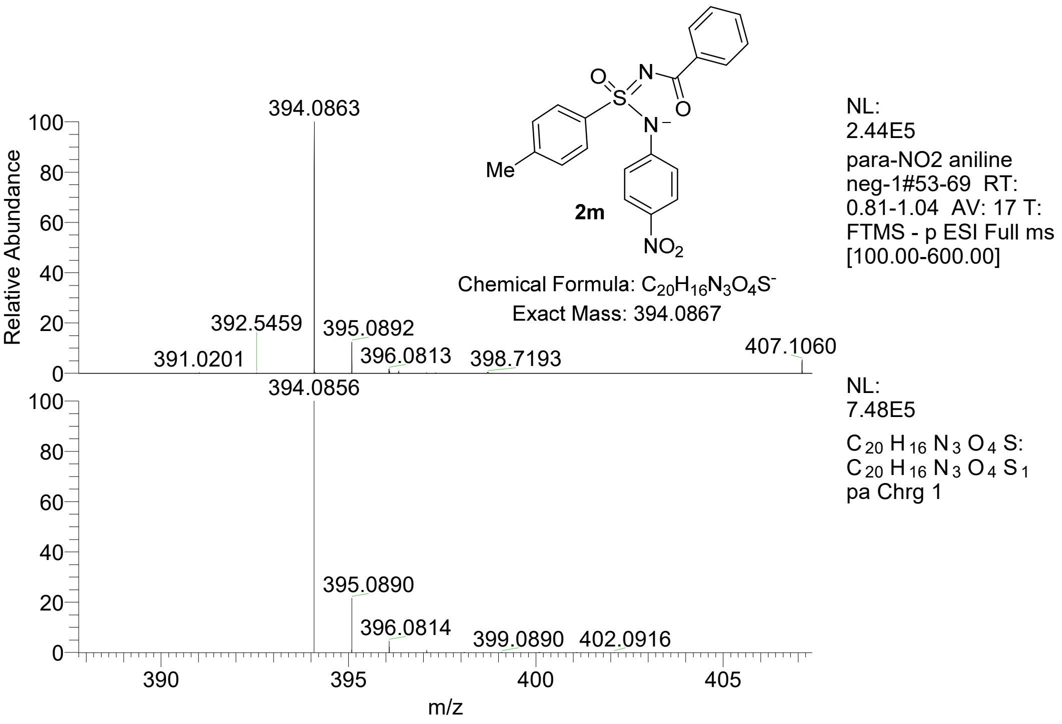

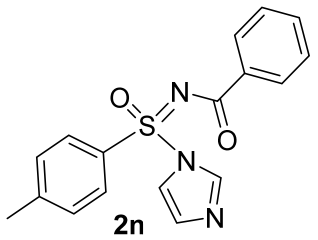

Rac-2n was synthesized according to the general procedure B with a reaction time of 2 h. Purification was performed with flash column chromatography on silica gel (from 20:80 to 70:30 EtOAc/*n*-hex) providing the title compound as white powder (135 mg, 0.42 mmol; 83% yield). ^{1}H NMR (400 MHz, DMSO) δ 8.42 (s, 1H), 8.15 (d, J = 8.3 Hz, 4H), 7.74 (t, J = 1.6 Hz, 1H), 7.72 – 7.63 (m, 1H), 7.61 – 7.49 (m, 4H), 7.13 (s, 1H), 2.43 (s, 3H). ^{13}C NMR (101 MHz, DMSO) δ 171.2, 146.6, 137.3, 134.1 (2 peaks), 133.3, 131.3, 130.7, 129.5, 128.6, 127.3, 118.3, 21.1. IR (neat): 1653, 1452, 1451, 1254, 1144, 1042 cm^{-1}. HRMS (ESI, m/z): calculated for $C_{17}H_{16}N_3O_2S$ [M+H]$^+$: 348.0777, found: 348.0769.

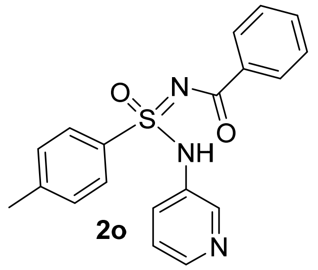

Rac-2o was synthesized according to the general procedure A with a reaction time of 5 h. Purification was performed with flash column chromatography on silica gel (from 100:2.5 to 100:5 DCM/MeOH) providing the title compound as off-white solid (74 mg, 0.21 mmol; 84% yield). ^{1}H NMR (400 MHz, DMSO-d_6) δ 11.06 (s, 1H), 8.33 (s, 1H), 8.25 (d, J = 4.9 Hz, 1H), 8.03 (d, J = 7.1 Hz, 2H), 7.80 (d, J = 8.3 Hz, 2H), 7.61 – 7.50 (m, 2H), 7.46 (t, J = 7.6 Hz, 2H), 7.39 (d, J = 8.1 Hz, 2H), 7.30 (dd, J = 8.3, 4.7 Hz, 1H), 2.34 (s, 3H). ^{13}C NMR (101 MHz, DMSO) δ 171.2, 145.0, 143.9, 142.3, 135.6, 135.5, 134.3 (confirmed by HMBC), 132.2, 129.9, 128.8 (2C confirmed by HSQC), 128.2, 127.5, 124.1, 21.0. IR (neat): 1631, 1578, 1264, 1249, 954, 722 cm^{-1}. HRMS (ESI, m/z): calculated for $C_{19}H_{17}N_3O_2S$ [M+H]$^+$: 352.1114, found: 352.1118.

NMR spectra

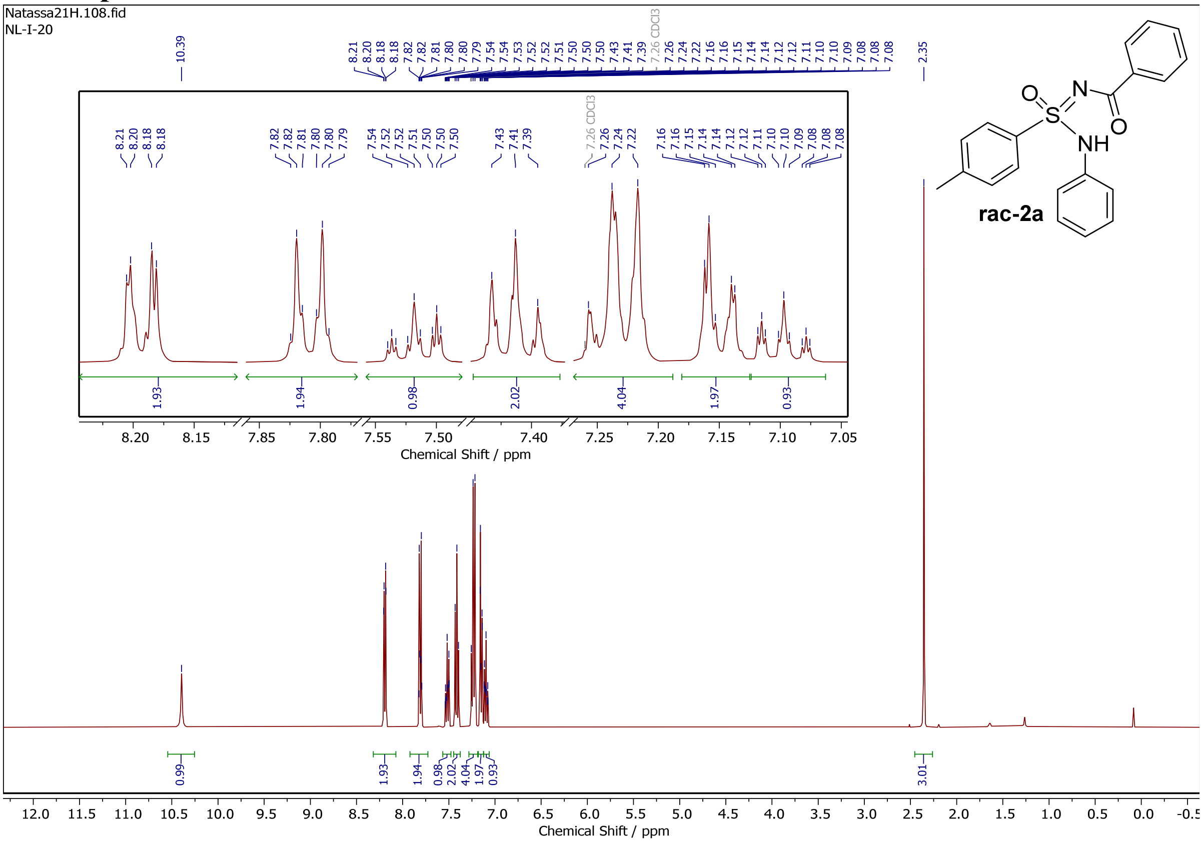

Figure S2. ^{1}H NMR (400 MHz) spectra of compound **rac-2a** ($CDCl_3$, 298 K).

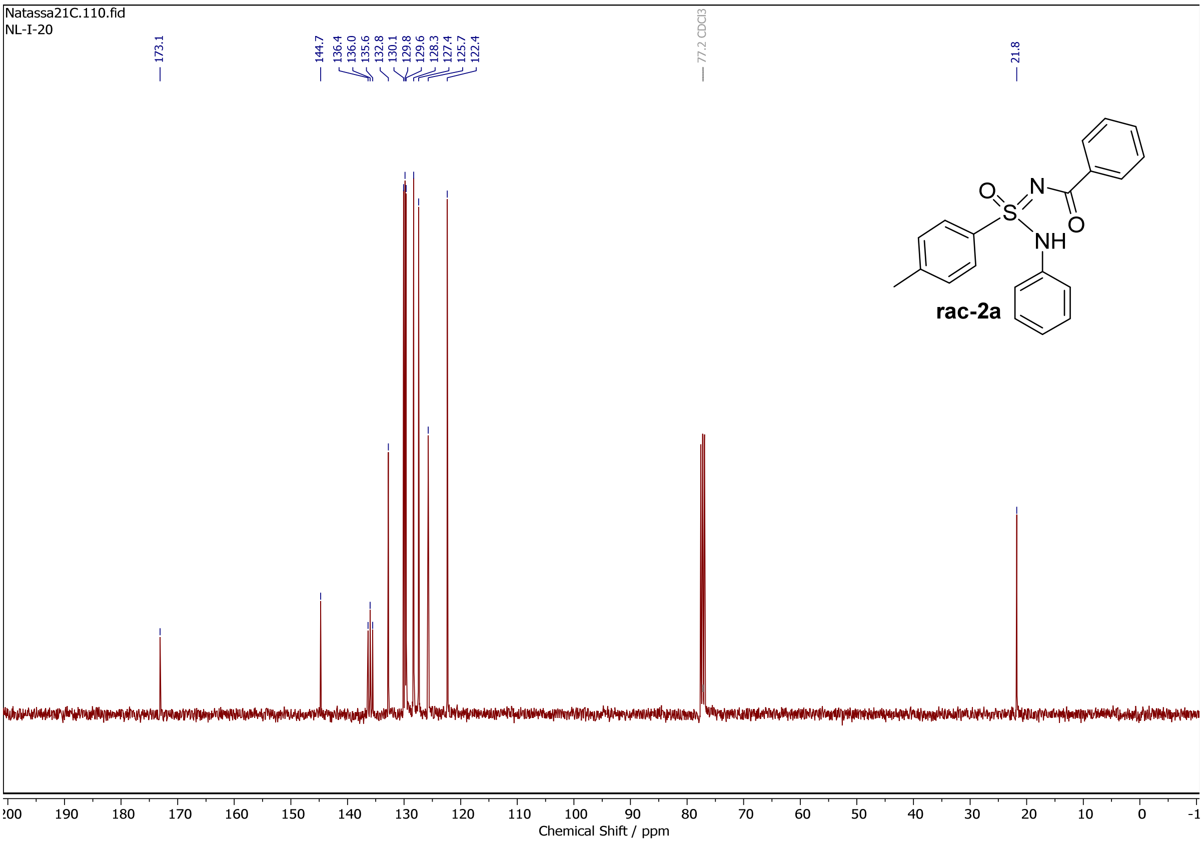

Figure S3. ^{13}C NMR (101 MHz) spectra of compound **rac-2a** ($CDCl_3$, 298 K).

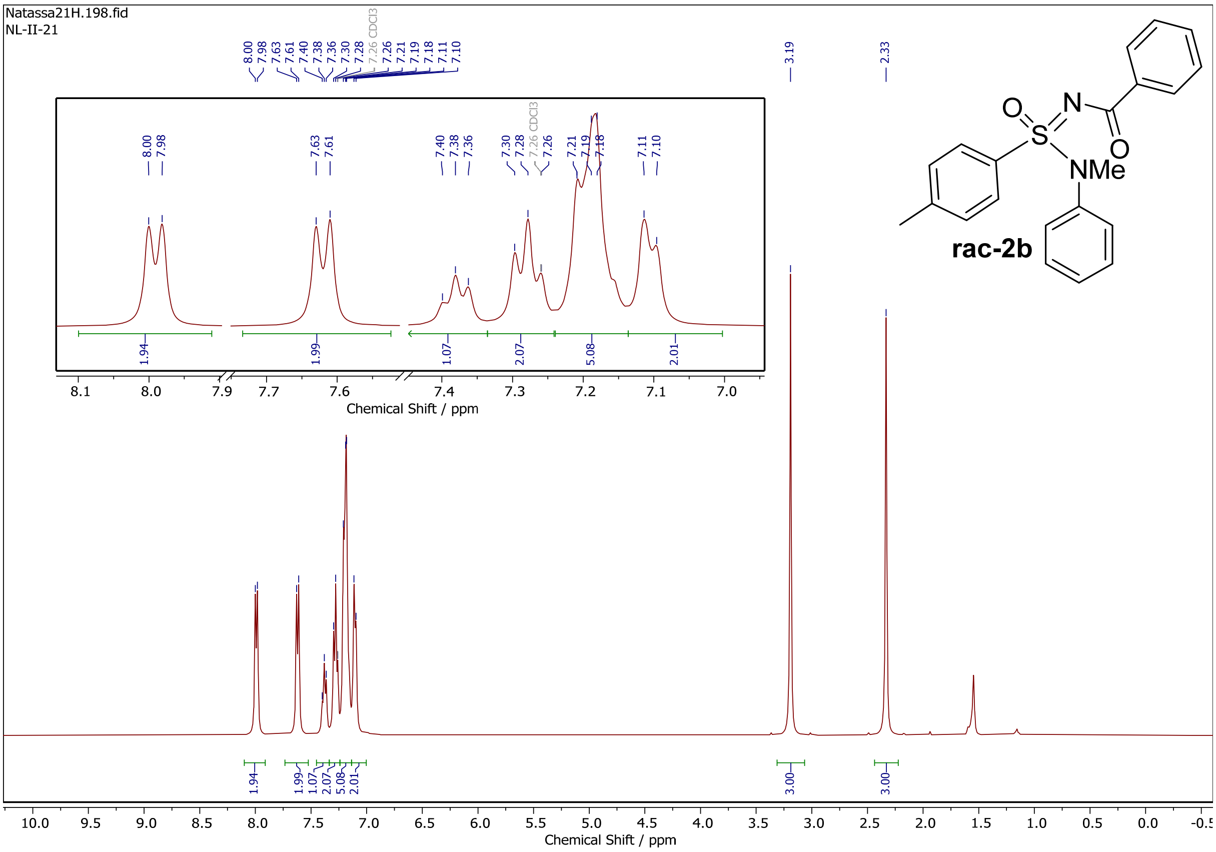

Figure S4. ^{1}H NMR (400 MHz) spectra of compound **rac-2b** ($CDCl_3$, 298 K).

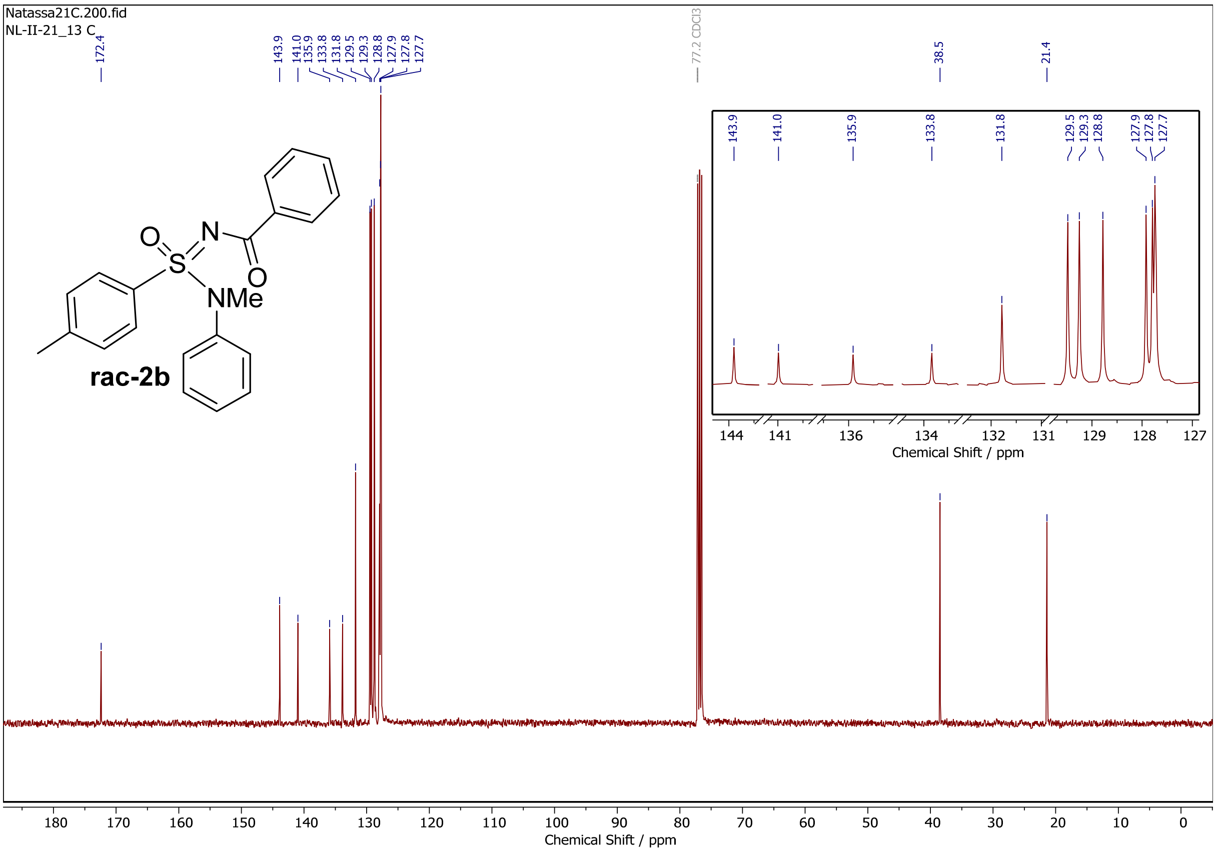

Figure S5. ^{13}C NMR (101 MHz) spectra of compound **rac-2b** ($CDCl_3$, 298 K).

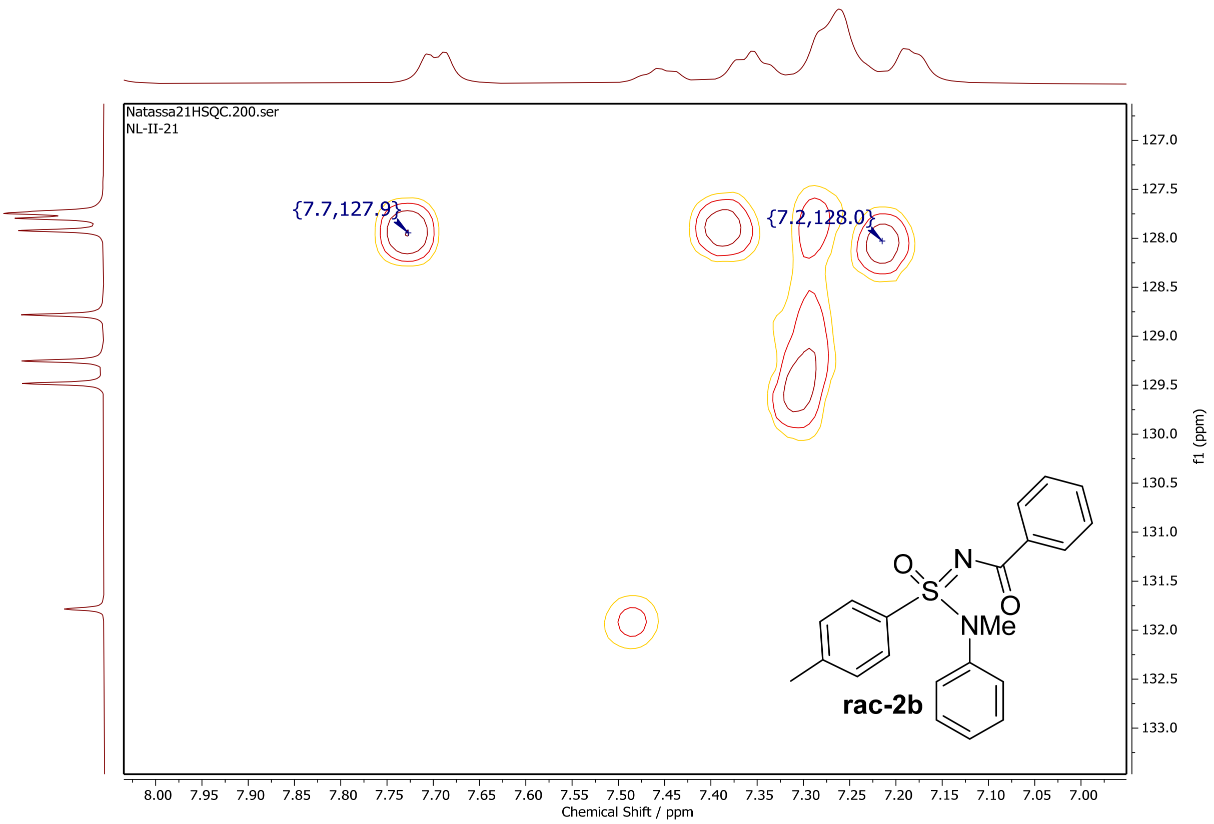

Figure S6. HSQC spectra of compound **rac-2b** ($CDCl_3$, 298 K).

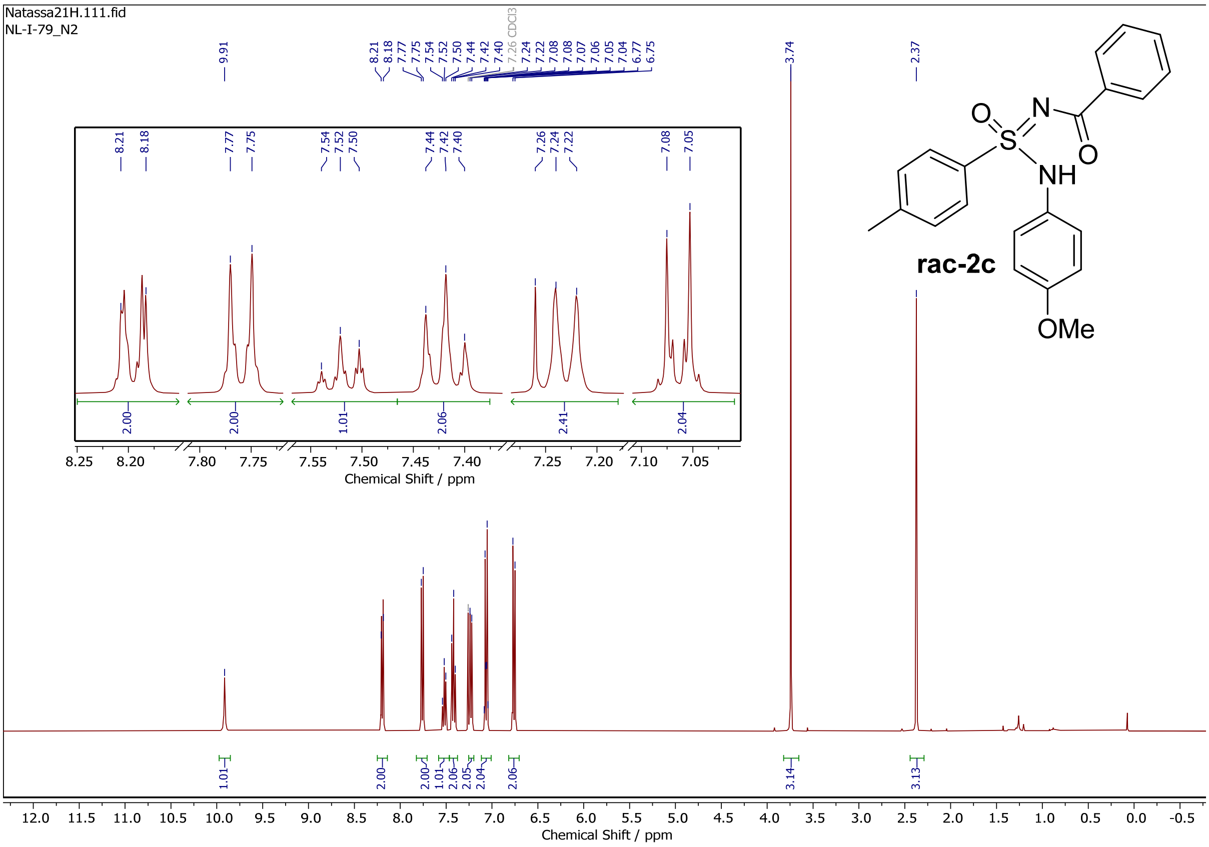

Figure S7. ^{1}H NMR (400 MHz) spectra of compound **rac-2c** ($CDCl_3$, 298 K).

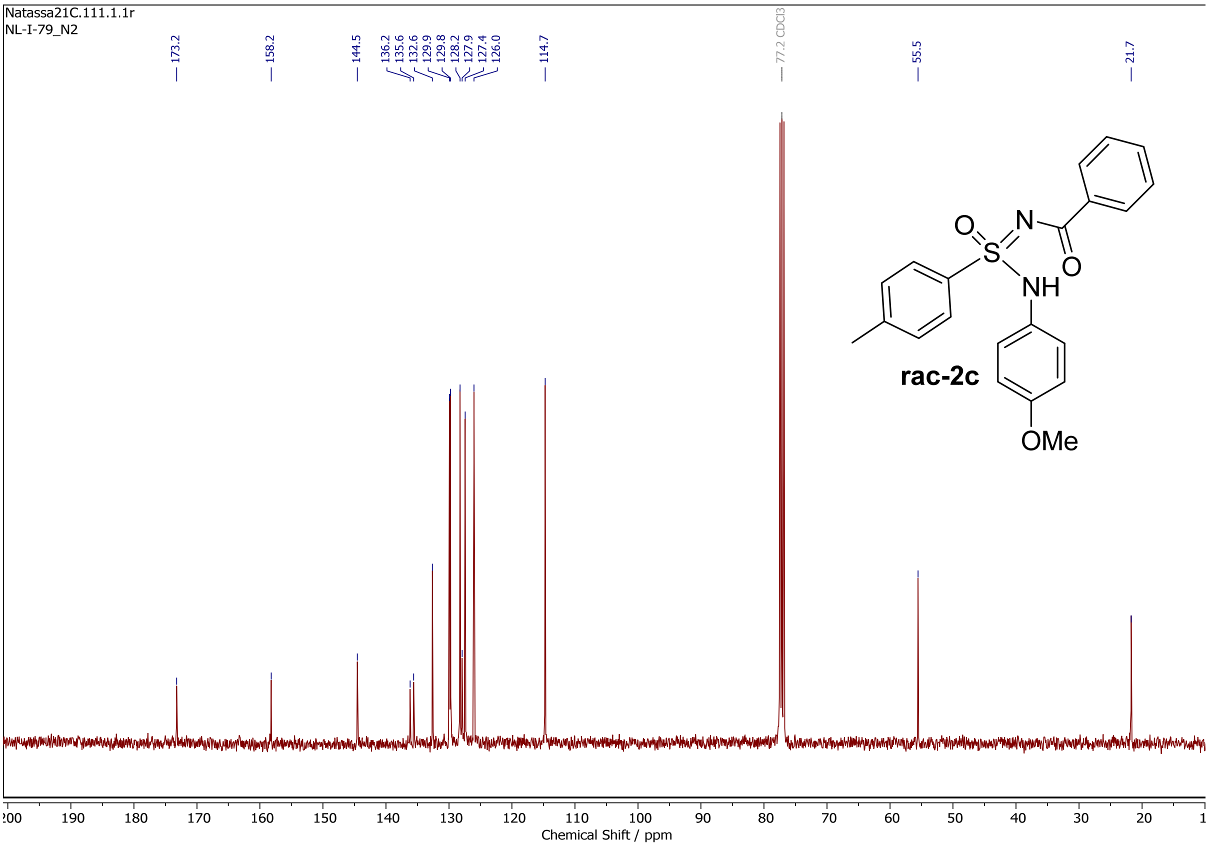

Figure S8. ^{13}C NMR (101 MHz) spectra of compound **rac-2c** ($CDCl_3$, 298 K).

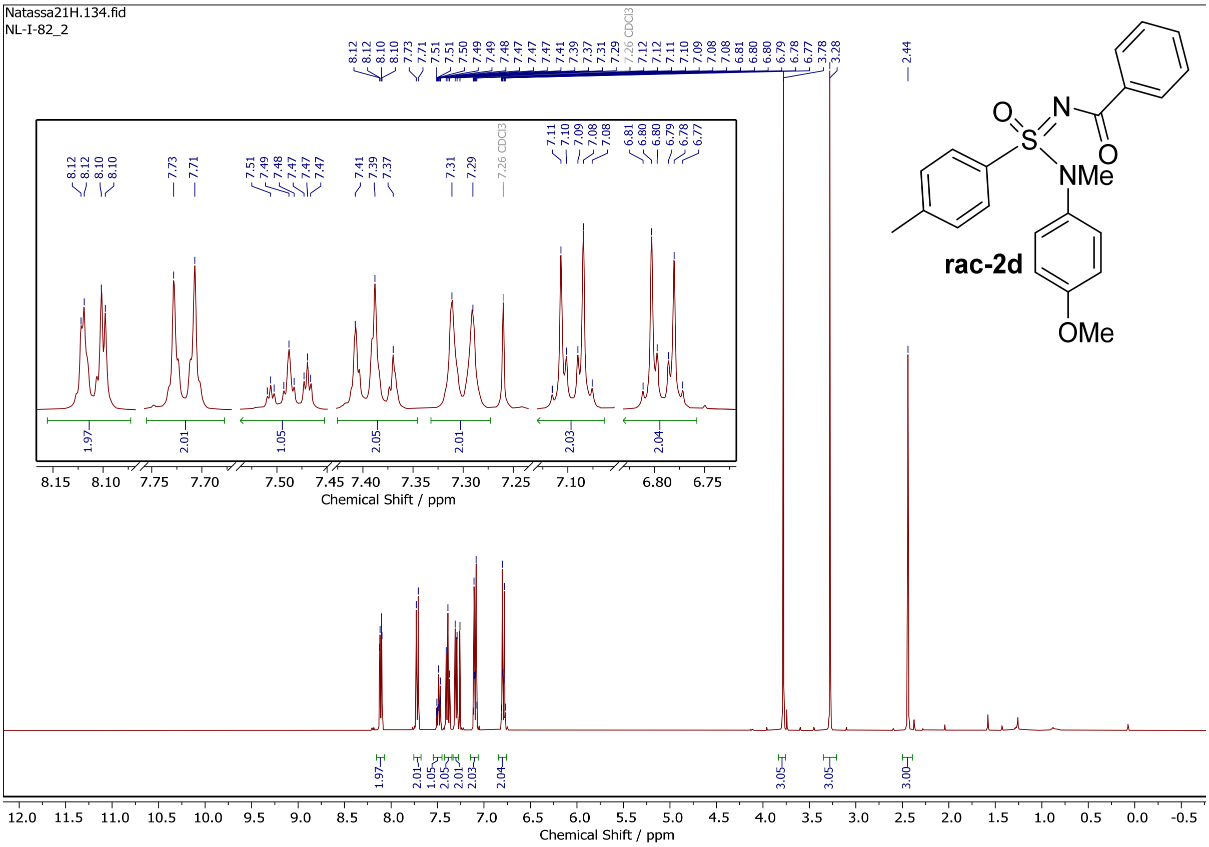

Figure S9. ^{1}H NMR (400 MHz) spectra of compound **rac-2d** ($CDCl_3$, 298 K).

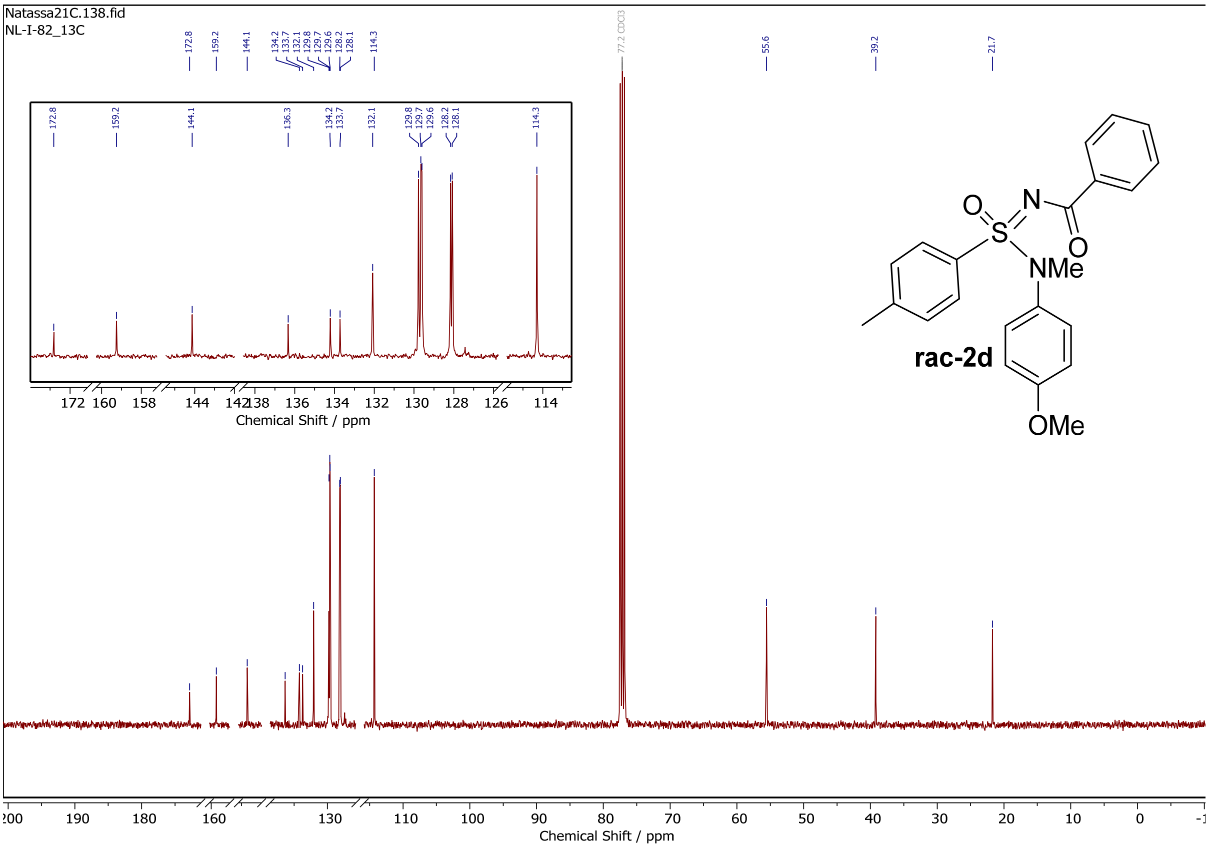

Figure S10. ^{13}C NMR (101 MHz) spectra of compound **rac-2d** ($CDCl_3$, 298 K).

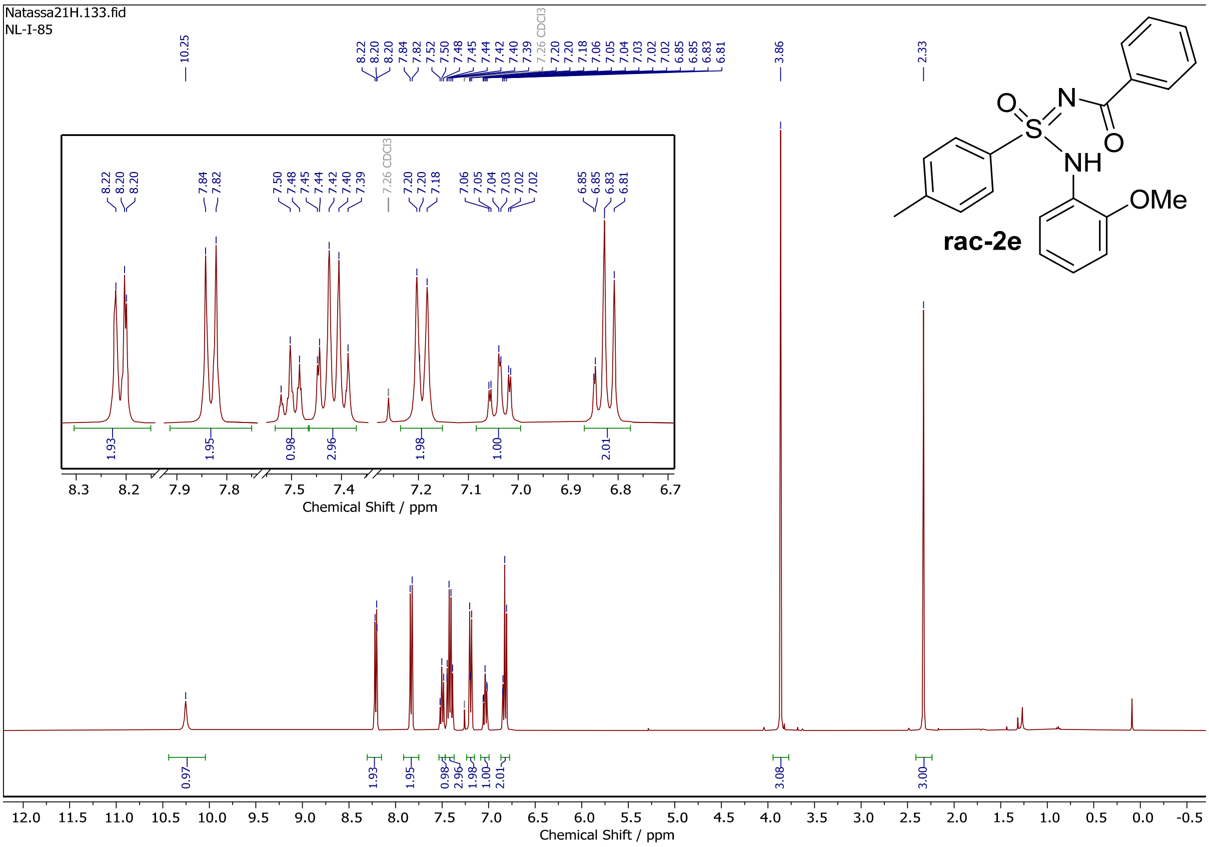

Figure S11. ^{1}H NMR (400 MHz) spectra of compound **rac-2e** ($CDCl_3$, 298 K).

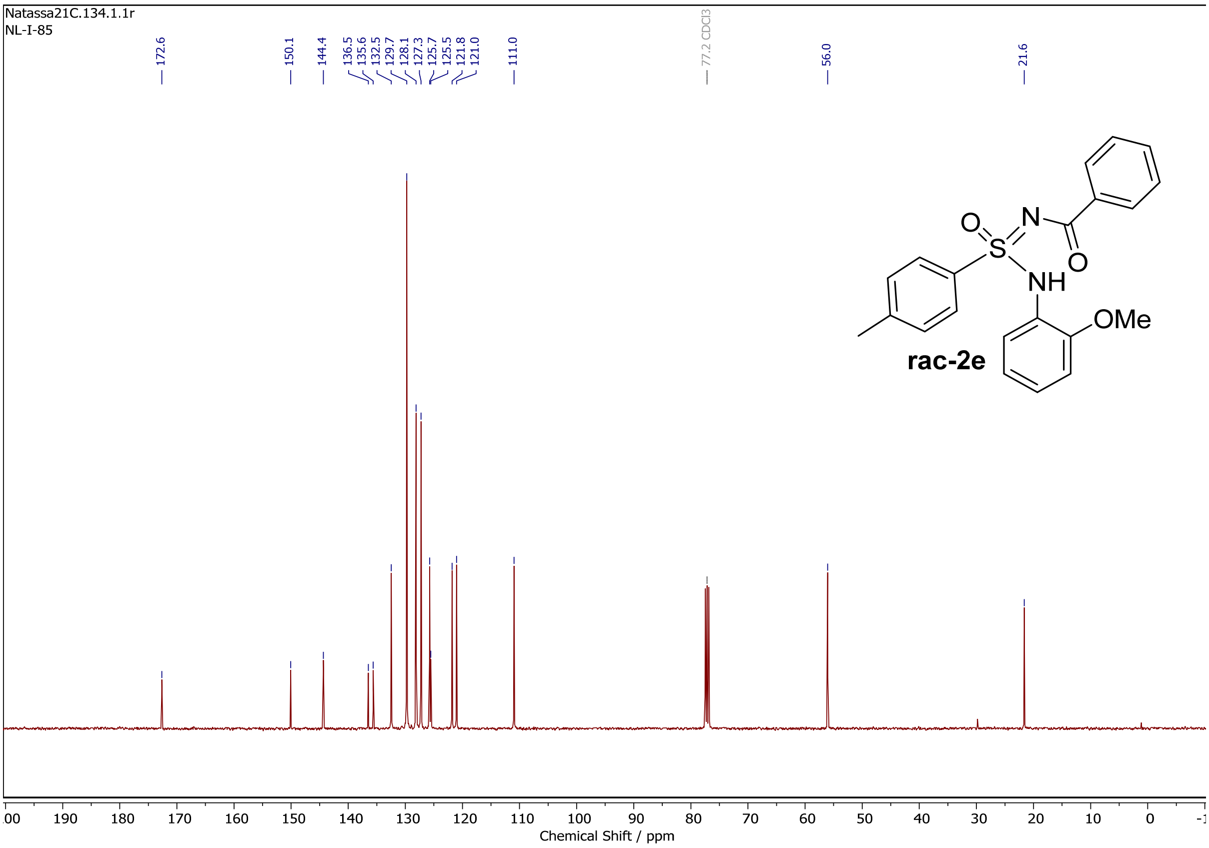

Figure S12. ^{13}C NMR (101 MHz) spectra of compound **rac-2e** ($CDCl_3$, 298 K).

HSQC Spectrum 2e

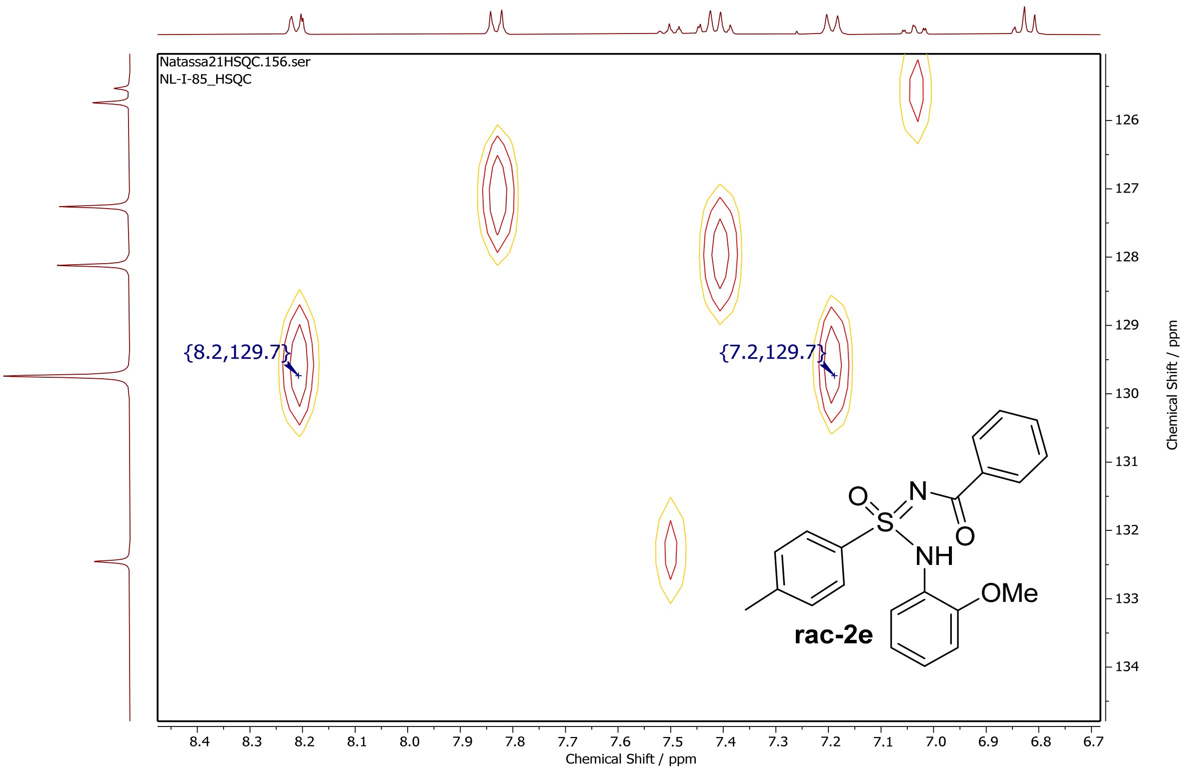

Figure S13. HSQC spectra of compound **rac-2e** ($CDCl_3$, 298 K).

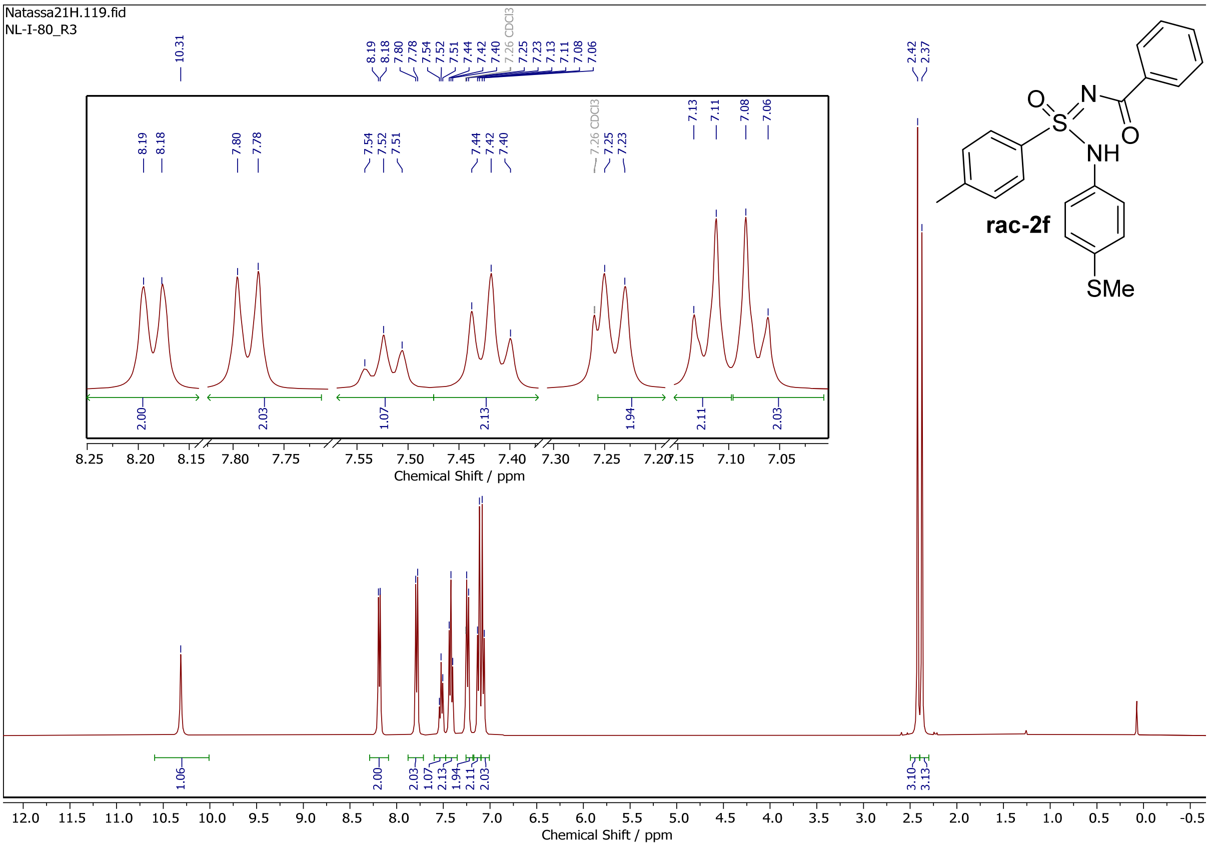

Figure S14. ^{1}H NMR (400 MHz) spectra of compound **rac-2f** ($CDCl_3$, 298 K).

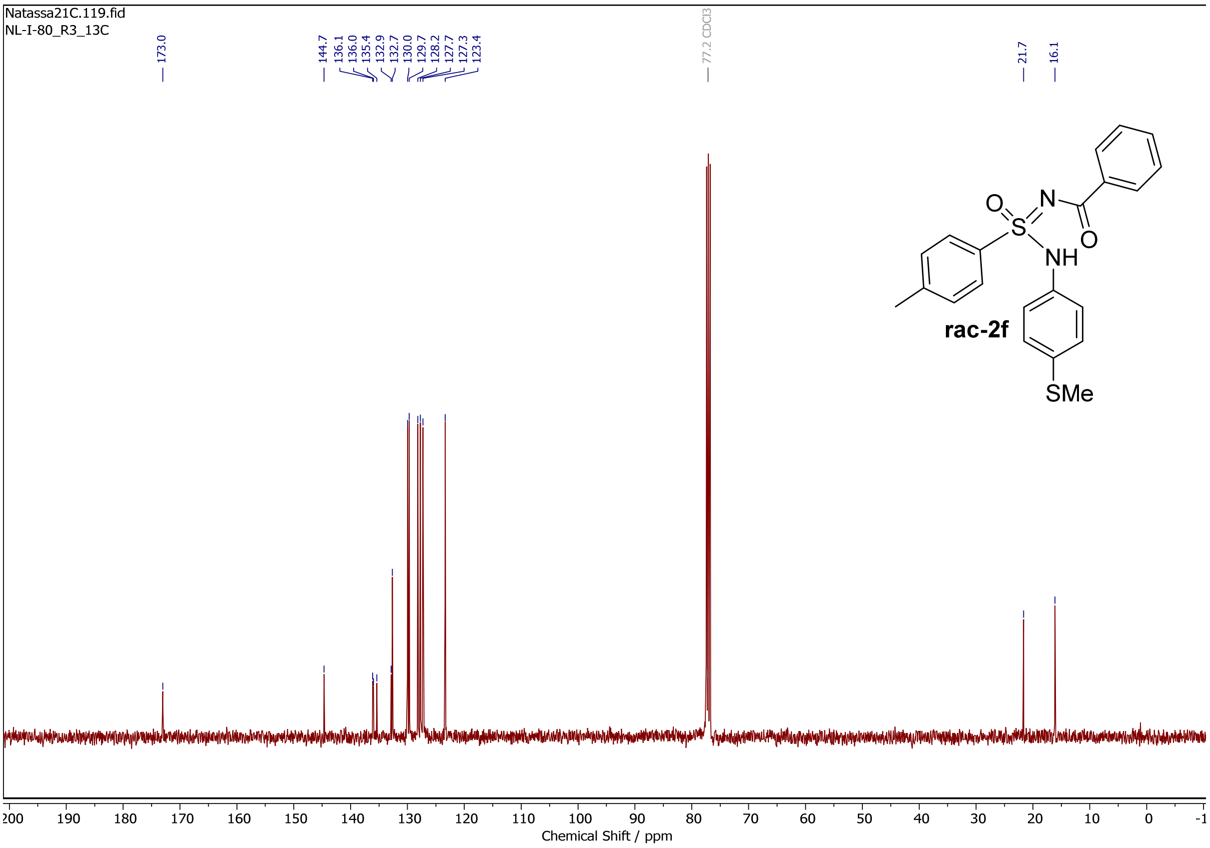

Figure S15. ^{13}C NMR (101 MHz) spectra of compound **rac-2f** ($CDCl_3$, 298 K).

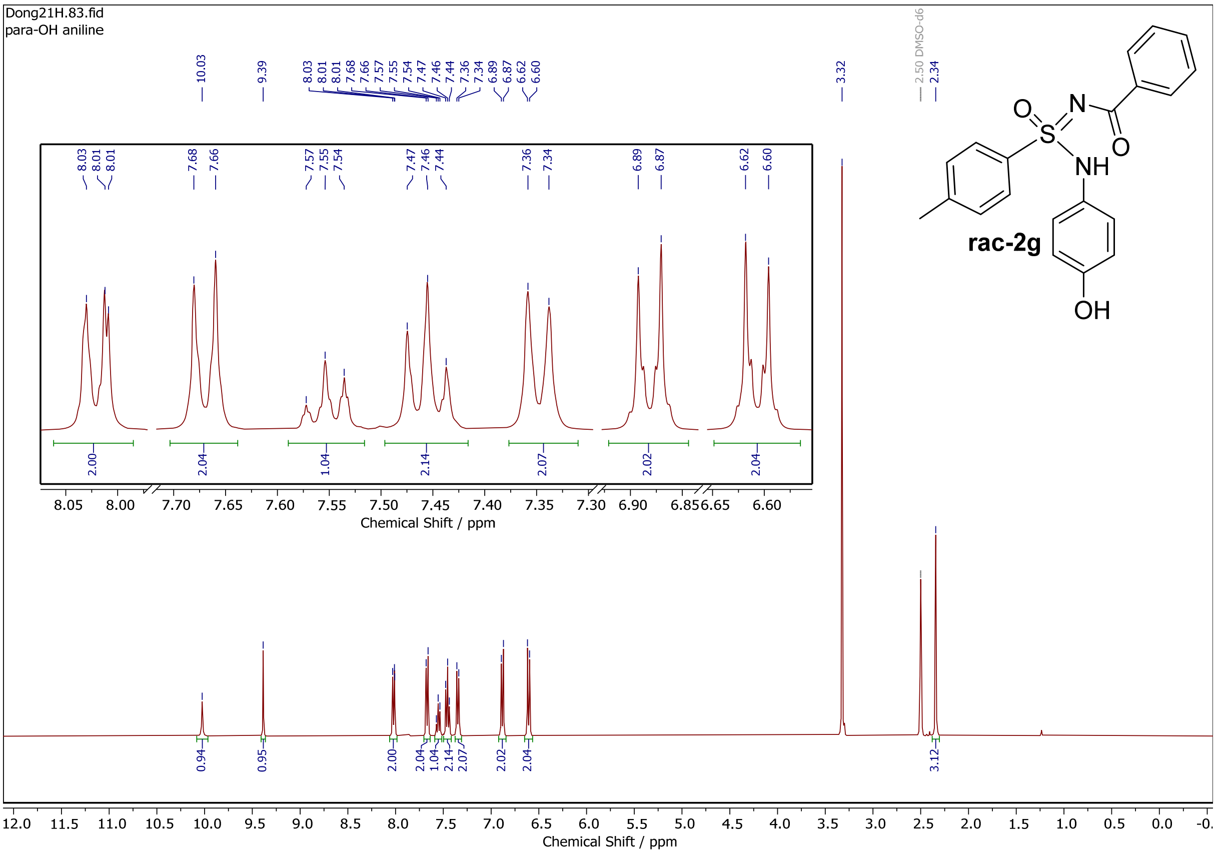

Figure S16. ^{1}H NMR (400 MHz) spectra of compound **rac-2g** (DMSO-d_6, 298 K).

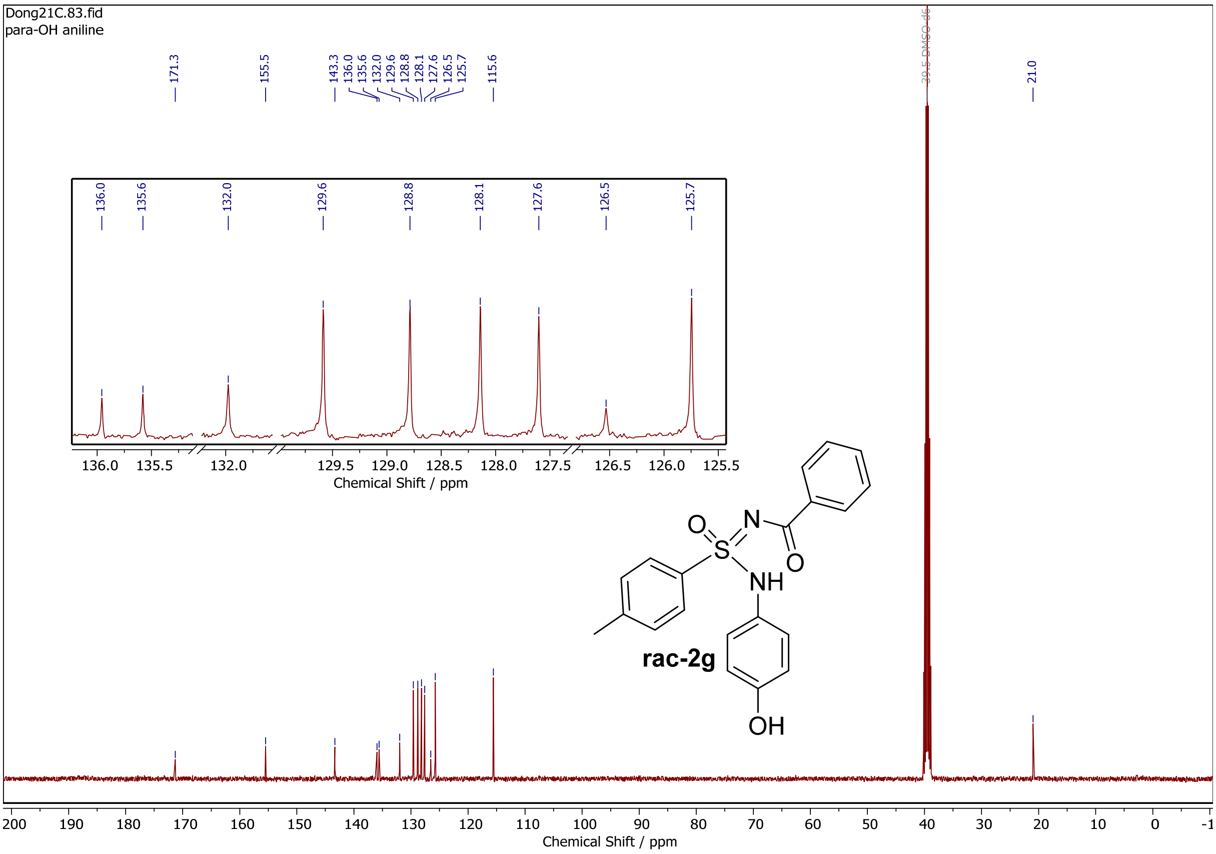

Figure S17. ^{13}C NMR (101 MHz) spectra of compound **rac-2g** (DMSO-d_6, 298 K).

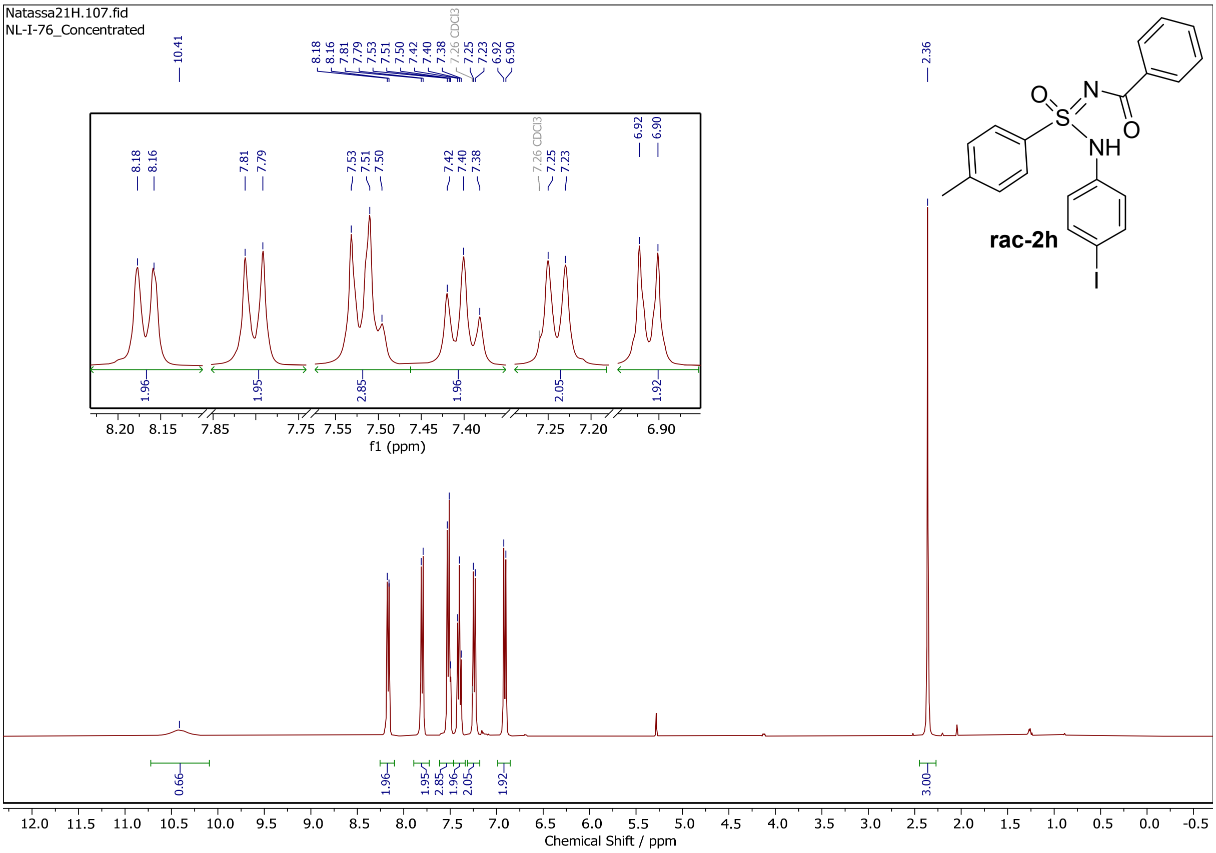

Figure S18. ^{1}H NMR (400 MHz) spectra of compound **rac-2h** ($CDCl_3$, 298 K).

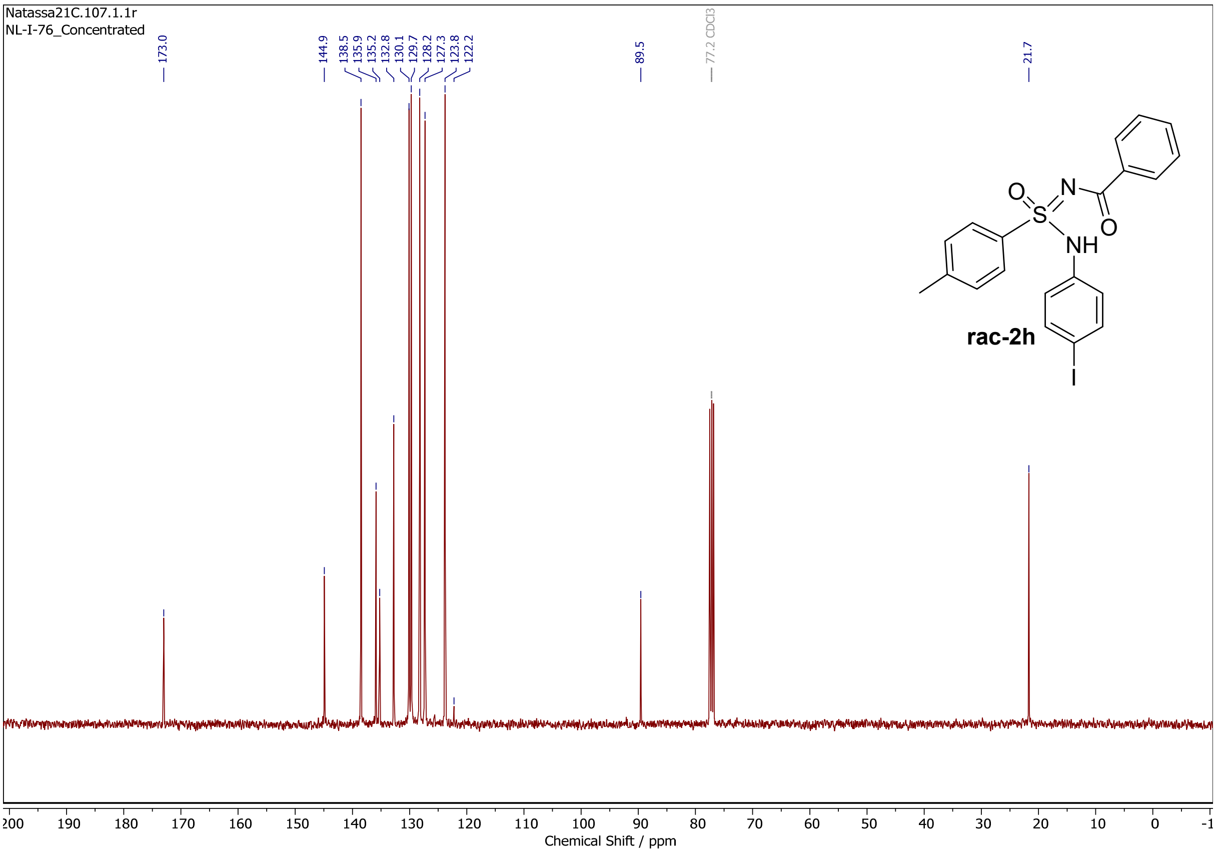

Figure S19. ^{13}C NMR (101 MHz) spectra of compound **rac-2h** ($CDCl_3$, 298 K).

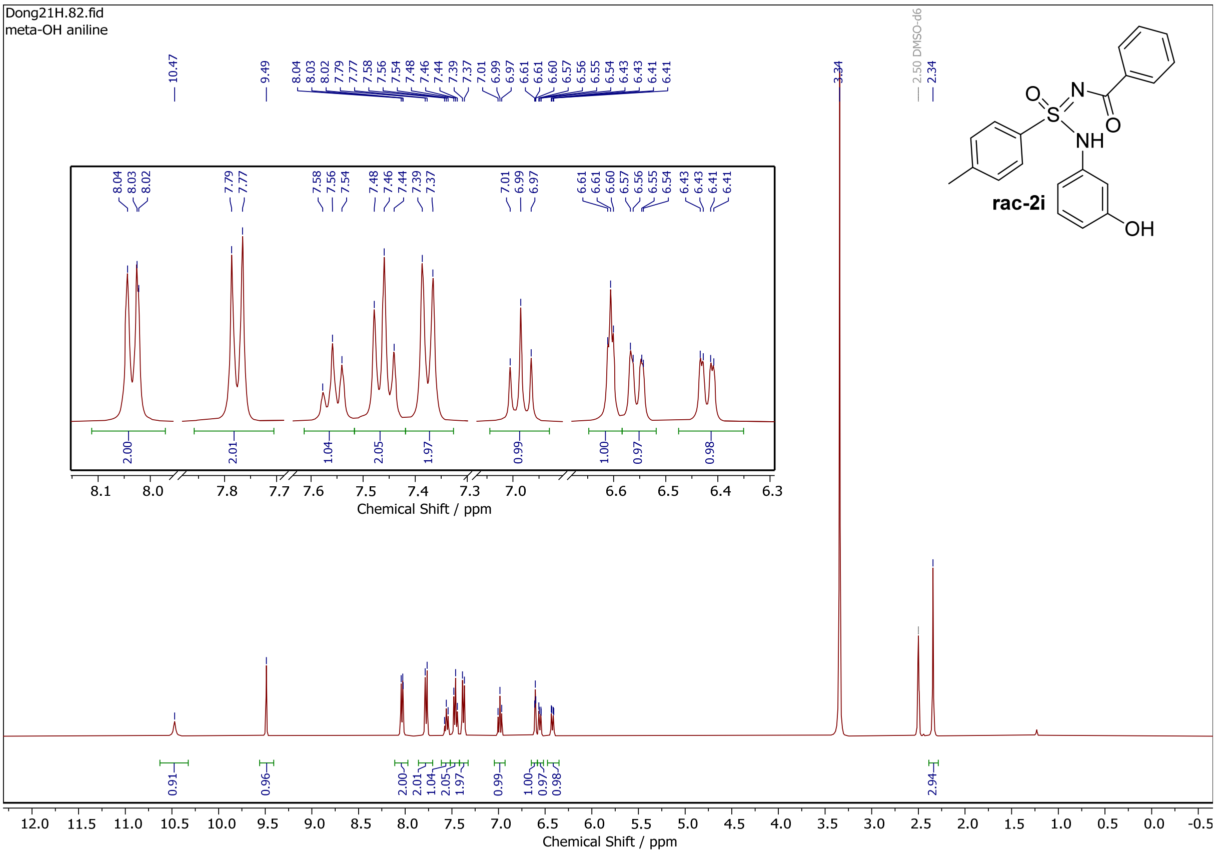

Figure S20. ^{1}H NMR (400 MHz) spectra of compound **rac-2i** (DMSO-d_6, 298 K).

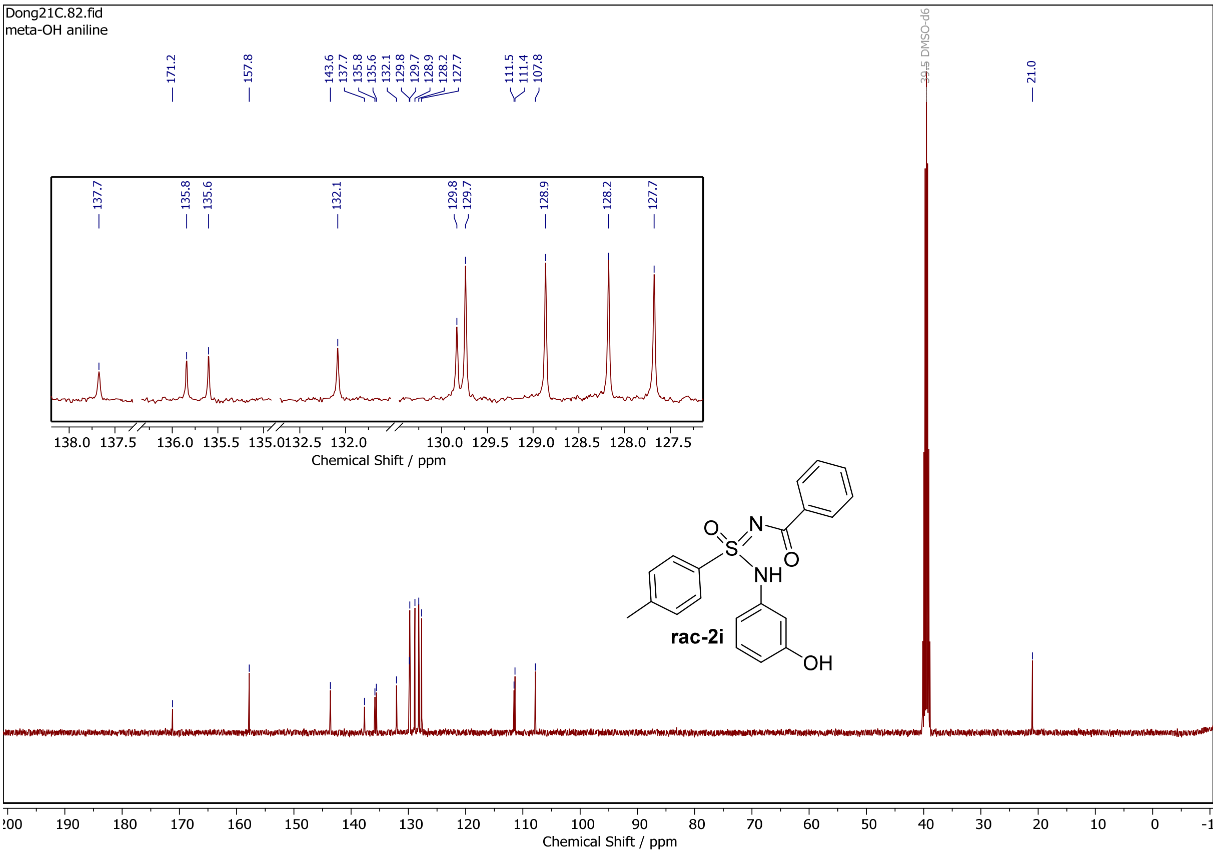

Figure S21. ^{13}C NMR (101 MHz) spectra of compound **rac-2i** (DMSO-d_6, 298 K).

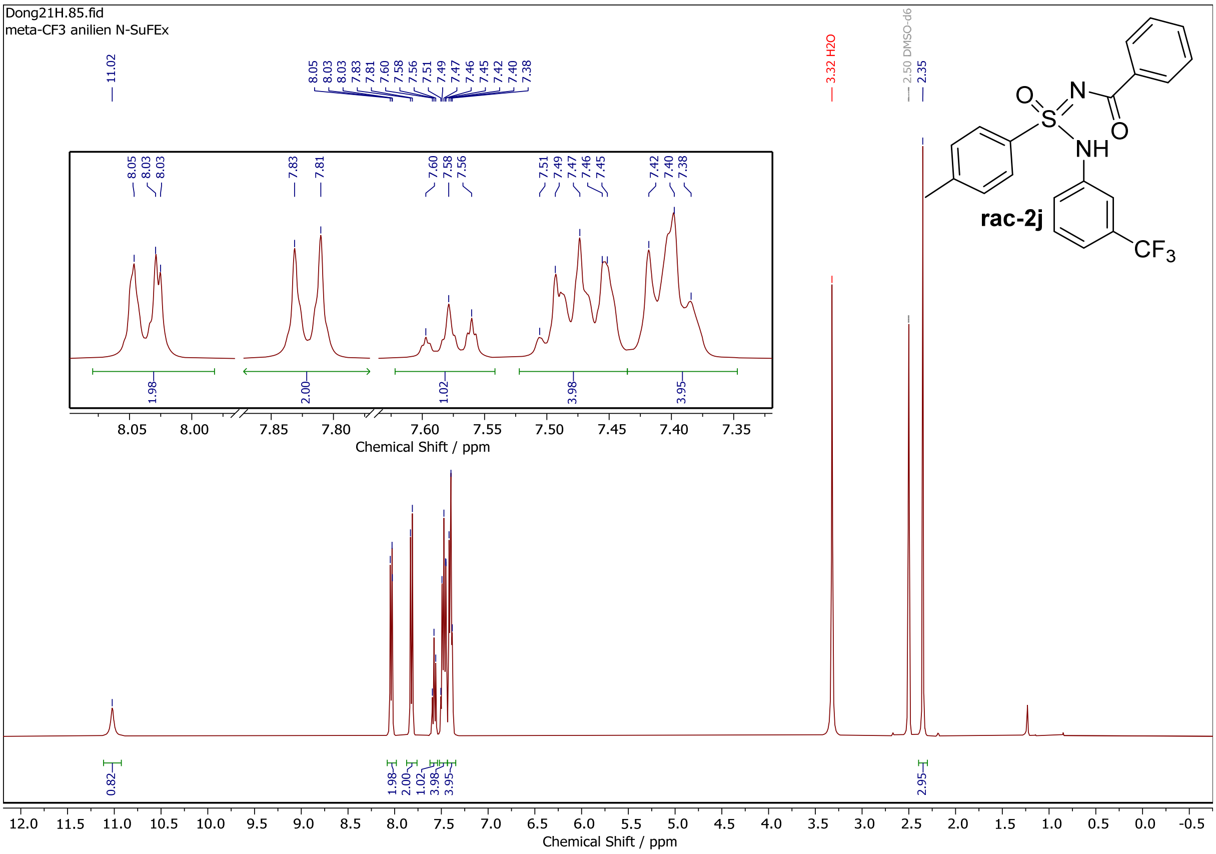

Figure S22. ^{1}H NMR (400 MHz) spectra of compound **rac-2j** (DMSO-d_6, 298 K).

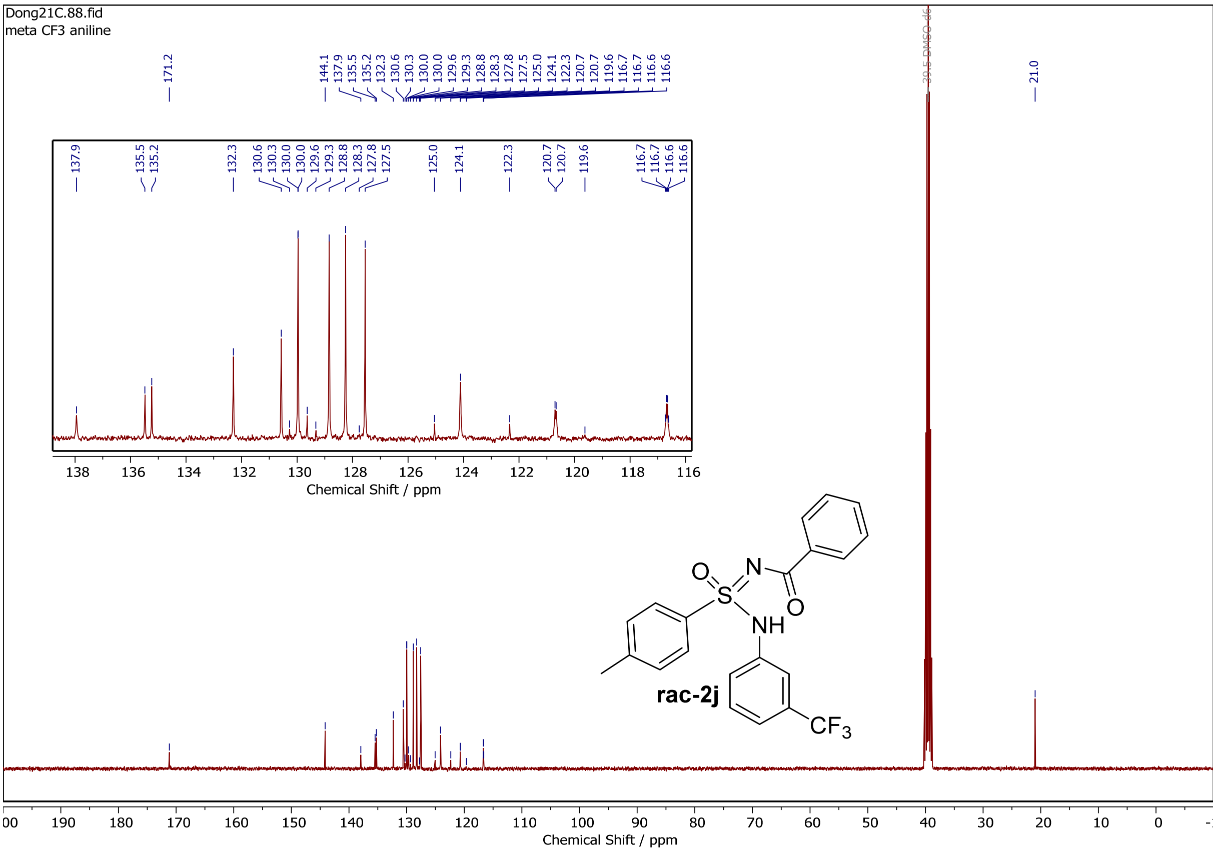

Figure S23. ^{13}C NMR (101 MHz) spectra of compound **rac-2j** (DMSO-d_6, 298 K).

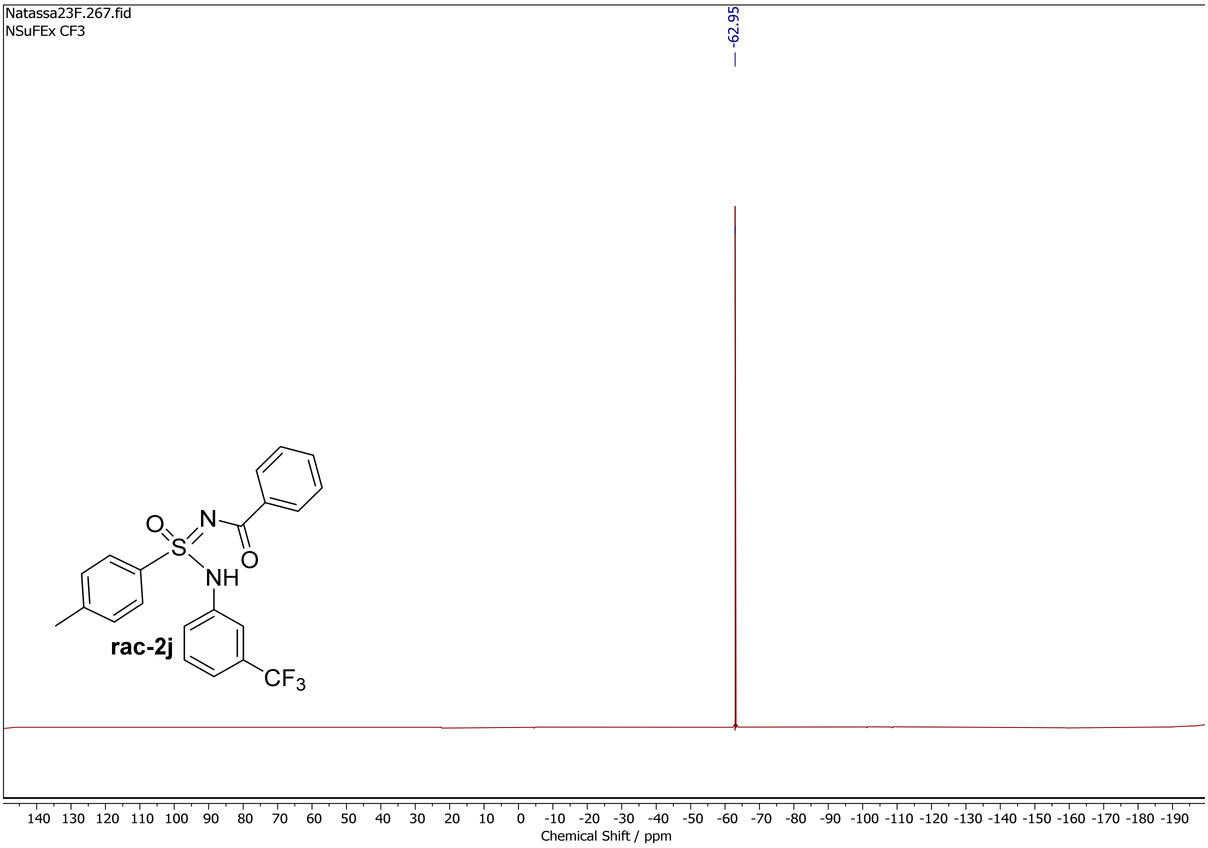

Figure S24. ^{19}F NMR (377 MHz) spectra of compound **rac-2j** ($CDCl_3$, 298 K).

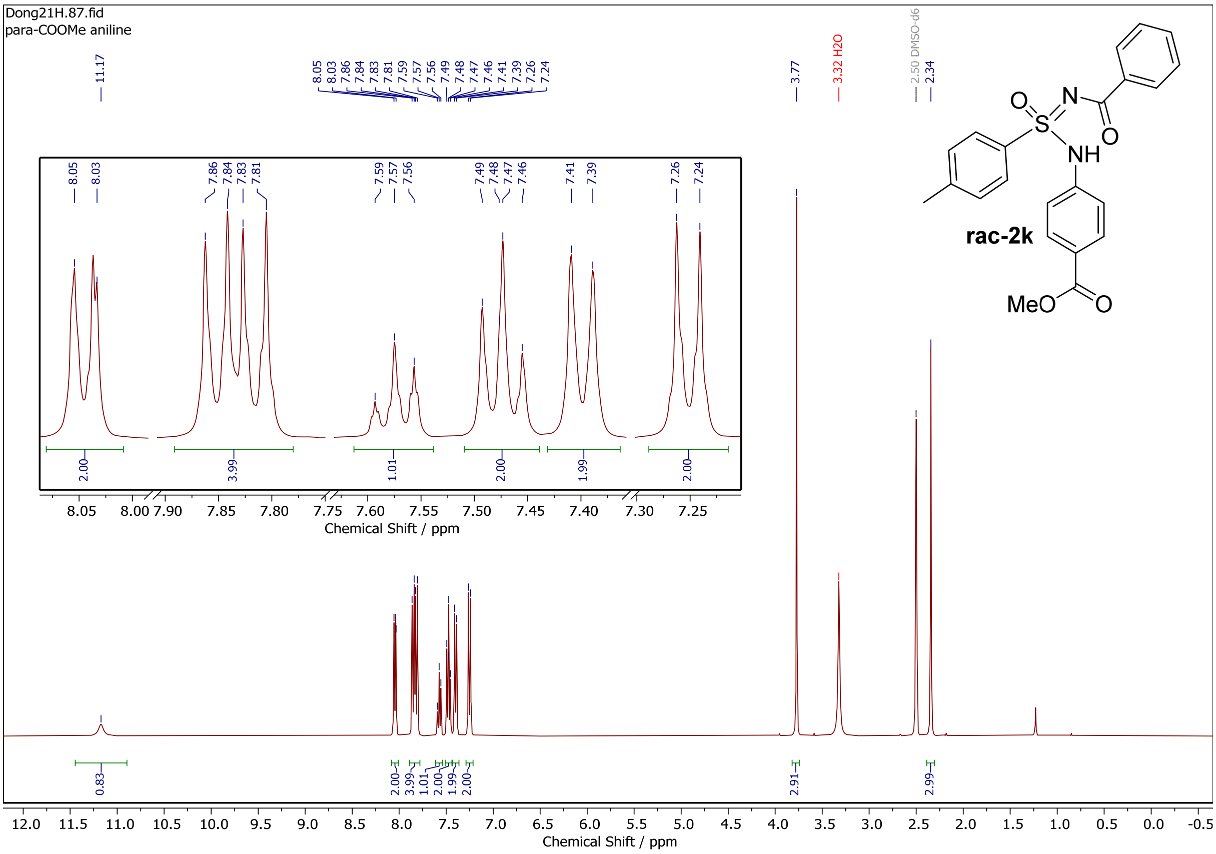

Figure S25. ^{1}H NMR (400 MHz) spectra of compound **rac-2k** (DMSO-d_6, 298 K).

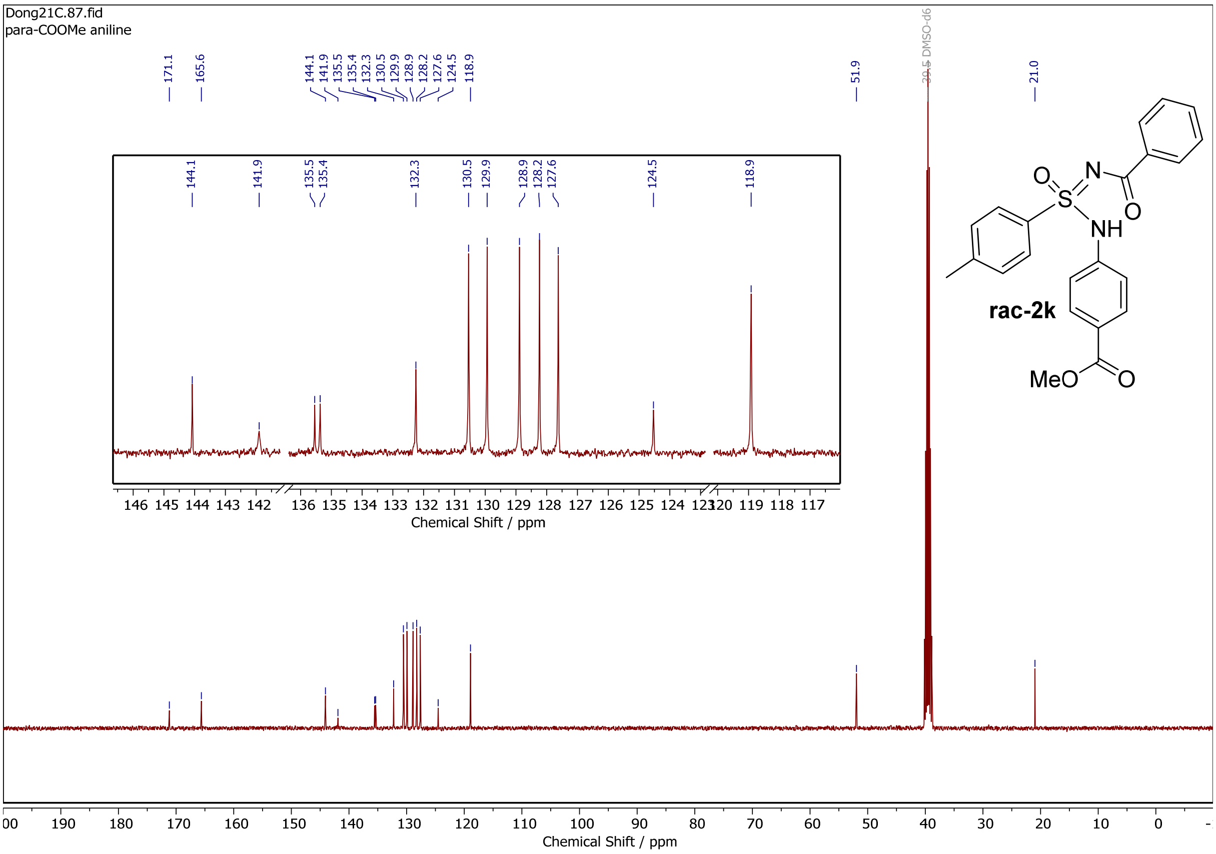

Figure S26. ^{13}C NMR (101 MHz) spectra of compound **rac-2k** (DMSO-d_6, 298 K).

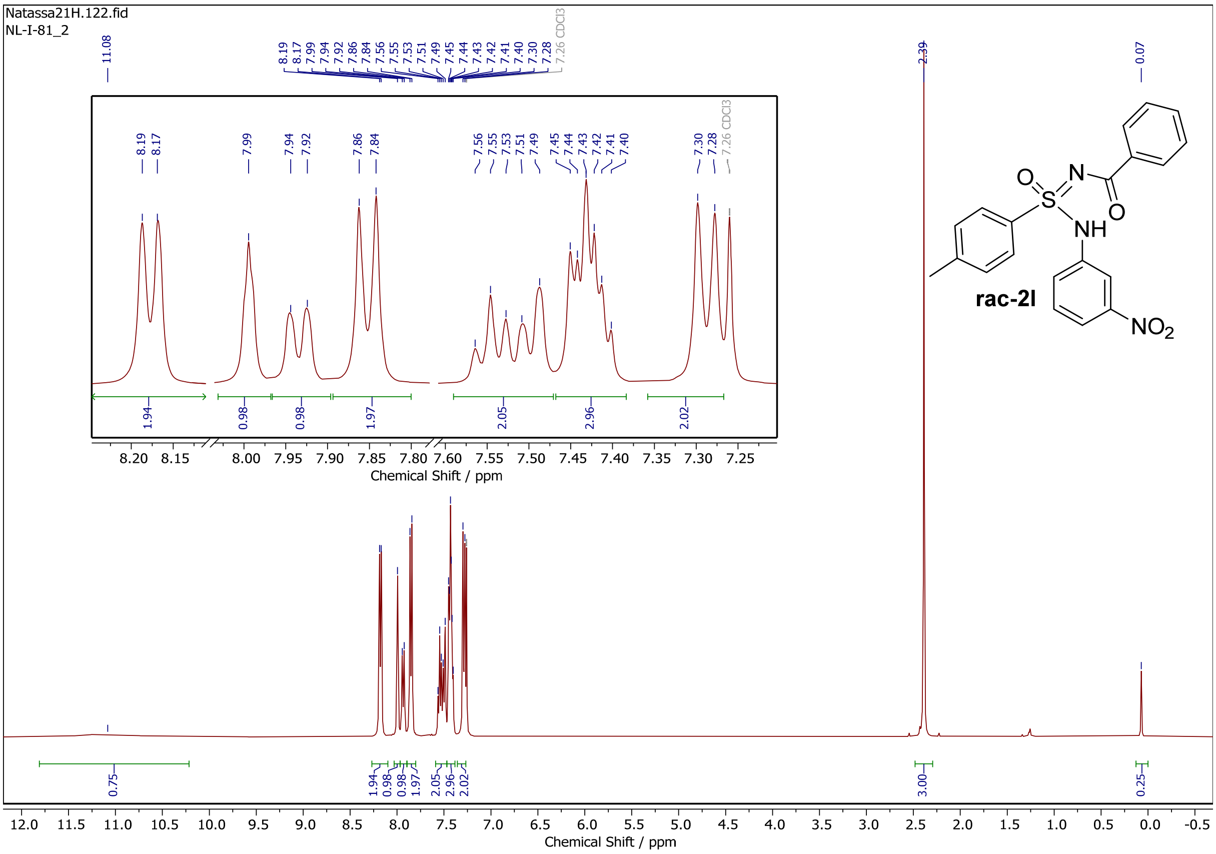

Figure S27. ^{1}H NMR (400 MHz) spectra of compound **rac-2l** ($CDCl_3$, 298 K).

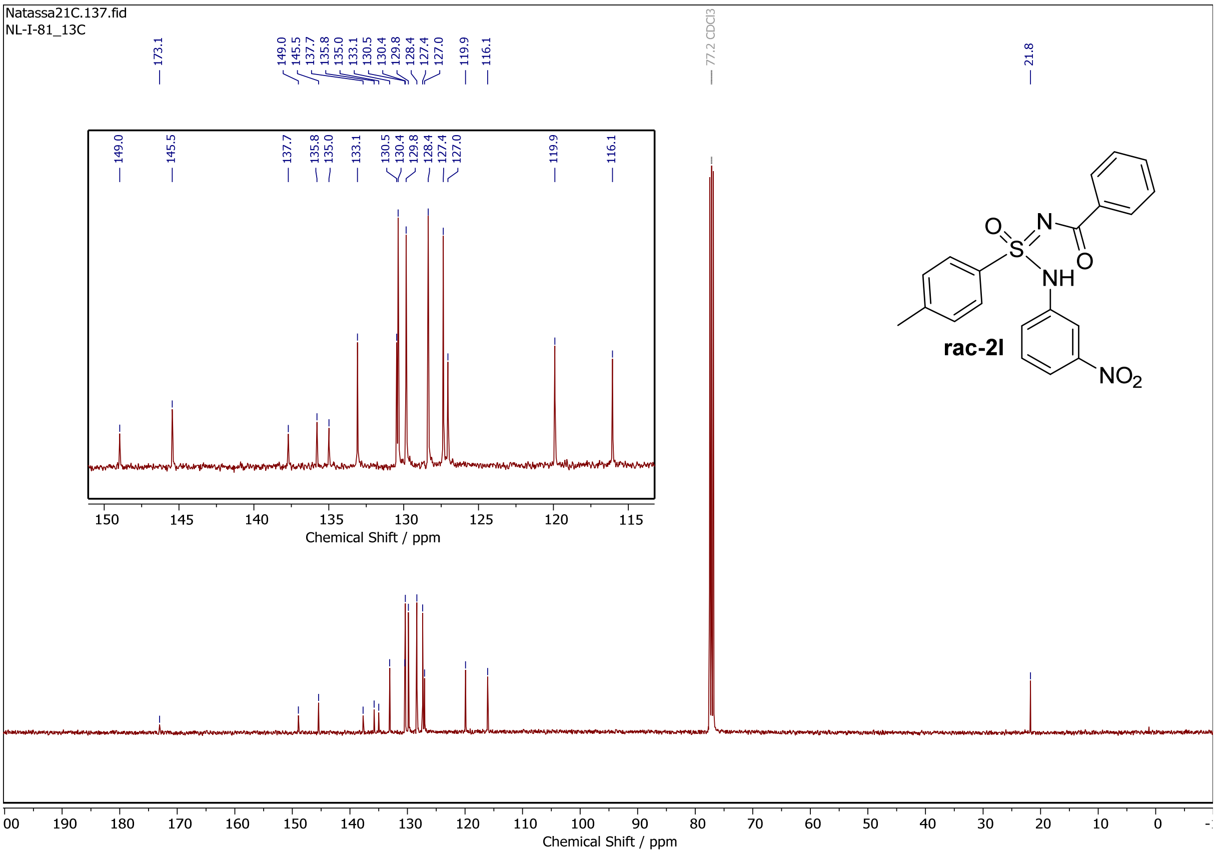

Figure S28. ^{13}C NMR (101 MHz) spectra of compound **rac-2l** ($CDCl_3$, 298 K).

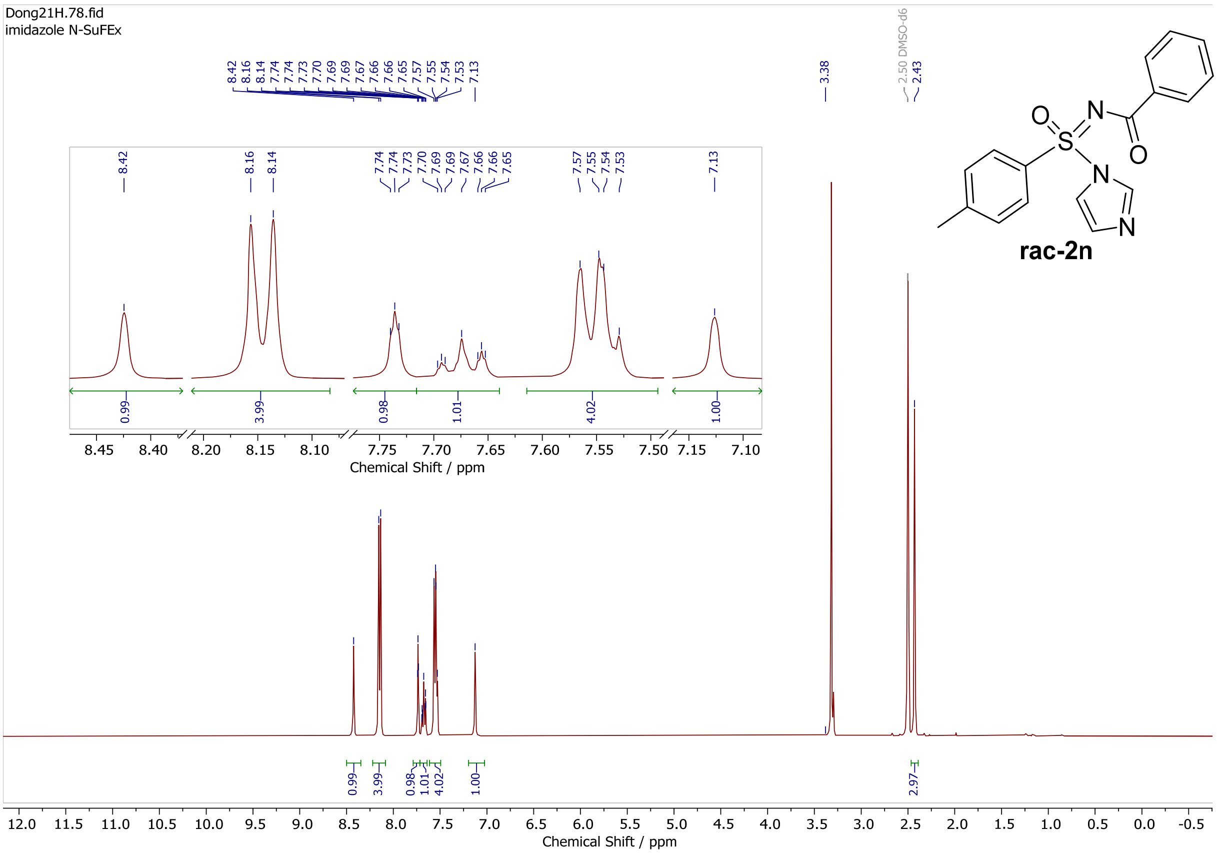

Figure S29. ^{1}H NMR (400 MHz) spectra of compound **rac-2n** (DMSO-d_6, 298 K).

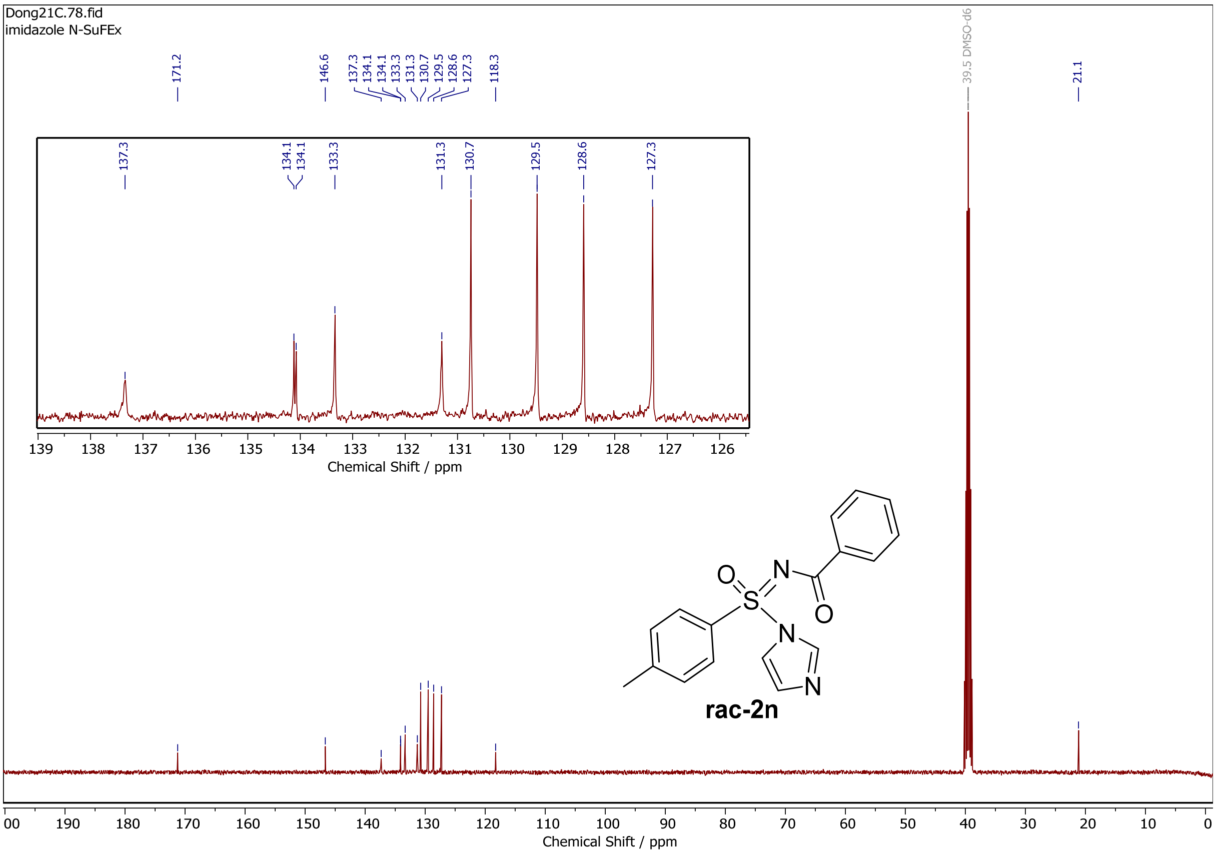

Figure S30. ^{13}C NMR (101 MHz) spectra of compound **rac-2n** (DMSO-d_6, 298 K).

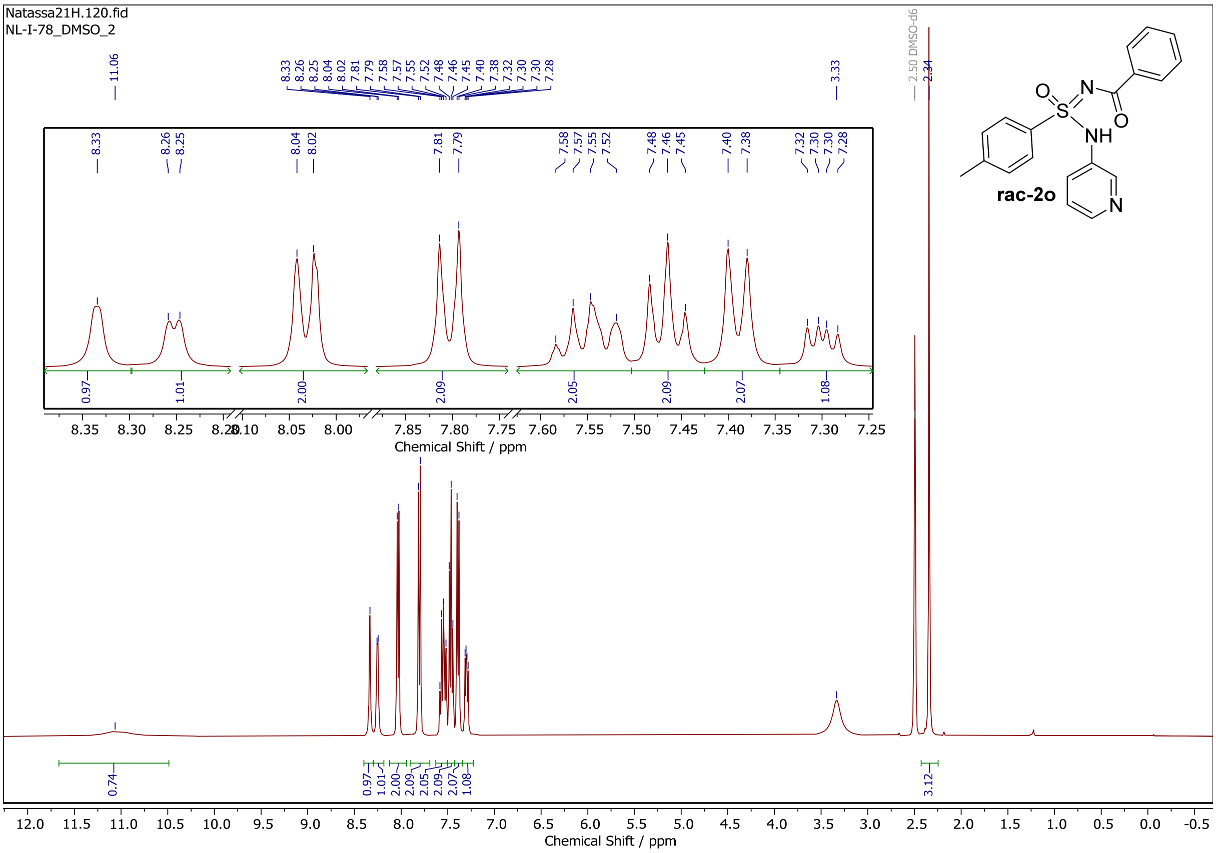

Figure S31. ^{1}H NMR (400 MHz) spectra of compound **rac-2o** (DMSO-d_6, 298 K).

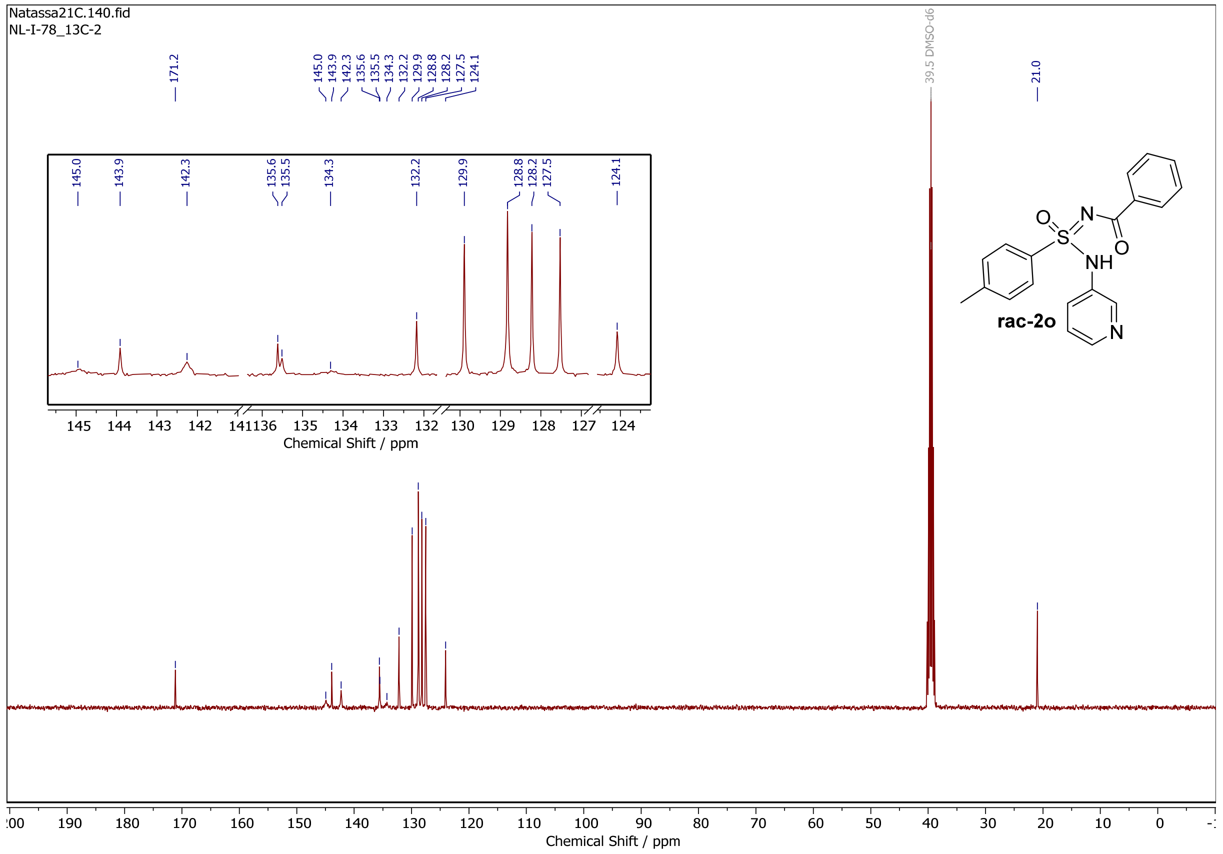

Figure S32. ^{13}C NMR (101 MHz) spectra of compound **rac-2o** (DMSO-d_6, 298 K).

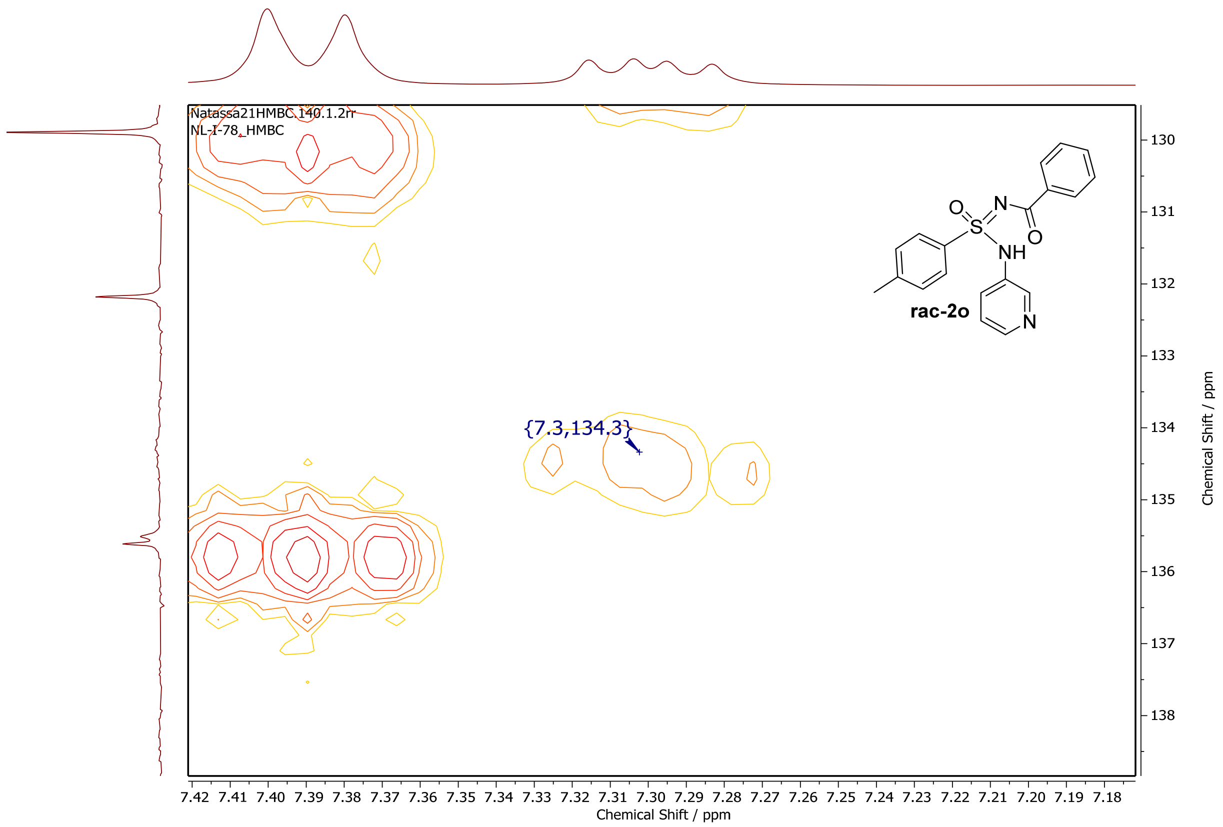

Figure S33. HMBC spectra of compound **rac-2o** (DMSO-d_6, 298 K)

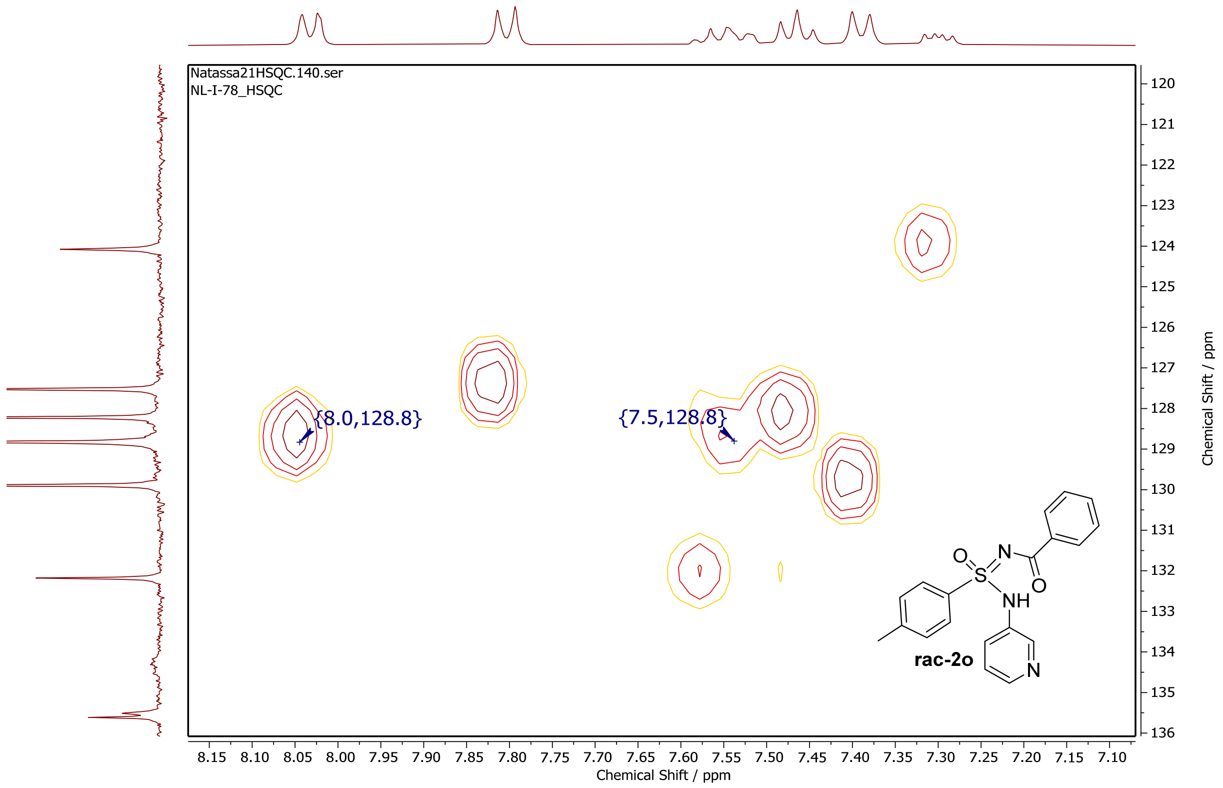

Figure S34. HSQC spectra of compound **rac-2o** (DMSO-d_6, 298 K)

N-SuFEx reactions between chiral *(R)*-1 and aromatic amines.

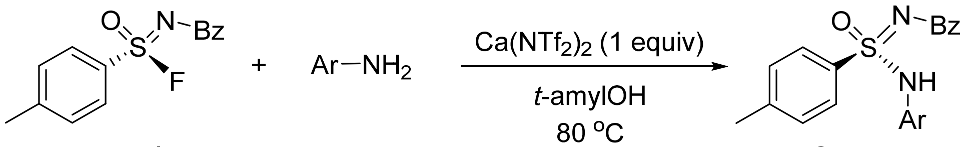

rac-1 **rac- 2**

General procedure. Reactions were performed following reactions conditions of the corresponding racemic general procedures A or B using (*R*)-**1** (>99% *ee*, 0.125 mmol, 35 mg, 1 equiv). The reaction product was isolated and the *ee* was determined by chiral HPLC.

HPLC data

(*rac*)-2a

HPLC (Chiralpak IA, *n*-hex/ IPA = 97.5:2.5, flow rate = 0.5 ml/min, l = 240 nm)

tR = 115.8 min, 131.4 min

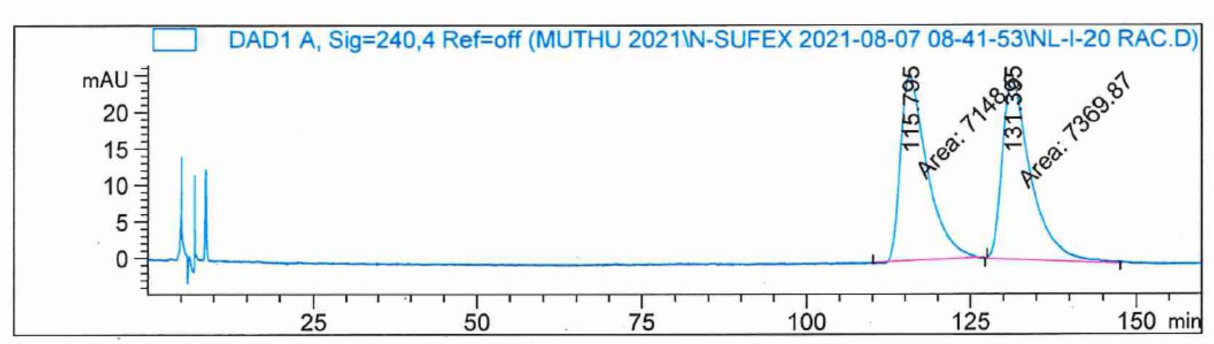

Signal 1: DAD1 A, Sig=240,4 Ref=off

Peak #	RetTime [min]	Sig	Type	Area [mAU*s]	Height [mAU]	Area %
1	115.795	1	MM	7148.60010	25.27129	49.2380
2	131.395	1	MM	7369.87256	24.56272	50.7620
Totals :				1.45185e4	49.83401	

(*S*)-2a, *ee* = >99%

tR = 113.7 min

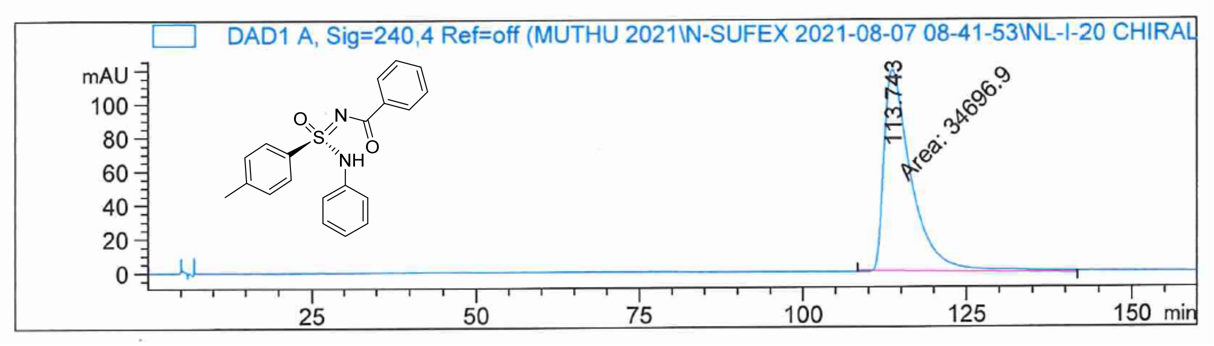

Signal 1: DAD1 A, Sig=240,4 Ref=off

Peak #	RetTime [min]	Sig	Type	Area [mAU*s]	Height [mAU]	Area %
1	113.743	1	MM	3.46969e4	119.17299	100.0000
Totals :				3.46969e4	119.17299	

(*rac*)-2b

HPLC (Chiralpak IA, *n*-hex/ IPA = 90:10, flow rate = 0.5 ml/min, l = 240 nm)

tR = 57.8 min, 72.1 min

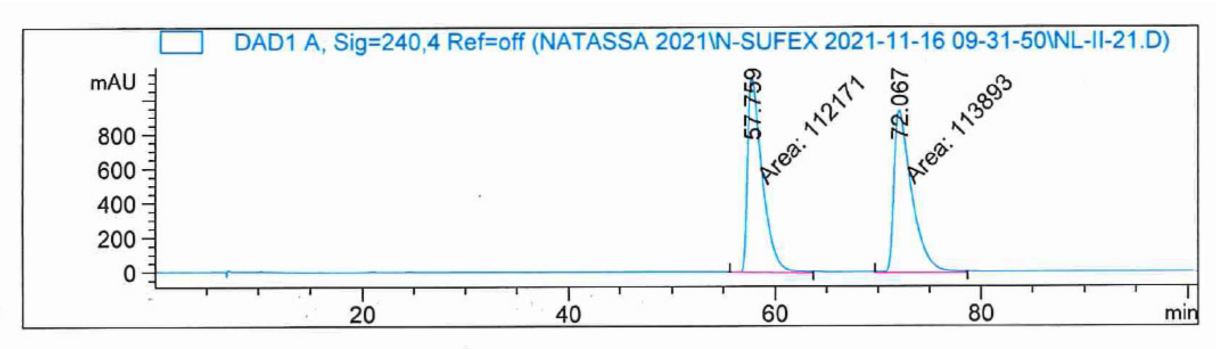

Signal 1: DAD1 A, Sig=240,4 Ref=off

Peak #	RetTime [min]	Sig	Type	Area [mAU*s]	Height [mAU]	Area %
1	57.759	1	MM	1.12171e5	1134.52991	49.6191
2	72.067	1	MM	1.13893e5	946.31818	50.3809
Totals :				2.26064e5	2080.84808	

(*S*)-2b, *ee* = 99%

tR = 59.5 min (minor), 72.4 (major)

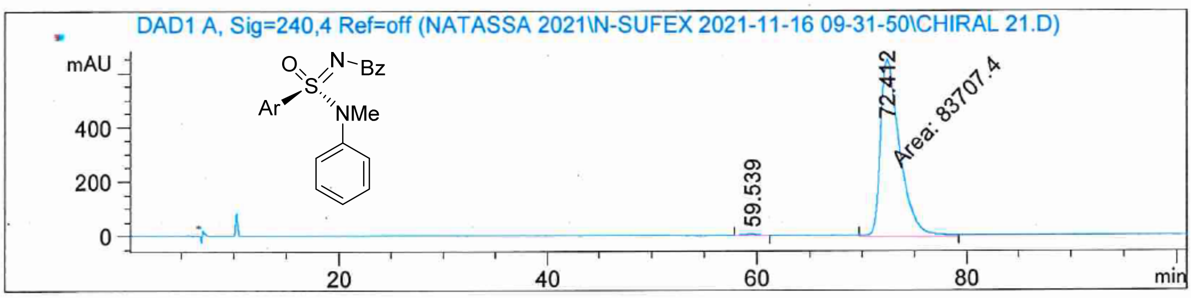

Signal 1: DAD1 A, Sig=240,4 Ref=off

Peak #	RetTime [min]	Sig	Type	Area [mAU*s]	Height [mAU]	Area %
1	59.539	1	BB	547.94055	5.67110	0.6503
2	72.412	1	MM	8.37074e4	652.08209	99.3497
Totals :				8.42553e4	657.75319	

(*rac*)-2c

HPLC (Chiralpak IA, *n*-hex/DCM/IPA = 90:10:2.5, flow rate = 1.0 ml/min, l = 240 nm)

tR = 39.4 min, 44.8 min

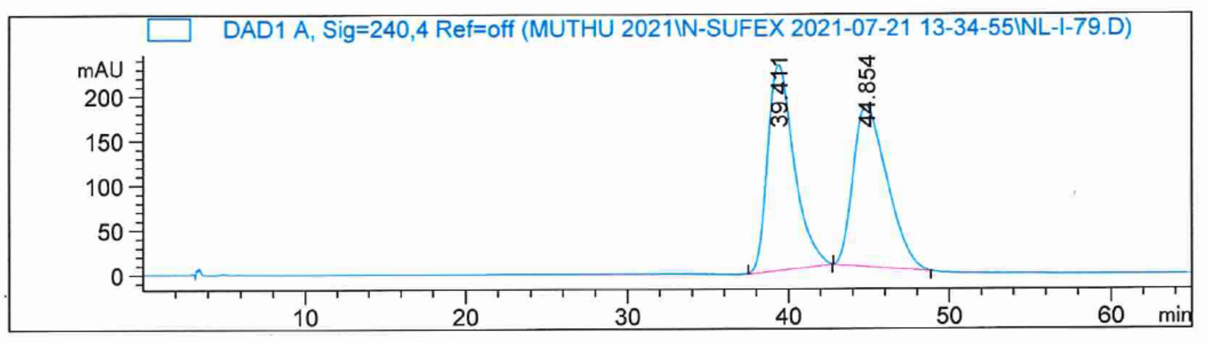

Signal 1: DAD1 A, Sig=240,4 Ref=off

Peak #	RetTime [min]	Sig	Type	Area [mAU*s]	Height [mAU]	Area %
1	39.411	1	BB	2.62596e4	230.31291	49.9753
2	44.854	1	BB	2.62856e4	180.32578	50.0247
Totals :				5.25451e4	410.63869	

(*S*)-2c, *ee* = 98%

tR = 39.6 min (minor), 43.9 (major)

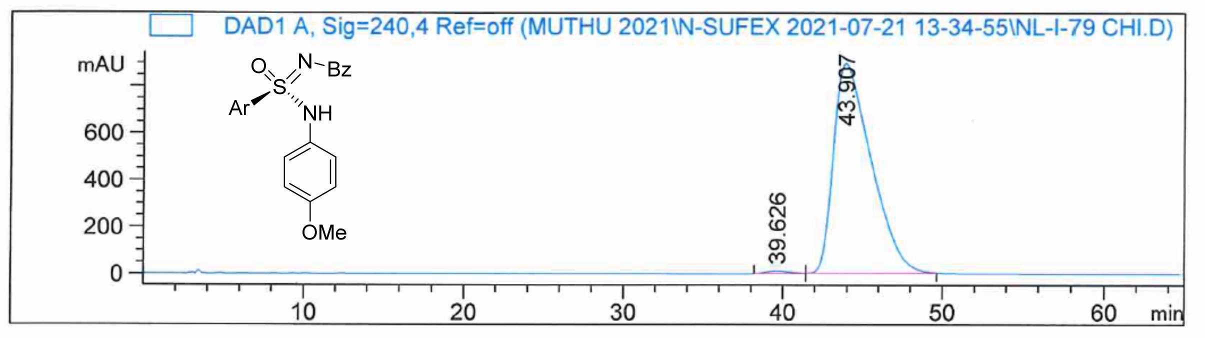

Signal 1: DAD1 A, Sig=240,4 Ref=off

Peak #	RetTime [min]	Sig	Type	Area [mAU*s]	Height [mAU]	Area %
1	39.626	1	BB	1084.03748	11.67913	0.7293
2	43.907	1	BB	1.47551e5	896.60876	99.2707
Totals :				1.48635e5	908.28790	

(*rac*)-2d

HPLC (Chiralpak IA, *n*-hex/ IPA = 90:10, flow rate = 0.5 ml/min, l = 240 nm)

tR = 90.6 min, 112.7 min

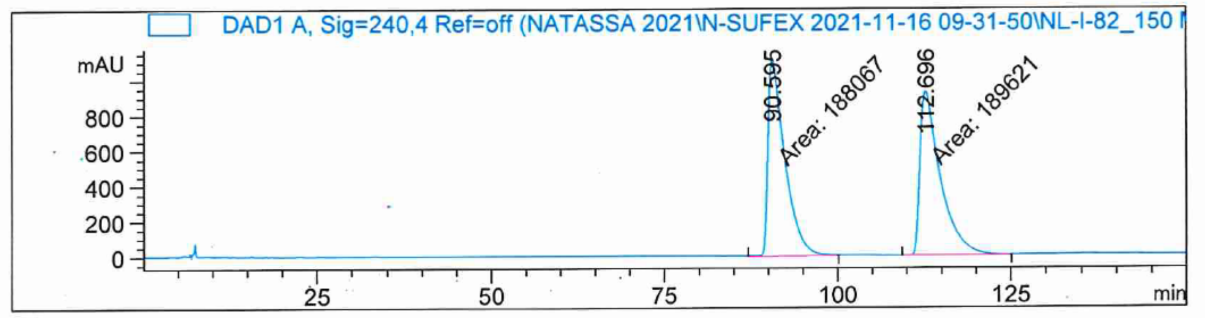

Signal 1: DAD1 A, Sig=240,4 Ref=off

Peak #	RetTime [min]	Sig	Type	Area [mAU*s]	Height [mAU]	Area %
1	90.595	1	MM	1.88067e5	1125.17505	49.7942
2	112.696	1	MM	1.89621e5	929.44958	50.2058
Totals :				3.77688e5	2054.62463	

(*S*)-2d, *ee* = 98%

tR = 93.9 min (minor), 112.9 min (major)

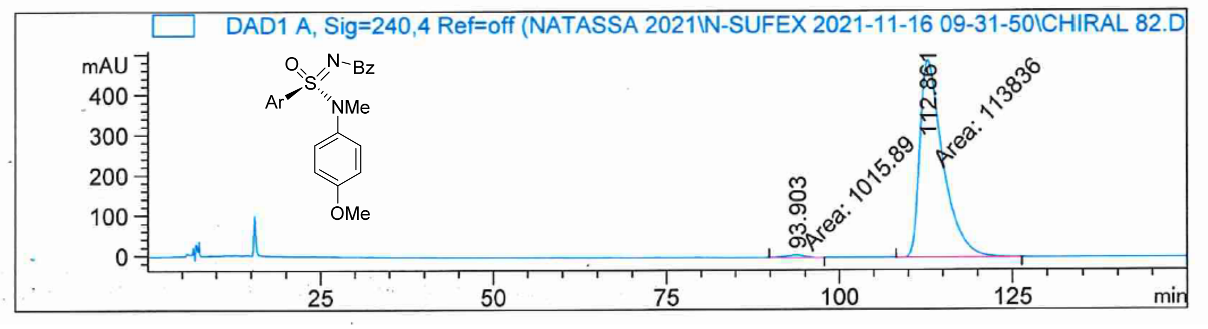

Signal 1: DAD1 A, Sig=240,4 Ref=off

Peak #	RetTime [min]	Sig	Type	Area [mAU*s]	Height [mAU]	Area %
1	93.903	1	MM	1015.88959	5.84833	0.8845
2	112.861	1	MM	1.13836e5	490.24481	99.1155
Totals :				1.14852e5	496.09314	

(*rac*)-2e

HPLC (Chiralpak IA, *n*-hex/ IPA = 90:10, flow rate = 0.5 ml/min, l = 280 nm)

tR = 38.0 min, 41.9 min

Extensive screening of HPLC conditions did not allow to achieve baseline separation of enantiomers in racemate, but the separation was sufficient to estimate the *ee* value.

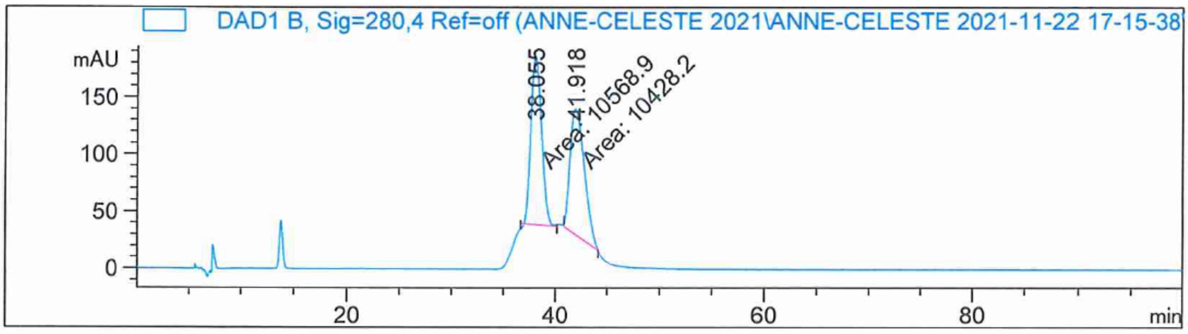

Signal 1: DAD1 B, Sig=280,4 Ref=off

Peak #	RetTime [min]	Sig	Type	Area [mAU*s]	Height [mAU]	Area %
1	38.055	1	MM	1.05689e4	146.19797	50.3350
2	41.918	1	MM	1.04282e4	110.48151	49.6650
Totals :				2.09970e4	256.67947	

(*S*)-2e, *ee* = >99%

tR = 38.1 min

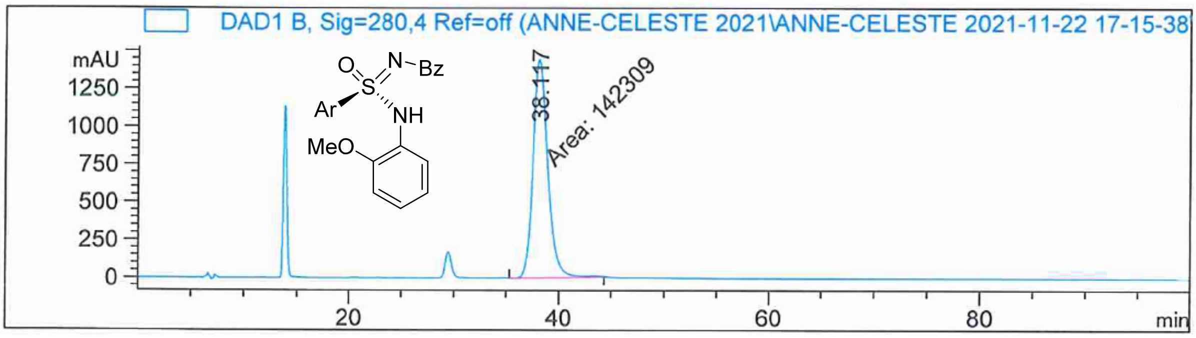

Signal 1: DAD1 B, Sig=280,4 Ref=off

Peak #	RetTime [min]	Sig	Type	Area [mAU*s]	Height [mAU]	Area %
1	38.117	1	MM	1.42309e5	1448.50708	100.0000
Totals :				1.42309e5	1448.50708	

(*rac*)-2f

HPLC (Chiralpak IA, *n*-hex/DCM/formic acid = 70:30:0.1%, flow rate = 0.5 ml/min, l = 260 nm)

tR = 29.9 min, 33.9 min

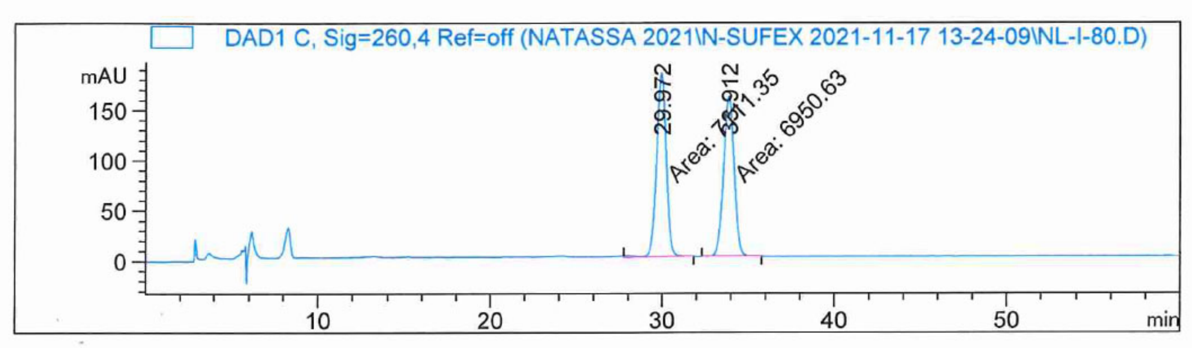

Signal 1: DAD1 C, Sig=260,4 Ref=off

Peak #	RetTime [min]	Sig	Type	Area [mAU*s]	Height [mAU]	Area %
1	29.972	1	MM	7111.34668	182.78667	50.5715
2	33.912	1	MM	6950.63184	159.07658	49.4285
Totals :				1.40620e4	341.86325	

(*S*)-2f, *ee* = >99%

tR = 33.8 min

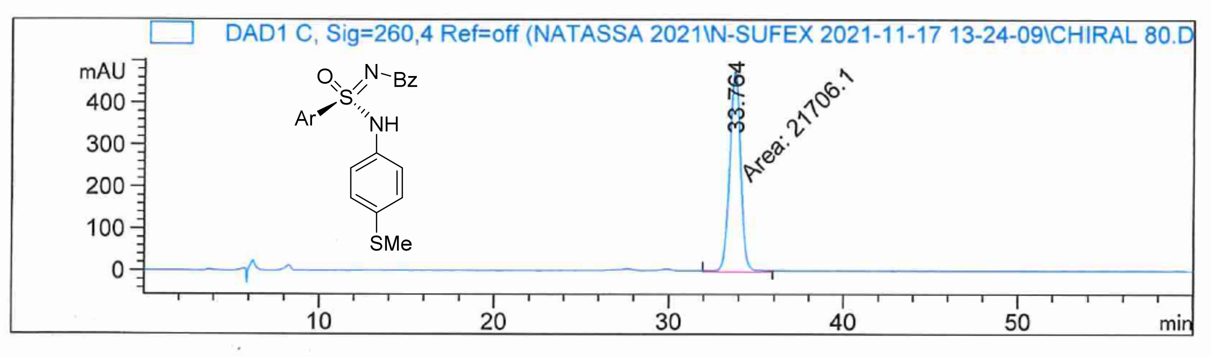

Signal 1: DAD1 C, Sig=260,4 Ref=off

Peak #	RetTime [min]	Sig	Type	Area [mAU*s]	Height [mAU]	Area %
1	33.764	1	MM	2.17061e4	481.12354	100.0000
Totals :				2.17061e4	481.12354	

(*rac*)-2g

HPLC (Chiralpak IA, *n*-hex/DCM/IPA = 80:20:5, flow rate = 0.5 ml/min, l = 240 nm)

tR = 53.6 min, 60.8 min

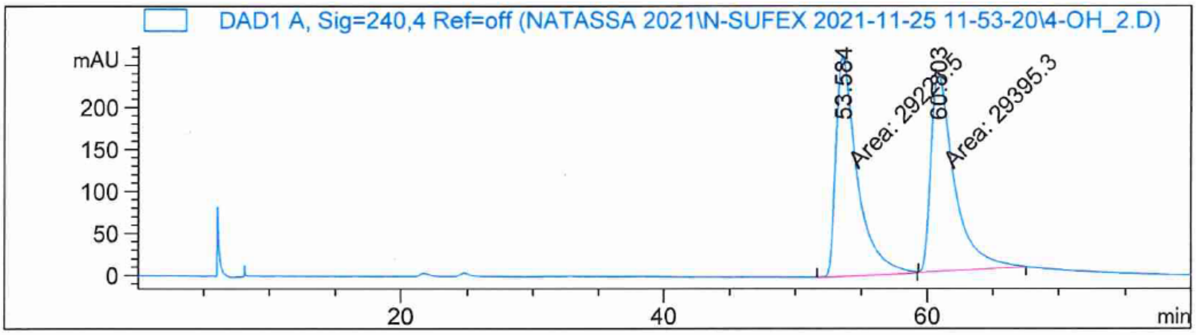

Signal 1: DAD1 A, Sig=240,4 Ref=off

Peak #	RetTime [min]	Sig	Type	Area [mAU*s]	Height [mAU]	Area %
1	53.584	1	MM	2.92215e4	260.90613	49.8518
2	60.803	1	MM	2.93953e4	236.21217	50.1482
Totals :				5.86168e4	497.11830	

(*S*)-2g, *ee* = >99%

tR = 53.9 min (minor), 59.0 min (major)

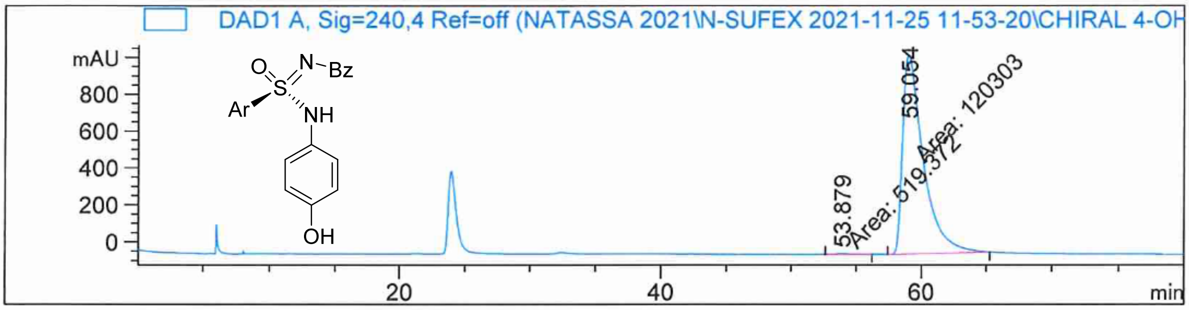

Signal 1: DAD1 A, Sig=240,4 Ref=off

Peak #	RetTime [min]	Sig	Type	Area [mAU*s]	Height [mAU]	Area %
1	53.879	1	MM	519.37195	3.80689	0.4299
2	59.054	1	MM	1.20303e5	1076.55920	99.5701
Totals :				1.20823e5	1080.36610	

(*rac*)-2h

HPLC (Chiralpak IA, *n*-hex/DCM/formic acid = 70:30:0.1%, flow rate = 0.5 ml/min, l = 260 nm)

tR = 28.6 min, 32.0 min

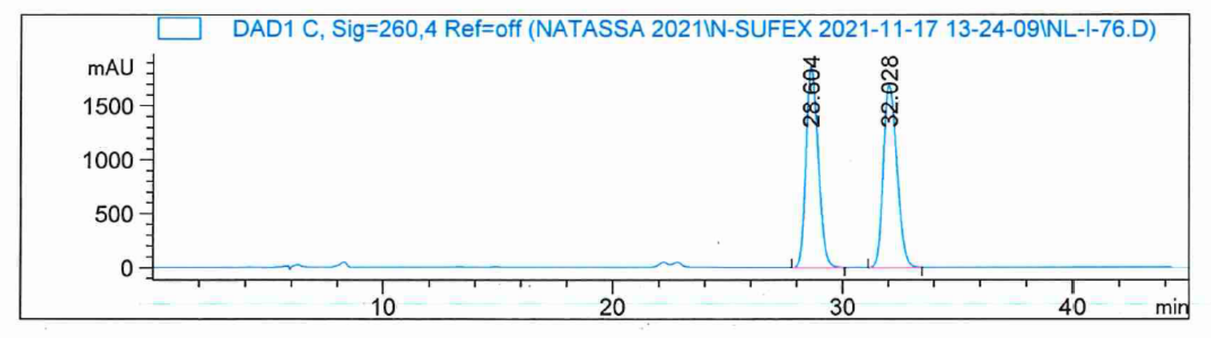

Signal 1: DAD1 C, Sig=260,4 Ref=off

Peak #	RetTime [min]	Sig	Type	Area [mAU*s]	Height [mAU]	Area %
1	28.604	1	VV	6.90772e4	1871.31213	49.6878
2	32.028	1	VV	6.99453e4	1688.21545	50.3122
Totals :				1.39022e5	3559.52759	

(*S*)-2h, *ee* = >99%

tR = 28.7 min (minor), 31.9 min (major)

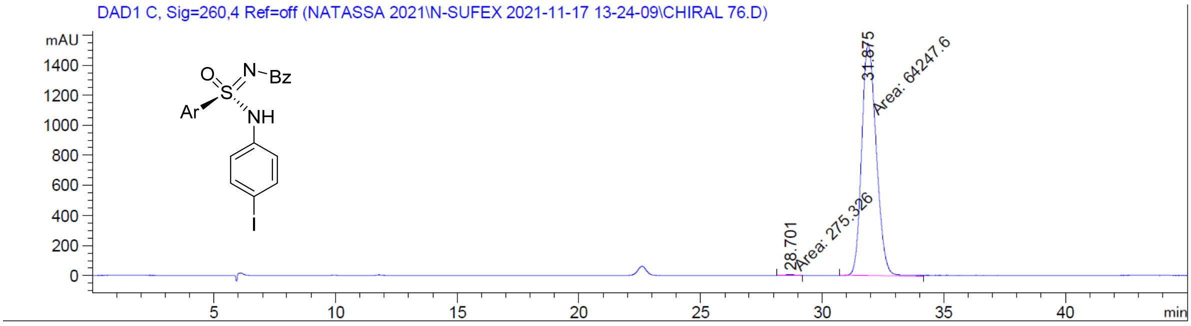

Signal 1: DAD1 C, Sig=260,4 Ref=off

Peak #	RetTime [min]	Type	Width [min]	Area [mAU*s]	Height [mAU]	Area %
1	28.701	MM	0.6665	275.32642	6.88509	0.4267
2	31.875	MM	0.6913	6.42476e4	1548.85608	99.5733
Totals :				6.45230e4	1555.74117	

(*rac*)-2j

HPLC (Chiralpak IA, *n*-hex/IPA/formic acid = 95:5:0.1%, flow rate = 0.5 ml/min, l = 260 nm)

tR = 66.0 min, 76.4 min

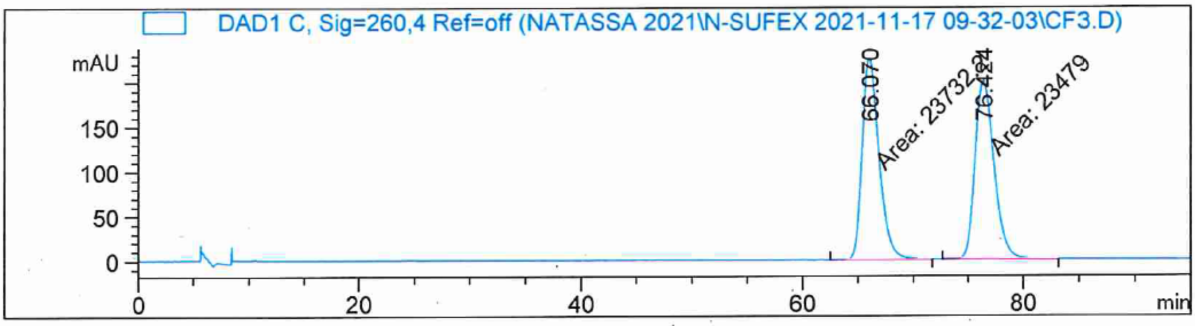

Signal 1: DAD1 C, Sig=260,4 Ref=off

Peak #	RetTime [min]	Sig	Type	Area [mAU*s]	Height [mAU]	Area %
1	66.070	1	MM	2.37322e4	225.94441	50.2682
2	76.424	1	MM	2.34790e4	199.42445	49.7318
Totals :				4.72112e4	425.36887	

(*S*)-2j, *ee* = >99%

tR = 65.8 min

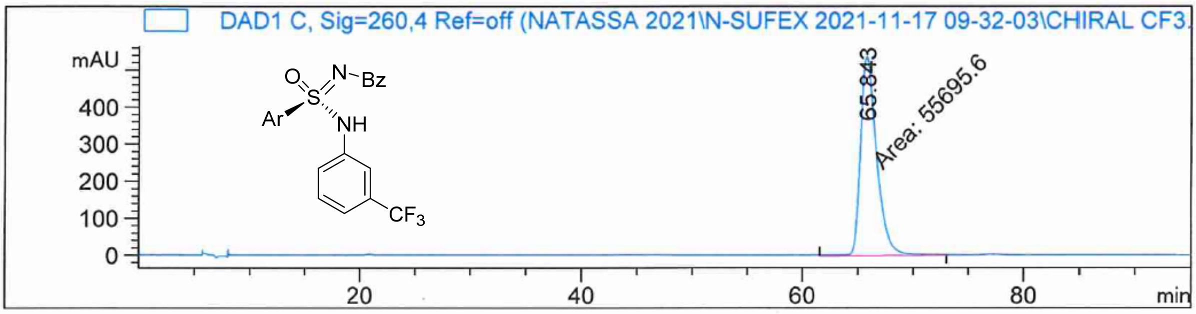

Signal 1: DAD1 C, Sig=260,4 Ref=off

Peak #	RetTime [min]	Sig	Type	Area [mAU*s]	Height [mAU]	Area %
1	65.843	1	MM	5.56956e4	539.78741	100.0000
Totals :				5.56956e4	539.78741	

(*rac*)-2k

HPLC (Chiralpak IA, *n*-hex/DCM/formic acid = 70:30:0.1%, flow rate = 0.5 ml/min, l = 280 nm)

tR = 30.6 min, 33.9 min

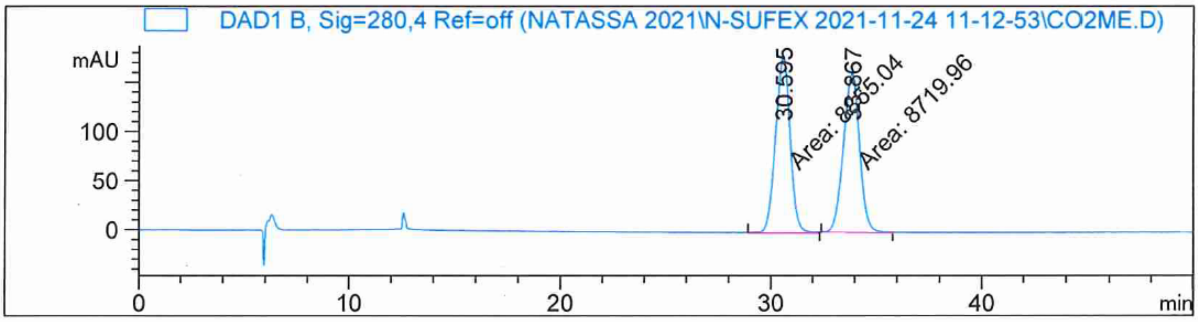


```
Signal 1: DAD1 B, Sig=280,4 Ref=off

Peak RetTime Sig Type    Area      Height     Area
  #   [min]             [mAU*s]     [mAU]       %
----|-------|---|----|----------|----------|--------|
   1  30.595   1  MM   8855.03711  179.70045  50.3843
   2  33.867   1  MM   8719.96094  161.88884  49.6157

Totals :               1.75750e4   341.58929
```

(*S*)-2k, *ee* = >99%

tR = 30.6 min (minor), 33.5 min (major)

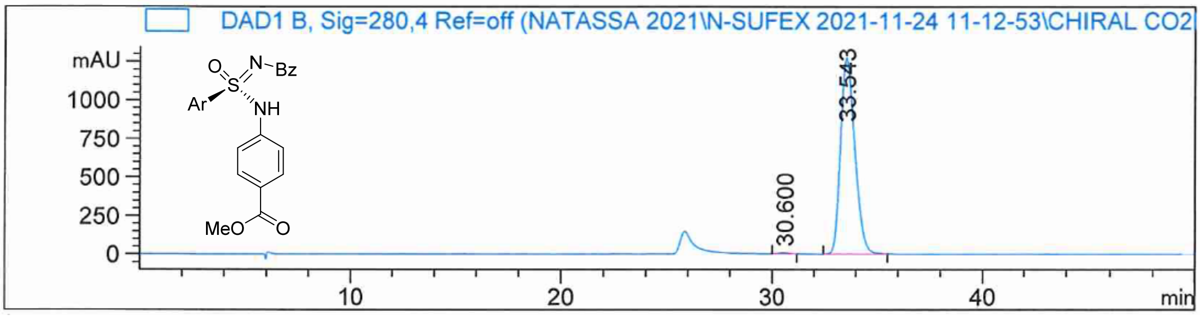


```
Signal 1: DAD1 B, Sig=280,4 Ref=off

Peak RetTime Sig Type    Area      Height     Area
  #   [min]             [mAU*s]     [mAU]       %
----|-------|---|----|----------|----------|--------|
   1  30.600   1  VV    235.18607    6.77477   0.3829
   2  33.543   1  VB   6.11885e4  1277.06152  99.6171

Totals :               6.14237e4  1283.83629
```

(*rac*)-2l

HPLC (Chiralpak IA, *n*-hex/IPA/formic acid = 90:10:0.1%, flow rate = 0.5 ml/min, l = 240 nm)

tR = 90.0 min, 97.4 min

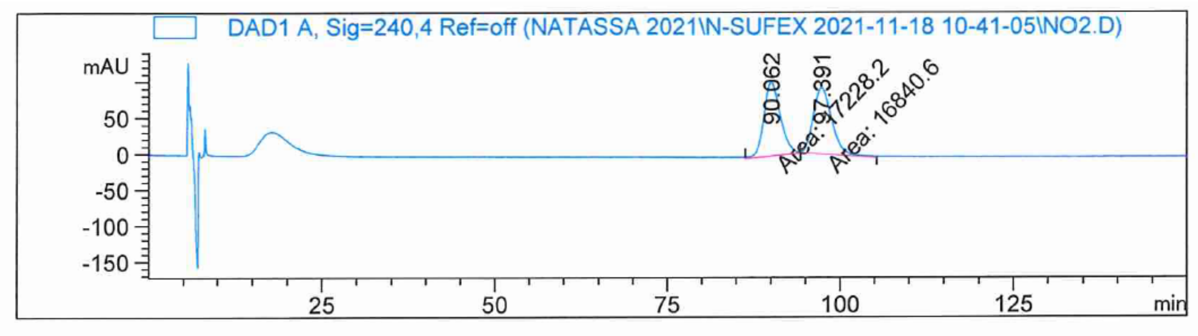

Signal 1: DAD1 A, Sig=240,4 Ref=off

Peak #	RetTime [min]	Sig	Type	Area [mAU*s]	Height [mAU]	Area %
1	90.062	1	MM	1.72282e4	103.85598	50.5689
2	97.391	1	MM	1.68406e4	92.43372	49.4311
Totals :				3.40689e4	196.28970	

(*S*)-2l, *ee* = >99%

tR = 90.1 min

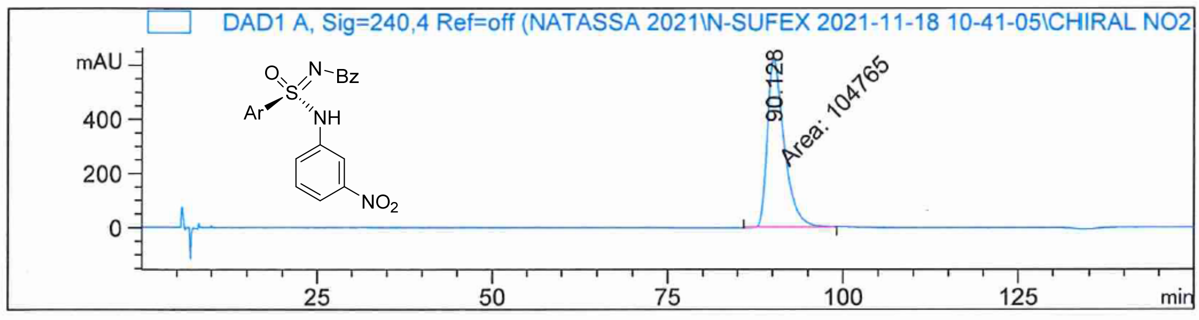

Signal 1: DAD1 A, Sig=240,4 Ref=off

Peak #	RetTime [min]	Sig	Type	Area [mAU*s]	Height [mAU]	Area %
1	90.128	1	MM	1.04765e5	625.08698	100.0000
Totals :				1.04765e5	625.08698	

X-Ray crystallographic data of (*S*)-2b and (*S*)-2d

The selected crystal was mounted onto a goniometer head and cooled to 150K with an Oxford Cryosystem. Intensity data were collected on a SuperNova, Dual, Cu at zero, EosS2 using a Cu microfocus source (λ = 1.54184) Å.

Unit cell determination, data collection, data reduction and a symmetry-related (multi-scan) absorption correction were performed using the CrysAlisPro software.[5]

The structure was solved with SHELXT and refined by a full-matrix least-squares procedure based on F^2 (Shelxl-2019/2).[6]

All non-hydrogen atoms were refined anisotropically. Hydrogen atoms were placed onto calculated positions and refined using a riding model.

Additional programs used for analyzing data and their graphical manipulation included: SHELXle,[7] Mercury.[8]

Single crystals of **(*S*)-2b** were obtained by recrystallization from Hexane/EtOAc, and the absolute configuration was confirmed by X-ray crystallography.

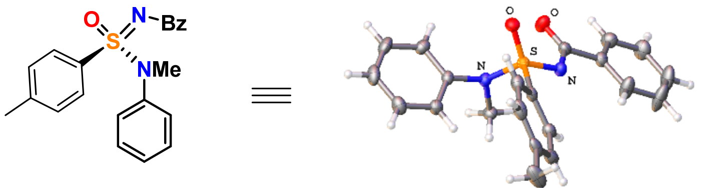

(*S*)-2b

Figure S34. X-Ray crystallography structure of **(*S*)-2b**

Table S7. Crystal data and structure refinement for **(*S*)-2b**

Identification code	**(*S*)-2b**	
Empirical formula	$C_{21}H_{20}N_2O_2S$	
Formula weight	364.45	
Temperature	150.01(10) K	
Wavelength	1.54184 Å	
Crystal system	Monoclinic	
Space group	$P2_1$	
Unit cell dimensions	a = 6.67070(8) Å	α= 90°.
	b = 7.74064(10) Å	β= 96.1236(13)°.
	c = 18.0469(3) Å	γ = 90°.
Volume	926.54(2) $Å^3$	
Z	2	
Density (calculated)	1.306 Mg/m^3	
Absorption coefficient	1.689 mm^{-1}	
F(000)	384	
Crystal size	0.161 x 0.145 x 0.078 mm^3	
Theta range for data collection	4.929 to 72.975°.	
Index ranges	-8<=h<=8, -9<=k<=9, -21<=l<=22	
Reflections collected	12759	
Independent reflections	3646 [R(int) = 0.0285]	
Completeness to theta = 67.684°	99.90%	
Absorption correction	Semi-empirical from equivalents	
Max. and min. transmission	1.00000 and 0.81302	
Refinement method	Full-matrix least-squares on F^2	
Data / restraints / parameters	3646 / 1 / 237	
Goodness-of-fit on F^2	1.042	
Final R indices [I>2sigma(I)]	R1 = 0.0309, wR2 = 0.0818	
R indices (all data)	R1 = 0.0314, wR2 = 0.0822	
Absolute structure parameter	0.017(11)	
Extinction coefficient	n/a	
Largest diff. peak and hole	0.238 and -0.300 e.$Å^{-3}$	

Single crystals of **(*S*)-2d** were obtained by recrystallization from Hexane/EtOAc, and the absolute configuration was confirmed by X-ray crystallography.

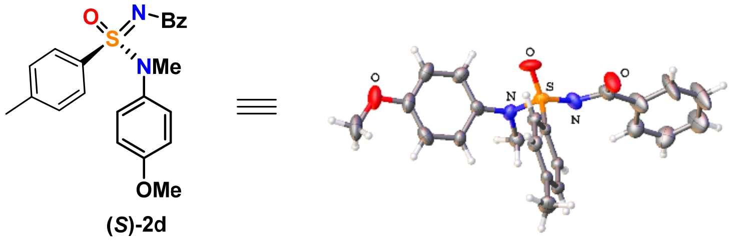

Figure S35. X-Ray crystallography structure of **(*S*)-2d**

Table S8. Crystal data and structure refinement for **(*S*)-2d**

Identification code	**(*S*)-2d**	
Empirical formula	$C_{22}H_{22}N_2O_3S$	
Formula weight	394.47	
Temperature	150.00(10) K	
Wavelength	1.54184 Å	
Crystal system	Orthorhombic	
Space group	$P2_12_12_1$	
Unit cell dimensions	a = 6.00677(4) Å	α= 90°.
	b = 15.56082(11) Å	β= 90°.
	c = 21.55575(16) Å	γ = 90°.
Volume	2014.82(2) Å^3	
Z	4	
Density (calculated)	1.300 Mg/m^3	
Absorption coefficient	1.632 mm^{-1}	
F(000)	832	
Crystal size	0.261 x 0.116 x 0.075 mm^3	
Theta range for data collection	3.503 to 72.878°.	
Index ranges	-7<=h<=7, -18<=k<=19, -26<=l<=25	
Reflections collected	29306	
Independent reflections	4017 [R(int) = 0.0296]	
Completeness to theta = 67.684°	100.00%	
Absorption correction	Semi-empirical from equivalents	
Max. and min. transmission	1.00000 and 0.75934	
Refinement method	Full-matrix least-squares on F^2	
Data / restraints / parameters	4017 / 60 / 336	
Goodness-of-fit on F^2	1.059	
Final R indices [I>2sigma(I)]	R1 = 0.0309, wR2 = 0.0849	
R indices (all data)	R1 = 0.0313, wR2 = 0.0853	
Absolute structure parameter	0.000(4)	
Extinction coefficient	n/a	
Largest diff. peak and hole	0.181 and -0.311 e.Å^{-3}	

Computational studies

All DFT calculations were performed using the Gaussian 16 (version c01) package.[9] The ωB97XD functional[10] combined with the 6-311+G(d,p) basis set as implemented in there was used for geometry optimizations and vibrational frequency calculations. The transition states were confirmed by vibrational frequency analysis with only one imaginary vibration along the reaction coordinate and the reaction paths were followed using intrinsic reaction coordinate (IRC) calculations. Reaction energies reported in this study are Gibbs free energies at 353 K and ambient pressure, including solvation correction with the PCM solvent model.

Table S9. Summary of reaction Gibbs free energies (353.15K, 1atm).

Entry	G (Hartree)	ΔG (kcal/mol)
$[Ca(NTf_2)_2$(*t*-Butanol)$_2$]+ **1** + 2×$PhNH_2$	-6578.701037	4.9
$[Ca(NTf_2)_2$(**1**)] + 2×(*t*-Butanol) + 2×$PhNH_2$	-6578.708851	0.0
$[Ca(NTf_2)_2$(**1**)]•$PhNH_2$ + 2×(*t*-Butanol) + $PhNH_2$	-6578.703637	3.3
**TS1**	-6578.681348	17.3
$[Ca(NTf_2)_2(F)^-$(**2•H**$^+$)] + 2×(*t*-Butanol) + $PhNH_2$	-6578.688877	12.5
$[Ca(NTf_2)_2(F)$(**2**)$]^-[H_3NPh]^+$ + 2×(*t*-Butanol)	-6578.710923	-1.3
$[Ca(NTf_2)_2(F)$(*t*-Butanol)$]^-[H_3NPh]^+$ + **2** + *t*-Butanol	-6578.720756	-7.5

Table S10. Gibbs free energies (353.15K, 1atm) of substrates, solvent, and product.

Entry	G (Hartree)
$PhNH_2$	-287.50532
t-Butanol	-233.58102
**1**	-1204.36435
**2**	-1391.40879
$Ca(NTf_2)_2(PhNH_2)_2$	-4907.17229
$Ca(NTf_2)_2$(*t*-Butanol)$_2$	-4799.32605
$[Ca(NTf_2)_2$(**1**)]	-5536.53618
$[Ca(NTf_2)_2$(**2**)]	-5723.59299

Table S11. Coordination Gibbs free energy (353.15 K, 1 atm) between $Ca(NTf_2)_2$ and various nucleophiles.

$$[Ca(NTf_2)_2(PhNH_2)_2] + \text{Nucleophile} \rightleftarrows [Ca(NTf_2)_2(\text{Nucleophile})] + 2\ PhNH_2$$

Nucleophile	ΔG (kcal/mol)
2 × $PhNH_2$	0.00
2 × *t*-Butanol	-1.5
**1**	-6.4
**2**	-12.7

Table S12. Distances of the most relevant bonds broken and formed during TS1 (Å).

	$[Ca(NTf_2)_2(\mathbf{1})]\bullet PhNH_2$	**TS1**	$[Ca(NTf_2)_2(F)^-(\mathbf{2\bullet H^+})]$
$Ca\text{-}O_{NTf}$	2.37, 2.39, 2.39, 2.40	2.41, 2.41, 2.50, 2.54	2.42, 2.43, 2.44, 2.51
Ca-(O=C)	2.42	2.42	2.49
Ca-(O=S)	2.46	2.54	2.58
Ca-F	4.08	2.27	2.10
S-N	3.25	1.97	1.91
S-F	1.63	2.23	3.97

Coordinates of all DFT model compounds:

PhNH₂

H	-2.77425	0.83462	0.27392
C	-0.22087	1.20427	-0.00535
C	-0.93591	0.	-0.00922
C	-0.22085	-1.20428	-0.0054
C	1.16786	-1.19854	0.00361
C	1.87579	0.00001	0.00757
C	1.16785	1.19854	0.00362
H	-0.76046	2.14614	-0.00999
H	-0.76046	-2.14614	-0.01009
H	1.70081	-2.1434	0.00842
H	2.95934	0.00001	0.01492
H	1.70079	2.14341	0.00844
N	-2.32782	-0.00005	-0.07562
H	-2.77426	-0.83436	0.27474

Electronic Energy=
-287.58731 (Hartree/Particle)
Zero-point correction=
0.117893
Thermal correction to Energy=
0.125831
Thermal correction to Enthalpy=
0.126950
Thermal correction to Gibbs Free Energy=
0.081991
Sum of electronic and zero-point Energies=
-287.469417
Sum of electronic and thermal Energies=
-287.461479
Sum of electronic and thermal Enthalpies=
-287.460360
Sum of electronic and thermal Free Energies=
-287.505319

***t*-Butanol**

O	2.77067	-0.41118	-1.00566
H	2.50893	-1.3345	-1.03815
C	3.14165	-0.00151	-2.33224
C	1.94437	-0.16321	-3.26911
H	1.63857	-1.21275	-3.32874
H	1.09684	0.42444	-2.90741
H	2.19462	0.17243	-4.27909
C	4.32325	-0.84362	-2.81419
H	5.16867	-0.73927	-2.12948
H	4.04822	-1.90192	-2.86693
H	4.64237	-0.52882	-3.81141
C	3.53697	1.46419	-2.20697
H	3.83757	1.86174	-3.17943
H	2.69615	2.05563	-1.83575
H	4.37426	1.57515	-1.51332

Electronic Energy= -233.681298
(Hartree/Particle)
Zero-point correction=
0.136093
Thermal correction to Energy=
0.145052
Thermal correction to Enthalpy=
0.146170
Thermal correction to Gibbs Free Energy=
0.100281
Sum of electronic and zero-point Energies=
-233.545205
Sum of electronic and thermal Energies=
-233.536245
Sum of electronic and thermal Enthalpies=
-233.535127
Sum of electronic and thermal Free Energies=
-233.581017

1

S	-1.34899	-1.20978	-0.07348
N	-0.73606	-1.90032	-1.28619
C	0.42349	-2.67271	-1.18367
C	0.75948	-3.39536	-2.44389
O	1.10754	-2.74477	-0.1819
C	1.91525	-4.17803	-2.47063
C	2.26276	-4.86635	-3.62393
C	1.45707	-4.77756	-4.75619
C	0.30385	-3.99958	-4.73344
C	-0.04569	-3.30805	-3.58073
O	-1.68259	-1.88104	1.15762
C	-2.69197	-0.24638	-0.67605
C	-2.66237	0.24604	-1.97673
C	-3.73197	1.01573	-2.40851
C	-4.7978	1.27636	-1.55102
C	-4.80548	0.77209	-0.25507
C	-3.74382	0.00049	0.19812
H	2.53215	-4.23821	-1.58218
H	3.16088	-5.47261	-3.64177
H	1.72889	-5.31592	-5.65716
H	-0.3234	-3.93114	-5.61455
H	-0.94174	-2.70047	-3.557
H	-1.82936	0.02247	-2.63028
H	-5.6392	0.97431	0.40556
H	-3.73593	-0.405	1.20186
H	-3.73448	1.40865	-3.41755
H	-5.63073	1.87632	-1.89804
F	-0.32536	-0.01214	0.38296

Electronic Energy= -1204.51367
(Hartree/Particle)
Zero-point correction=
0.206282
Thermal correction to Energy=
0.226955
Thermal correction to Enthalpy=
0.228073
Thermal correction to Gibbs Free Energy=
0.149316
Sum of electronic and zero-point Energies=
-1204.307388
Sum of electronic and thermal Energies=
-1204.286715

Sum of electronic and thermal Enthalpies=
-1204.285597
Sum of electronic and thermal Free Energies=
-1204.364354

2

S	-2.09384	-1.88632	-0.50586
N	-1.18788	-3.08129	-0.96339
C	0.15002	-2.81815	-1.14687
C	0.9301	-3.99779	-1.64247
O	0.68995	-1.73747	-0.94384
C	2.2961	-3.83895	-1.87838
C	3.05435	-4.90512	-2.34265
C	2.45152	-6.1382	-2.57503
C	1.0896	-6.3019	-2.34132
C	0.32968	-5.23546	-1.87711
O	-1.97746	-1.29693	0.81813
C	-2.1068	-0.56828	-1.70998
C	-1.99891	-0.88965	-3.05815
C	-2.09325	0.131	-3.9931
C	-2.30257	1.44271	-3.57511
C	-2.41278	1.74275	-2.22216
C	-2.31587	0.73182	-1.27307
H	2.75183	-2.87378	-1.69311
H	4.11523	-4.77578	-2.52363
H	3.04334	-6.97145	-2.93713
H	0.61902	-7.26183	-2.52051
H	-0.73021	-5.35698	-1.69218
H	-1.83865	-1.91495	-3.37119
H	-2.57404	2.76458	-1.90093
H	-2.39543	0.94582	-0.21498
H	-2.00378	-0.09815	-5.04798
H	-2.37797	2.23535	-4.31027
C	-4.73737	-1.78175	-0.65104
C	-5.45753	-1.61447	-1.83118
C	-5.13147	-1.13408	0.51581
C	-6.58401	-0.80217	-1.83908
H	-5.13476	-2.11627	-2.73706
C	-6.24467	-0.30252	0.49171
H	-4.56808	-1.28324	1.4282
C	-6.97441	-0.13782	-0.68082
H	-7.14881	-0.6789	-2.75571
H	-6.54943	0.20718	1.39828
H	-7.84764	0.50394	-0.69147
H	-3.54978	-3.31294	-1.38031
N	-3.58269	-2.6263	-0.63464

Electronic Energy= -1391.65521
(Hartree/Particle)
Zero-point correction=
0.312908
Thermal correction to Energy=
0.340437
Thermal correction to Enthalpy=
0.341555
Thermal correction to Gibbs Free Energy=
0.246420
Sum of electronic and zero-point Energies=
-1391.342302
Sum of electronic and thermal Energies=
-1391.314773
Sum of electronic and thermal Enthalpies=
-1391.313655
Sum of electronic and thermal Free Energies=
-1391.408790

$[Ca(NTf_2)_2(PhNH_2)_2]$

Ca	0.67102	0.00274	1.52815
N	2.48343	-3.38101	2.78864
N	0.5735	3.58803	-0.26398
S	0.90267	-3.41865	2.82513
O	0.25362	-2.15435	2.4524
C	0.4983	-3.63439	4.63813
S	3.44412	-2.15136	2.50233
O	2.75532	-0.88633	2.21497
C	4.25451	-1.82337	4.15661
S	1.97296	2.83834	-0.25714
O	1.89542	1.4004	0.03836
C	2.47946	2.90763	-2.05423
S	-0.85077	3.01871	0.12312
O	-0.82335	1.70192	0.77322
C	-1.68703	2.68086	-1.5149
O	0.40344	-4.62045	2.20263
O	4.51791	-2.54356	1.62509
O	2.96811	3.6069	0.44964
O	-1.64511	4.04818	0.74547
F	1.00164	-4.77803	5.07118
F	-0.81909	-3.64732	4.78007
F	1.00438	-2.63784	5.3511
F	4.69412	-2.95402	4.68303
F	3.38606	-1.25896	4.98433
F	5.27339	-0.99742	3.96861
F	1.61236	2.23726	-2.79966
F	3.67728	2.35645	-2.17495
F	2.5265	4.16509	-2.45745
F	-1.06735	1.68526	-2.14282
F	-1.65199	3.76167	-2.27355
F	-2.9448	2.33369	-1.2914
C	-0.32002	1.45886	4.309
C	-0.52556	0.41207	5.20518
C	-1.33792	2.38035	4.07276
C	-1.74195	0.29323	5.86592
H	0.26657	-0.30537	5.38493
C	-2.55099	2.25422	4.73865
H	-1.1854	3.19051	3.36774
C	-2.76071	1.21199	5.63613
H	-1.89069	-0.52312	6.56363
H	-3.33653	2.97753	4.55145
H	-3.70886	1.11758	6.15207
N	0.89921	1.52728	3.56995
H	1.11278	2.48039	3.29662
H	1.68476	1.18547	4.11414
C	0.96825	-1.93698	-1.16211
C	1.09812	-3.28614	-0.84244
C	1.94217	-1.31279	-1.93897
C	2.20025	-4.00253	-1.29432
H	0.34316	-3.77774	-0.23899
C	3.03674	-2.03751	-2.39212
H	1.84353	-0.26091	-2.17852
C	3.17454	-3.38388	-2.06944
H	2.29455	-5.05048	-1.03347
H	3.78919	-1.54221	-2.99534
H	4.03478	-3.94401	-2.41621
N	-0.10271	-1.16659	-0.61574
H	-0.41868	-0.45484	-1.2656
H	-0.90152	-1.75075	-0.39122

Zero-point correction=
0.352584 (Hartree/Particle)
Thermal correction to Energy=
0.413850
Thermal correction to Enthalpy=
0.414968
Thermal correction to Gibbs Free Energy=
0.238497
Sum of electronic and zero-point Energies=
-4907.058203
Sum of electronic and thermal Energies=
-4906.996937
Sum of electronic and thermal Enthalpies=
-4906.995818
Sum of electronic and thermal Free Energies=
-4907.172290

[Ca(NTf$_2$)$_2$(*t*-BuOH)$_2$]

Ca	0.83231	-0.198	1.61968
N	1.70424	-3.69539	3.39641
N	0.39726	3.35778	-0.21679
S	0.27354	-3.11534	3.75524
O	-0.05117	-1.85446	3.07688
C	0.38873	-2.6318	5.55708
S	2.90879	-2.96086	2.66902
O	2.67878	-1.53548	2.39129
C	4.25069	-2.96127	3.97092
S	1.87886	2.79684	-0.10466
O	1.96671	1.35078	0.15108
C	2.52184	2.99392	-1.84871
S	-0.95896	2.58356	0.05414
O	-0.79328	1.33676	0.81219
C	-1.53536	2.00585	-1.62795
O	-0.70886	-4.1685	3.70833
O	3.42918	-3.76946	1.59291
O	2.70203	3.66263	0.70485
O	-1.96799	3.50746	0.50554
F	0.7586	-3.67654	6.27932
F	-0.79922	-2.20973	5.96422
F	1.27399	-1.65762	5.70768
F	4.47543	-4.19756	4.38083
F	3.89376	-2.21196	5.00244
F	5.3543	-2.46507	3.43125
F	1.82331	2.2418	-2.68487
F	3.79035	2.61244	-1.8709
F	2.43375	4.26036	-2.21596
F	-0.66982	1.13376	-2.12479
F	-1.63663	3.03925	-2.44732
F	-2.71795	1.42295	-1.50049
C	1.42575	2.12281	4.31477
C	1.01612	1.66473	5.70965
H	1.8217	1.09997	6.18662
H	0.79324	2.53041	6.33771
H	0.12735	1.03198	5.66031
C	0.28415	2.84802	3.62015
H	-0.02557	3.70491	4.22141
H	0.59807	3.22632	2.64502
H	-0.58053	2.19213	3.49411
C	2.68423	2.98165	4.34792
H	3.50946	2.444	4.82496
H	2.97989	3.25939	3.334
H	2.50154	3.89356	4.92135
O	1.70209	0.94124	3.50683
H	2.45368	0.47162	3.88149
C	0.67153	-2.4935	-1.19587
C	1.97765	-3.12669	-1.66142
H	1.77659	-4.07729	-2.1613
H	2.49362	-2.47499	-2.37321
H	2.63477	-3.31254	-0.80954
C	-0.03319	-3.37298	-0.17443
H	0.64085	-3.63934	0.64297
H	-0.9141	-2.87026	0.23328
H	-0.359	-4.30025	-0.64931
C	-0.24782	-2.17095	-2.3685
H	-1.16864	-1.70075	-2.01706
H	0.24267	-1.49466	-3.07443
H	-0.50632	-3.0872	-2.90442
O	0.98834	-1.24802	-0.50796
H	1.45938	-0.66752	-1.11428

Electronic Energy= -4799.60285
(Hartree/Particle)
Zero-point correction=
0.389129
Thermal correction to Energy=
0.452189
Thermal correction to Enthalpy=
0.453308
Thermal correction to Gibbs Free Energy=
0.276811
Sum of electronic and zero-point Energies=
-4799.213727
Sum of electronic and thermal Energies=
-4799.150667
Sum of electronic and thermal Enthalpies=
-4799.149548
Sum of electronic and thermal Free Energies=
-4799.326045

[Ca(NTf$_2$)$_2$(1)]

S	-0.66349	0.28757	-1.05256
N	-0.43518	-1.11871	-1.60776
C	0.80144	-1.72189	-1.47457
C	0.82612	-3.14986	-1.84129
O	1.80188	-1.13519	-1.05515
C	2.02975	-3.84784	-1.72167
C	2.07247	-5.20416	-2.00343
C	0.91691	-5.86468	-2.41068
C	-0.28399	-5.17105	-2.53443
C	-0.33354	-3.81534	-2.2486
O	-0.02943	0.74252	0.17098
C	-2.38295	0.59666	-1.04526
C	-3.1615	0.12838	-2.10061
C	-4.51951	0.39795	-2.06803
C	-5.06565	1.11052	-1.00201
C	-4.26392	1.55882	0.04126
C	-2.89791	1.30837	0.0315
H	2.92076	-3.32709	-1.39652
H	3.0041	-5.74707	-1.90059
H	0.95061	-6.92622	-2.62817
H	-1.18229	-5.69018	-2.84633
H	-1.26557	-3.27047	-2.32965
H	-2.71989	-0.43341	-2.91362
H	-4.69818	2.10213	0.87089

H	-2.26258	1.6316	0.84747
H	-5.15303	0.04782	-2.87307
H	-6.13	1.31252	-0.98429
Ca	2.16928	-0.05994	1.05819
N	1.85188	-3.99259	2.08339
N	0.55059	3.27424	2.62841
S	0.4917	-3.17408	2.07788
O	0.59544	-1.85673	1.4428
C	0.14973	-2.77135	3.87195
S	3.31503	-3.40686	1.89636
O	3.39114	-1.93791	1.87508
C	4.14638	-3.88322	3.50131
S	0.05763	1.87825	3.20616
O	0.96448	0.75468	2.94351
C	0.18432	2.1317	5.05245
S	1.81744	3.53089	1.71074
O	2.30718	2.33772	1.0113
C	3.21184	3.93757	2.88989
O	-0.59064	-4.03243	1.66802
O	4.04753	-4.11814	0.87596
O	-1.35144	1.68368	2.95606
O	1.61581	4.72728	0.93306
F	0.0979	-3.88799	4.58261
F	-1.01198	-2.14047	3.95576
F	1.10832	-1.99183	4.34982
F	4.10649	-5.19694	3.65006
F	3.54471	-3.30557	4.52841
F	5.40951	-3.48295	3.45224
F	1.442	2.35431	5.40198
F	-0.25085	1.03535	5.65616
F	-0.56194	3.16162	5.41601
F	3.54872	2.85101	3.57377
F	2.8364	4.88712	3.73101
F	4.25918	4.35382	2.19272
F	-0.15305	1.33668	-2.18011

Electronic Energy= -5536.73876 (Hartree/Particle)
Zero-point correction= 0.319113
Thermal correction to Energy= 0.382693
Thermal correction to Enthalpy= 0.383812
Thermal correction to Gibbs Free Energy= 0.202584
Sum of electronic and zero-point Energies= -5536.419650
Sum of electronic and thermal Energies= -5536.356069
Sum of electronic and thermal Enthalpies= -5536.354951
Sum of electronic and thermal Free Energies= -5536.536179

[Ca(NTf$_2$)$_2$(2)]

S	-1.88048	-1.485	-0.7941
N	-0.97743	-2.66051	-1.25061
C	0.28597	-2.87255	-0.77394
C	0.90944	-4.13103	-1.26968
O	0.88881	-2.13465	0.00524
C	2.214	-4.43068	-0.87623
C	2.81993	-5.59957	-1.31536
C	2.12668	-6.47466	-2.14601
C	0.82525	-6.17838	-2.54111
C	0.2168	-5.00946	-2.10535
O	-1.60641	-0.69577	0.40791
C	-2.23699	-0.41701	-2.15684
C	-2.10847	-0.90679	-3.45007
C	-2.47197	-0.07708	-4.50172
C	-2.95961	1.20105	-4.2466
C	-3.08042	1.66722	-2.9412
C	-2.71704	0.85602	-1.87608
H	2.7507	-3.74546	-0.23372
H	3.83431	-5.82728	-1.00933
H	2.60135	-7.38746	-2.48816
H	0.28582	-6.85842	-3.19008
H	-0.79425	-4.77155	-2.41129
H	-1.72098	-1.90199	-3.62783
H	-3.45136	2.66637	-2.74967
H	-2.78844	1.20792	-0.8552
H	-2.37002	-0.42942	-5.52069
H	-3.2416	1.8423	-5.07336
C	-4.5325	-1.54747	-0.45325
C	-5.44378	-1.68629	-1.49594
C	-4.7727	-0.65868	0.58823
C	-6.61452	-0.93966	-1.48339
H	-5.23819	-2.36919	-2.31352
C	-5.93657	0.09907	0.58065
H	-4.05487	-0.55157	1.3903
C	-6.86003	-0.04225	-0.44903
H	-7.32875	-1.05155	-2.29039
H	-6.12128	0.7957	1.38971
H	-7.77018	0.54579	-0.44664
H	-3.36525	-3.09643	-1.12711
N	-3.31926	-2.32579	-0.46385
Ca	0.70776	0.15445	0.80211
N	2.53138	-2.89124	2.65406
N	0.15662	3.58981	3.01124
S	1.02937	-2.65239	3.05975
O	0.49487	-1.31275	2.81916
C	1.05677	-2.83434	4.91934
S	3.52923	-1.84704	1.97758
O	2.91376	-0.5978	1.53681
C	4.64143	-1.31958	3.38526
S	-0.69745	2.32392	3.43274
O	-0.76048	1.27372	2.41502
C	0.24852	1.53804	4.8406
S	1.298	3.61806	1.89764
O	1.69738	2.30361	1.38932
C	2.77028	4.19155	2.89607
O	0.18725	-3.76544	2.63224
O	4.39958	-2.54573	1.06163
O	-1.94177	2.75186	4.02912
O	1.06252	4.67058	0.93728
F	1.55557	-4.01601	5.24657
F	-0.18983	-2.74568	5.36624
F	1.78885	-1.88028	5.46798
F	5.12639	-2.3863	4.00385
F	3.96872	-0.57849	4.2511
F	5.64278	-0.60789	2.88694
F	1.42239	1.10083	4.41401
F	-0.45387	0.51682	5.31721
F	0.43728	2.42395	5.80909
F	3.05292	3.31811	3.85068
F	2.51303	5.36981	3.4434
F	3.81238	4.30341	2.08381

Electronic Energy= -5723.89103
(Hartree/Particle)
Zero-point correction=
0.425591
Thermal correction to Energy=
0.497242
Thermal correction to Enthalpy=
0.498360
Thermal correction to Gibbs Free Energy=
0.298037
Sum of electronic and zero-point Energies=
-5723.465440
Sum of electronic and thermal Energies=
-5723.393789
Sum of electronic and thermal Enthalpies=
-5723.392671
Sum of electronic and thermal Free Energies=
-5723.592994

[Ca(NTf$_2$)$_2$(1)]•PhNH$_2$

S	2.99653	-0.59845	-1.80357
N	3.39056	0.86436	-1.6272
C	2.43532	1.85142	-1.49644
C	2.98014	3.19637	-1.22212
O	1.22168	1.65547	-1.58759
C	2.09838	4.27863	-1.1783
C	2.57945	5.55031	-0.90939
C	3.93854	5.7449	-0.67709
C	4.81918	4.66798	-0.71776
C	4.34307	3.39394	-0.99132
O	1.70593	-1.12942	-1.39879
C	4.30156	-1.64751	-1.3026
C	5.58434	-1.1363	-1.14819
C	6.58704	-2.01708	-0.77326
C	6.29764	-3.36442	-0.57217
C	5.00353	-3.84628	-0.72938
C	3.98041	-2.98428	-1.09476
H	1.04275	4.11906	-1.35618
H	1.89551	6.38992	-0.87726
H	4.31265	6.73985	-0.46395
H	5.87596	4.82175	-0.53493
H	5.02076	2.55038	-1.02263
H	5.78401	-0.08484	-1.30504
H	4.78127	-4.89038	-0.551
H	2.96201	-3.33662	-1.19374
H	7.59627	-1.64917	-0.63722
H	7.08967	-4.04285	-0.27812
C	3.21955	-1.5208	1.83411
C	4.29592	-2.27864	2.30526
C	1.9648	-2.13028	1.71405
C	4.12316	-3.61722	2.63168
H	5.27498	-1.81823	2.39573
C	1.80215	-3.46826	2.04358
H	1.12696	-1.55658	1.33479
C	2.87837	-4.22473	2.50199
H	4.97301	-4.19047	2.98706
H	0.82497	-3.9257	1.92984
H	2.74749	-5.27072	2.75418
H	2.6264	0.42065	1.54425
H	4.27034	0.21558	1.69899
N	3.40452	-0.20932	1.40068
Ca	-0.49826	-0.04981	-1.50024
N	-0.72216	3.0885	0.90094
N	-3.11083	-3.10407	-0.8379
S	0.05849	1.83705	1.47149
O	-0.22184	0.56064	0.80106
C	-0.65512	1.62974	3.18508
S	-1.71872	3.14434	-0.33147
O	-1.76245	1.92198	-1.13973
C	-3.41918	3.20891	0.44301
S	-1.86029	-3.01608	0.13229
O	-0.80386	-2.11411	-0.33856
C	-2.50111	-2.179	1.6739
S	-3.49615	-2.07813	-1.98975
O	-2.6944	-0.84706	-2.01573
C	-5.1911	-1.50373	-1.44879
O	1.45192	2.15643	1.70158
O	-1.56733	4.40089	-1.02467
O	-1.4695	-4.33785	0.55591
O	-3.70886	-2.75395	-3.24745
F	-0.46203	2.73882	3.88089
F	-0.02902	0.62255	3.77606
F	-1.94986	1.36941	3.12354
F	-3.4644	4.16858	1.35348
F	-3.70333	2.0479	1.01073
F	-4.30813	3.46042	-0.50751
F	-2.88252	-0.94526	1.38196
F	-1.53318	-2.13833	2.57769
F	-3.53089	-2.85362	2.16212
F	-5.12361	-0.90119	-0.27309
F	-6.0087	-2.54059	-1.36631
F	-5.65096	-0.65031	-2.35308
F	3.01785	-0.85812	-3.41246

Electronic Energy= -5824.34693
(Hartree/Particle)
Zero-point correction=
0.438638
Thermal correction to Energy=
0.512340
Thermal correction to Enthalpy=
0.513458
Thermal correction to Gibbs Free Energy=
0.310654
Sum of electronic and zero-point Energies=
-5823.908301
Sum of electronic and thermal Energies=
-5823.834599
Sum of electronic and thermal Enthalpies=
-5823.833480
Sum of electronic and thermal Free Energies=
-5824.036284

TS1

Imaginary frequency: -111.79 cm^{-1}

S	2.86554	-0.30654	-0.63332
N	3.18905	1.16124	-0.96988
C	2.22109	2.12123	-1.10671
C	2.74429	3.48969	-1.32645
O	1.0129	1.89514	-1.04822
C	1.83796	4.55153	-1.35107
C	2.29742	5.84485	-1.55441
C	3.65686	6.08156	-1.73662

C	4.5618	5.02409	-1.71186
C	4.10884	3.72952	-1.50297
O	1.66803	-0.923	-0.08475
C	3.98408	-1.41514	-1.40187
C	5.11512	-0.92262	-2.04095
C	6.00662	-1.84452	-2.56935
C	5.75647	-3.2092	-2.4491
C	4.61338	-3.67103	-1.80485
C	3.70685	-2.77009	-1.26562
H	0.78275	4.35907	-1.20098
H	1.5949	6.66972	-1.569
H	4.01283	7.0929	-1.89748
H	5.61979	5.20972	-1.85446
H	4.80821	2.9031	-1.48205
H	5.28466	0.14261	-2.12614
H	4.42462	-4.73342	-1.71783
H	2.81142	-3.10677	-0.75881
H	6.89559	-1.49503	-3.07901
H	6.46011	-3.92003	-2.86557
C	3.95984	-1.48169	1.77979
C	5.17584	-2.14243	1.65697
C	2.88825	-2.03872	2.46675
C	5.32059	-3.39037	2.24661
H	5.99618	-1.69475	1.10682
C	3.04618	-3.28866	3.0484
H	1.94762	-1.50707	2.53572
C	4.25774	-3.96364	2.93738
H	6.26387	-3.91556	2.16167
H	2.21954	-3.7349	3.5873
H	4.37466	-4.93979	3.39272
H	3.10642	0.3898	1.61463
H	4.63956	0.29724	0.97273
N	3.77111	-0.21917	1.11222
Ca	-0.32113	-0.09217	-1.42871
N	-0.6371	2.66132	1.44243
N	-3.30297	-2.90354	-1.34892
S	0.00633	1.27059	1.83926
O	-0.49684	0.12316	1.09102
C	-0.60186	1.00547	3.58444
S	-1.65002	2.90436	0.23337
O	-1.79259	1.79111	-0.70463
C	-3.3229	2.98559	1.0618
S	-2.07267	-3.14583	-0.37833
O	-0.91473	-2.29016	-0.6456
C	-2.69395	-2.58088	1.29197
S	-3.49076	-1.72573	-2.39765
O	-2.47806	-0.66732	-2.33455
C	-5.0533	-0.89646	-1.79289
O	1.45032	1.39131	2.00627
O	-1.44087	4.2296	-0.30344
O	-1.8319	-4.56013	-0.22124
O	-3.82711	-2.24125	-3.70413
F	-0.24169	2.02389	4.34958
F	-0.05186	-0.10982	4.05204
F	-1.91819	0.88348	3.60462
F	-3.30634	3.88516	2.03514
F	-3.64161	1.80292	1.56532
F	-4.23071	3.33399	0.15916
F	-3.03628	-1.30541	1.24748
F	-1.73069	-2.7487	2.18864
F	-3.74836	-3.30461	1.64446
F	-4.85868	-0.34719	-0.60547
F	-6.03376	-1.78308	-1.71251
F	-5.38749	0.04919	-2.66137
F	1.651	-0.41516	-2.49738

Electronic Energy= -5824.32570
(Hartree/Particle)
Zero-point correction=
0.440152
Thermal correction to Energy=
0.513366
Thermal correction to Enthalpy=
0.514484
Thermal correction to Gibbs Free Energy=
0.311707
Sum of electronic and zero-point Energies=
-5823.885550
Sum of electronic and thermal Energies=
-5823.812336
Sum of electronic and thermal Enthalpies=
-5823.811217
Sum of electronic and thermal Free Energies=
-5824.013995

$[Ca(NTf_2)_2(F)^-(2\bullet H^+)]$

S	3.1153	-0.61796	-0.69607
N	3.56498	0.80115	-1.01126
C	2.65921	1.86227	-1.08832
C	3.28875	3.19247	-1.17053
O	1.44572	1.70409	-1.09372
C	2.45849	4.31637	-1.20209
C	3.01771	5.58293	-1.26501
C	4.4017	5.73208	-1.29823
C	5.2305	4.6142	-1.26735
C	4.67763	3.34404	-1.20052
O	1.79765	-1.17132	-0.94433
C	4.38063	-1.741	-1.12336
C	5.68872	-1.28783	-1.28209
C	6.66296	-2.2279	-1.57483
C	6.3231	-3.57371	-1.69599
C	5.00887	-3.99885	-1.5322
C	4.01285	-3.07994	-1.23912
H	1.38335	4.19345	-1.17251
H	2.37521	6.45489	-1.2865
H	4.8364	6.72389	-1.34854
H	6.30688	4.73373	-1.29478
H	5.31722	2.47111	-1.17677
H	5.92697	-0.23649	-1.18897
H	4.75424	-5.0458	-1.63395
H	2.98307	-3.38949	-1.11367
H	7.6879	-1.9086	-1.71335
H	7.09419	-4.29931	-1.92541
C	2.9072	-2.02753	1.74124
C	3.96115	-2.84449	2.13028
C	1.5846	-2.44988	1.77924
C	3.6756	-4.12432	2.58315
H	4.98501	-2.48942	2.08309
C	1.31785	-3.73541	2.22873
H	0.78443	-1.79923	1.44893
C	2.35785	-4.56883	2.62779
H	4.4835	-4.7737	2.89617
H	0.29395	-4.08565	2.26084
H	2.14024	-5.57141	2.97593
H	2.55726	0.03286	1.54851
H	4.15209	-0.41122	1.4587
N	3.20786	-0.71371	1.20844

Ca	-0.36848	0.07526	-1.57548
N	-0.34807	2.80368	1.38505
N	-3.35571	-2.7129	-1.31355
S	0.19212	1.37933	1.76259
O	-0.25175	0.26123	0.92875
C	-0.54165	1.06508	3.45232
S	-1.27853	3.19296	0.1464
O	-1.45798	2.13089	-0.83898
C	-2.97408	3.38171	0.91132
S	-2.15023	-2.88705	-0.30041
O	-0.96956	-2.08236	-0.62267
C	-2.76034	-2.15981	1.3092
S	-3.58966	-1.48603	-2.30367
O	-2.69518	-0.34407	-2.09302
C	-5.26105	-0.86411	-1.74311
O	1.63282	1.41412	2.02496
O	-0.91996	4.5104	-0.31923
O	-1.94906	-4.28751	-0.0154
O	-3.79542	-1.94092	-3.6584
F	-0.19402	2.03827	4.2765
F	-0.06493	-0.08752	3.90163
F	-1.85817	0.99881	3.37382
F	-2.91393	4.20108	1.94955
F	-3.42578	2.20217	1.30665
F	-3.79447	3.88322	0.00017
F	-3.00606	-0.86848	1.16063
F	-1.82636	-2.32313	2.24087
F	-3.86881	-2.77548	1.6947
F	-5.21237	-0.46641	-0.48111
F	-6.16319	-1.82578	-1.86466
F	-5.60623	0.16088	-2.51056
F	0.38238	-0.09916	-3.53232

Electronic Energy= -5824.33218 (Hartree/Particle)
Zero-point correction= 0.440054
Thermal correction to Energy= 0.514304
Thermal correction to Enthalpy= 0.515423
Thermal correction to Gibbs Free Energy= 0.310662
Sum of electronic and zero-point Energies= -5823.892132
Sum of electronic and thermal Energies= -5823.817882
Sum of electronic and thermal Enthalpies= -5823.816763
Sum of electronic and thermal Free Energies= -5824.021524

$[Ca(NTf_2)_2(F)(2)]^-[H_3NPh]^+$

S	-1.880475	-1.484995	-0.794100
N	-0.977425	-2.660508	-1.250607
C	0.285973	-2.872547	-0.773940
C	0.909441	-4.131025	-1.269678
O	0.888814	-2.134650	0.005242
C	2.214004	-4.430684	-0.876232
C	2.819934	-5.599574	-1.315357
C	2.126682	-6.474661	-2.146009
C	0.825251	-6.178378	-2.541111
C	0.216804	-5.009456	-2.105347
O	-1.606405	-0.695772	0.407911
C	-2.236990	-0.417014	-2.156844
C	-2.108465	-0.906789	-3.450067
C	-2.471974	-0.077083	-4.501720
C	-2.959608	1.201049	-4.246601
C	-3.080418	1.667220	-2.941202
C	-2.717044	0.856015	-1.876078
H	2.750697	-3.745459	-0.233715
H	3.834310	-5.827284	-1.009328
H	2.601349	-7.387456	-2.488156
H	0.285821	-6.858422	-3.190082
H	-0.794246	-4.771550	-2.411294
H	-1.720983	-1.901990	-3.627828
H	-3.451363	2.666372	-2.749667
H	-2.788437	1.207916	-0.855204
H	-2.370018	-0.429415	-5.520691
H	-3.241601	1.842298	-5.073360
H	-2.921324	-4.536510	2.331759
C	-4.532498	-1.547465	-0.453248
C	-5.443779	-1.686294	-1.495939
C	-4.772703	-0.658677	0.588228
C	-6.614516	-0.939664	-1.483391
H	-5.238190	-2.369193	-2.313523
C	-5.936568	0.099067	0.580645
H	-4.054867	-0.551568	1.390299
C	-6.860031	-0.042247	-0.449026
H	-7.328753	-1.051549	-2.290392
H	-6.121284	0.795700	1.389713
H	-7.770184	0.545794	-0.446640
H	-2.779860	-3.323922	1.204749
H	-3.365247	-3.096430	-1.127109
C	-4.666533	-2.939912	3.368528
C	-3.351753	-2.640310	3.050858
C	-2.751387	-1.451483	3.430351
C	-3.502807	-0.534220	4.156849
C	-4.824545	-0.812245	4.485182
C	-5.404745	-2.014312	4.094550
H	-5.116406	-3.872270	3.046032
H	-1.732549	-1.220970	3.144291
H	-3.056015	0.407650	4.449842
H	-5.405397	-0.088675	5.044598
H	-6.435078	-2.232584	4.347246
N	-2.609924	-3.575009	2.194976
N	-3.319261	-2.325794	-0.463850
H	-1.586612	-3.560934	2.364624
Ca	0.707757	0.154446	0.802111
N	2.531376	-2.891238	2.654056
N	0.156621	3.589810	3.011237
S	1.029370	-2.652391	3.059754
O	0.494873	-1.312752	2.819156
C	1.056774	-2.834336	4.919335
S	3.529228	-1.847037	1.977578
O	2.913759	-0.597803	1.536805
C	4.641429	-1.319583	3.385261
S	-0.697448	2.323916	3.432735
O	-0.760475	1.273723	2.415020
C	0.248524	1.538038	4.840600
S	1.297997	3.618055	1.897642
O	1.697379	2.303607	1.389315
C	2.770276	4.191549	2.896074
O	0.187251	-3.765435	2.632238
O	4.399582	-2.545725	1.061629
O	-1.941766	2.751861	4.029118
O	1.062515	4.670584	0.937278
F	1.555574	-4.016010	5.246569
F	-0.189831	-2.745678	5.366236

F	1.788850	-1.880277	5.467981
F	5.126391	-2.386299	4.003855
F	3.968724	-0.578489	4.251100
F	5.642784	-0.607887	2.886940
F	1.422394	1.100831	4.414011
F	-0.453868	0.516818	5.317211
F	0.437279	2.423947	5.809086
F	3.052924	3.318110	3.850681
F	2.513027	5.369805	3.443402
F	3.812379	4.303409	2.083810
F	0.557112	0.558331	-1.267631

Electronic Energy= -6111.96897
(Hartree/Particle)
Zero-point correction=
0.562015
Thermal correction to Energy=
0.645617
Thermal correction to Enthalpy=
0.646735
Thermal correction to Gibbs Free Energy=
0.420091
Sum of electronic and zero-point Energies=
-6111.406964
Sum of electronic and thermal Energies=
-6111.323363
Sum of electronic and thermal Enthalpies=
-6111.322244
Sum of electronic and thermal Free Energies=
-6111.548889

$[Ca(NTf_2)_2(F)(t\text{-}BuOH)]^-[H_3NPh]^+$

Ca	0.78974	-0.24678	2.34816
N	1.1789	-4.08694	3.67795
N	-0.3867	3.4902	1.07844
S	-0.30442	-3.5663	3.4756
O	-0.4092	-2.28953	2.76005
C	-0.90418	-3.16349	5.20165
S	2.54094	-3.30987	3.44158
O	2.39134	-1.9167	3.01282
C	3.26323	-3.18406	5.16028
S	1.17487	3.25449	1.16277
O	1.59613	1.85064	1.22903
C	1.75586	3.84595	-0.51252
S	-1.52532	2.38939	1.2185
O	-1.09779	1.18158	1.92888
C	-1.83573	1.78599	-0.5252
O	-1.14611	-4.64784	3.02834
O	3.47477	-4.12891	2.70552
O	1.79871	4.1629	2.10854
O	-2.75211	3.02026	1.63191
F	-0.66383	-4.1799	6.01438
F	-2.2086	-2.9337	5.15706
F	-0.28695	-2.08168	5.65392
F	3.397	-4.38975	5.6871
F	2.48322	-2.44776	5.93603
F	4.45576	-2.60751	5.06928
F	1.20078	3.11646	-1.4677
F	3.0732	3.71425	-0.57052
F	1.43009	5.11531	-0.67856
F	-0.76927	1.13107	-0.96554
F	-2.08392	2.81095	-1.32384
F	-2.87729	0.968	-0.51811
C	2.23538	-1.64813	-0.70208
C	3.70223	-1.96191	-0.42764
H	4.04879	-2.76406	-1.08414
H	4.32509	-1.08019	-0.60485
H	3.83307	-2.27764	0.6091
C	1.34668	-2.8484	-0.41386
H	1.50763	-3.21966	0.60004
H	0.29281	-2.5858	-0.53204
H	1.58232	-3.65943	-1.10579
C	2.02686	-1.14459	-2.12686
H	0.97571	-0.90269	-2.29883
H	2.6281	-0.24942	-2.31569
H	2.33061	-1.90869	-2.84601
O	1.80944	-0.60458	0.21153
H	2.20274	0.23317	-0.05242
F	1.43573	0.84958	4.10996
C	5.44151	-0.00731	2.18918
C	6.07613	0.79246	1.24472
C	5.81996	2.1588	1.20329
C	4.92765	2.731	2.10265
C	4.30283	1.91774	3.03403
C	4.54841	0.55491	3.09194
H	5.62983	-1.07372	2.21961
H	6.76726	0.3499	0.53699
H	6.30927	2.78392	0.46601
H	4.71372	3.79306	2.06875
H	4.02913	-0.06271	3.81378
N	3.33072	2.49037	3.9627
H	3.75053	2.70702	4.86554
H	2.92649	3.34895	3.57633
H	2.50326	1.79679	4.10628

Electronic Energy= -4953.99855
(Hartree/Particle)
Zero-point correction=
0.384069
Thermal correction to Energy=
0.448862
Thermal correction to Enthalpy=
0.449980
Thermal correction to Gibbs Free Energy=
0.267606
Sum of electronic and zero-point Energies=
-4953.614487
Sum of electronic and thermal Energies=
-4953.549693
Sum of electronic and thermal Enthalpies=
-4953.548575
Sum of electronic and thermal Free Energies=
-4953.730949

References

1. Liang, D. D.; Streefkerk, D. E.; Jordaan, D.; Wagemakers, J.; Baggerman, J.; Zuilhof, H., Silicon-Free SuFEx Reactions of Sulfonimidoyl Fluorides: Scope, Enantioselectivity, and Mechanism. *Angew. Chem. Int. Ed.* **2020**, *59*, 7494-7500.

2. Chao, Y.; Krishna, A.; Subramaniam, M.; Liang, D.-D.; Pujari, S. P.; Sue, A. C.-H.; Li, G.; Miloserdov, F. M.; Zuilhof, H., Sulfur–Phenolate Exchange: SuFEx-Derived Dynamic Covalent Reactions and Degradation of SuFEx Polymers. *Angew. Chem. Int. Ed.* **2022**, *61*, e202207456.

3. Mukherjee, P.; Woroch, C. P.; Cleary, L.; Rusznak, M.; Franzese, R. W.; Reese, M. R.; Tucker, J. W.; Humphrey, J. M.; Etuk, S. M.; Kwan, S. C.; am Ende, C. W.; Ball, N. D., Sulfonamide Synthesis via Calcium Triflimide Activation of Sulfonyl Fluorides. *Org. Lett.* **2018**, *20*, 3943-3947.

4. Biosca, M.; Pàmies, O.; Diéguez, M., Giving a Second Chance to Ir/Sulfoximine-Based Catalysts for the Asymmetric Hydrogenation of Olefins Containing Poorly Coordinative Groups. *J. Org. Chem.* **2019**, *84*, 8259-8266.

5. CrysAlisPro 1.171.42.49 (Rigaku OD, 2022)

6. Sheldrick, G. M., SHELXL, *Acta Crystallogr.* **2015**, *C71*, 3-8.

7. Hübschle, C. B.; Sheldrick, G. M.; Dittrich, B., ShelXle: a Qt graphical user interface for SHELXL. *J. Appl. Crystallogr.* **2011**, *44*, 1281-1284.

8. Macrae, C. F.; Edgington, P. R.; McCabe, P.; Pidcock, E.; Shields, G. P.; Taylor, R.; Towler, M.; van der Streek, J., Mercury: visualization and analysis of crystal structures, *J. Appl. Crystallogr.* **2006**, *39*, 453-457.

9. Frisch, M. J.; Trucks, G. W.; Schlegel, H. B.; Scuseria, G. E.; Robb, M. A.; Cheeseman, J. R.; Scalmani, G.; Barone, V.; Petersson, G. A.; Nakatsuji, H.; Li, X.; Caricato, M.; Marenich, A. V.; Bloino, J.; Janesko, B. G.; Gomperts, R.; Mennucci, B.; Hratchian, H. P.; Ortiz, J. V.; Izmaylov, A. F.; Sonnenberg, J. L.; Williams; Ding, F.; Lipparini, F.; Egidi, F.; Goings, J.; Peng, B.; Petrone, A.; Henderson, T.; Ranasinghe, D.; Zakrzewski, V. G.; Gao, J.; Rega, N.; Zheng, G.; Liang, W.; Hada, M.; Ehara, M.; Toyota, K.; Fukuda, R.; Hasegawa, J.; Ishida, M.; Nakajima, T.; Honda, Y.; Kitao, O.; Nakai, H.; Vreven, T.; Throssell, K.; Montgomery Jr., J. A.; Peralta, J. E.; Ogliaro, F.; Bearpark, M. J.; Heyd, J. J.; Brothers, E. N.; Kudin, K. N.; Staroverov, V. N.; Keith, T. A.; Kobayashi, R.; Normand, J.; Raghavachari, K.; Rendell, A. P.; Burant, J. C.; Iyengar, S. S.; Tomasi, J.; Cossi, M.; Millam, J. M.; Klene, M.; Adamo, C.; Cammi, R.; Ochterski, J. W.; Martin, R. L.; Morokuma, K.; Farkas, O.; Foresman, J. B.; Fox, D. J. *Gaussian 16 Rev. C.01*, Wallingford, CT, 2016.

10. Chai, J.-D.; Head-Gordon, M., Long-range corrected hybrid density functionals with damped atom–atom dispersion corrections. *Phys. Chem. Chem. Phys.* **2008**, *10*, 6615-6620.
